# Chloroplast acetyltransferase GNAT2 acts as a redox-regulated switch for state transitions in tomato

**DOI:** 10.1186/s43897-025-00164-0

**Published:** 2025-08-06

**Authors:** Xiaoyun Wang, Jianghao Wu, Hongxin Li, Ying Liu, Dexian Han, Danhui Dong, Jialong Zhang, Lixin Zhang, Na Zhang, Yang-Dong Guo

**Affiliations:** 1 College of Horticulture, China Agricultural University, Beijing, 100193 China; 2 College of Plant Science and Technology, Beijing University of Agriculture, Beijing, 102206 China; 3 Henan Key Laboratory of Synthetic Biology and Biomanufacturing, State Key Laboratory of Crop Stress Adaptation and Improvement, School of Life Sciences, Henan University, Jinming Avenue, Kaifeng, 475004 China

**Keywords:** Acetyltransferase, Light-harvesting Complex II, Redox, State Transitions, *Solanum lycopersicum* L

## Abstract

State transition is a dynamic process to balance the amount of light energy received by photosystem I (PSI) and photosystem II (PSII) so as to maintain an optimal photosynthetic yield and to minimize photo-damage in a fluctuating light environment. Recent studies show that chloroplast acetyltransferase participates in the acetylation of photosynthetic proteins and state transitions. However, the exact molecular mechanisms are poorly understood. In this study, we characterized a chloroplast acetyltransferase in *Solanum lycopersicum*, SlGNAT2, and found that mutants lacking this enzyme are deficient in state transitions and retarded in growth under fluctuating light. Acetyltransferase activity assays and fluorescence measurements suggest that ^6^Lys of mature SlLhcb2 protein is a target of SlGNAT2 and might be involved in state transitions. In addition, ^131^Cys-related redox changes of SlGNAT2 affect its acetylation activity on SlLhcb2 and influence the assembly of the PSI-LHCI-LHCII supercomplex. Therefore, we propose that the chloroplast redox state may regulate the activity of SlGNAT2 which in turn acetylates SlLhcb2 and mediates state transitions in higher plants.

**Supplementary Information:**

The online version contains supplementary material available at 10.1186/s43897-025-00164-0.

## Core

Chloroplast redox state regulates SlGNAT2 activity which leads to acetylation SlLhcb2 at ^6^Lys, and facilitates state transitions

## Gene & Accession numbers

Sequence data from this article can be found in the Solanaceae Genomics Network or Arabidopsis Genome Initiative databases under accession numbers: Solyc10g074910 (*GNAT2*), Solyc07g047850 (*Lhcb2*), AT1G32070 (*NSI*), AT3G05010 (*CAND2*).

## Introduction

In a natural environment, plants need to acclimate to constant changes of light quality and quantity due to many factors, like the degree of cloudiness, changes of solar angle or wind-induced movement of leaves, to maintain an optimal photosynthetic yield and to minimize photo-damage (Pearcy 1990; Allahverdiyeva et al. 2015). As an abiotic stress, fluctuating light leads to a decrease of the electron transport rate (ETR) of photosystem I (PSI) and photosystem II (PSII), accumulation of reactive oxygen species (ROS) in chloroplasts, photoinhibition of PSII, and to significantly decreased carbon fixation and productivity in field crops (Slattery et al. 2018; Roeber et al. 2020; Wang et al. 2020b). To rapidly and dynamically respond to the changing light environment, higher plants have evolved sophisticated light acclimation mechanisms. State transitions belong to the fast response mechanisms and play a significant role in rebalancing the light excitation energy between the two photosystems, in photoprotection under fluctuating light, and in regulating the light energy utilization efficiency and stabilizing crop yield (Bellafiore et al. 2005; Grieco et al. 2012).

The phenomenon of state transitions was first detected in *Porphyridium cruentum* (Murata 1969) and *Chlorella pyrenoidosa* (Bonaventura and Myers 1969). It was subsequently found to be a common mechanism in most photosynthetic organisms (Fork and Satoh 1986). Due to the difference of pigment composition, PSII and PSI of higher plants have distinct absorption characteristics (Fork and Satoh 1986). When PSII is preferentially excited (state 2), plastoquinone (PQ) is reduced and binds to the Q_o_ site of the cytochrome *b*_6_*f* complex (Cyt*b*_6_*f*), and activates the serine/threonine-protein kinase STN7 which interacts with the Cyt*b*_6_*f* complex. STN7 is required for the phosphorylation of Lhcb1 and Lhcb2, the subunits of the light-harvesting complex of PSII (LHCII). Upon phosphorylation, one of the LHCII trimers associates with the PSI core subunits, PsaH/L/O, as an extra antenna of PSI, increasing its light-harvesting cross-section (Zito et al. 1999; Bellafiore et al. 2005; Pietrzykowska et al. 2014; Longoni et al. 2015; Shapiguzov et al. 2016; Pan et al. 2018). In the reverse process, over-excitation of PSI by far-red light (state 1) promotes the oxidation of the PQ pool. This leads to the dephosphorylation of the LHCII trimer by the phosphatase PPH1/TAP38 (protein phosphatase 1/thylakoid-associated phosphatase 38), and its return to the PSII supercomplex, thereby rebalancing the excitation energy distribution (Pribil et al. 2010; Shapiguzov et al. 2010).

Protein acetylation of the ε-amino group of lysine (Lys, K) is a widespread post-translational modification of chloroplast proteins. Recently, the acetylation of photosystem proteins catalyzed by *Arabidopsis* NUCLEAR SHUTTLE INTERACTING/serotonin N-acetyltransferase/general control non-repressible 5 (GCN5)-related N-acetyltransferase 2 (NSI/SNAT1/GNAT2; hereafter, NSI) was found to be involved in state transitions (Koskela et al. 2018). The acetylation status of various chloroplast proteins was influenced by NSI, including PSAH-1/PSAH-2, LHCB1.4, PSBP-1, and KEA1/KEA2. Furthermore, plants lacking NSI could not form the state transition supercomplex (PSI-LHCI-LHCII) and were blocked in state 1 even though the phosphorylation of LHCII was normal, indicating the crucial role of NSI in state transitions (Koskela et al. 2018, 2020). But the open questions are whether NSI can directly affect the interactions between LHCII and PSI supercomplex and/or acetylate several photosynthetic proteins to modulate the rearrangement of thylakoid membranes. These rearrangements may promote the assembly of the PSI-LHCI-LHCII complex which is essential for state transitions. As an essential regulator of state transitions, the STN7 kinase phosphorylates several LHCII proteins including Lhcb2 which enhances the affinity of LHCII to PSI (Lemeille et al. 2009; Longoni et al. 2015; Shapiguzov et al. 2016). Thus, whether NSI-mediated lysine acetylation affects the interaction between PSI and LHCII is an important question regarding state transitions.

Under fluctuating light, the photosynthetic electron flow leads to a change of the redox state of the PQ pool. As a key component of the signaling pathway of state transitions, the activity of STN7 kinase is regulated by a dual redox control by PQ and the ferredoxin/thioredoxin system (Rochaix 2013). We recently discovered that lumen thiol oxidoreductase (LTO1) could interact with STN7 and oxidize the conserved lumenal cysteines (Cys, C) of STN7 thereby activating the kinase activity of STN7 (Wu et al. 2021). The equally important roles of NSI-induced acetylation and STN7-induced phosphorylation in state transitions, prompted us to examine whether the activity of NSI and its involvement in state transitions are redox-regulated.

Tomato (*Solanum lycopersicum* L.) is an important crop of high economic and nutritional value as well as a model plant. The improvement of tomato environmental adaptability and photosynthetic efficiency would be helpful for tomato production. Although the regulatory principles explored here are anticipated to operate broadly in plants, this work prioritizes tomato due to its dual role as a model organism and an economic crop with translational potential for these mechanisms. We found that a mutant affected in the chloroplast acetyltransferase SlGNAT2 (hereafter, GNAT2) in tomato, blocked in state 1 and retarded in growth under fluctuating light. In addition, the fruit ripening time in the knockout lines of *GNAT2* was significantly longer compared to wild type (WT) in the greenhouse due to the slower fruit growth. Moreover, lysine acetylome screening was employed to identify the acetylated targets of GNAT2 and WT. We show that ^6^Lys of mature Lhcb2 is acetylated by GNAT2, and that this modification depends on the redox regulation of ^131^Cys. Furthermore, mutating ^131^Cys-GNAT2 affects the assembly of the PSI-LHCI-LHCII supercomplex. Thus, our results reveal a previously unrecognized redox-dependent regulatory process and the acetylation of GNAT2 in tomato during state transitions. These results provide novel insights into the mechanisms of light acclimation of crop plants.

## Results

### GNAT2 is required for state transitions in tomato

**Fig. 1:**
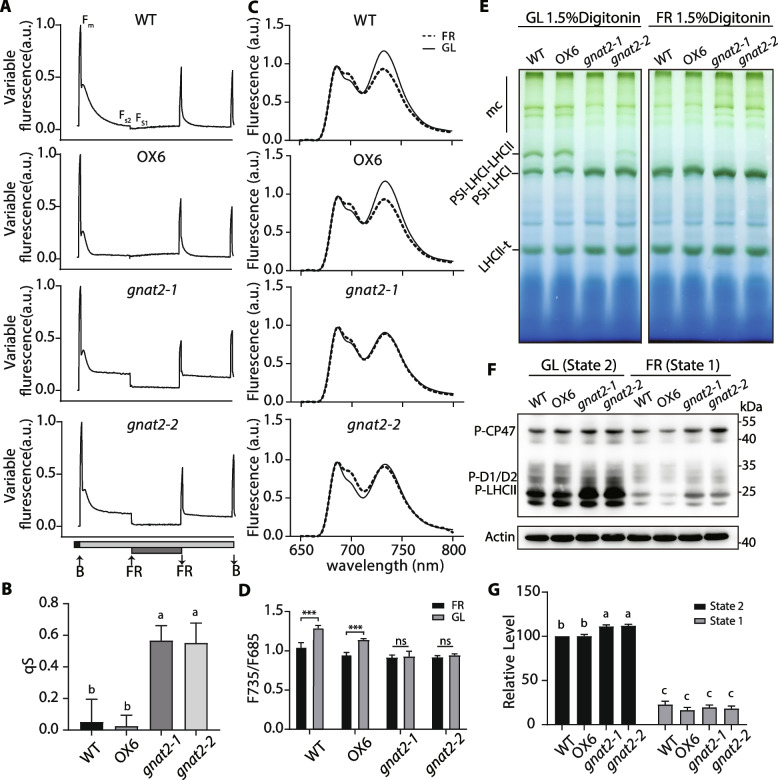
GNAT2 is required for state transitions in tomato. **A** State transitions measured in WT, OX6, *gnat2 - 1* and *gnat2 - 2*. After 30 min dark adaption, blue light (B, 447 nm) and blue light combined with far-red light (FR, 735 nm) were used to induce state 2 and state 1, respectively. F_S1_ and F_S2_ represent the steady-state fluorescence under state 1 and state 2 conditions. Upward arrow indicates light on and downward arrow indicates light off. a.u., arbitrary units. **B** State transitions (qS) were estimated by (F_S2_-F_S1_)/F_S1_ (Wu et al. 2021). The value of qS is inversely related to the extent of state transitions. Values are means ± SD from three independent biological replicates, one-way ANOVA using Tukey’s multiple comparisons. **C** 77 K fluorescence emission spectra of thylakoids from different lines under GL (solid lines) and FR light (dashed lines). **D** The ratio of fluorescence around 735 nm and 685 nm in (**C**). At least four independent measurements were performed. Values are means ± SD, two-tailed student’s *t*-test. **E** Blue native-PAGE analysis of photosystem complexes from different lines. The thylakoid samples were subjected to GL and FR light treatment, and solubilized with 1.5% digitonin. mc, megacomplex; t, trimer. **F** Phosphorylation levels of thylakoid membrane proteins in different lines by immunoblotting (anti-phosphothreonine antiserum, Cell Signaling Technology, 1:10,000). *β*-Actin was used as loading control. **G** The amount of protein of P-LHCII bands was estimated by ImageJ software. The relative levels of each group were normalized with the amount of WT under state 2 condition. The average values (± SEM) from four biological repeats are shown, two-way ANOVA using Tukey’s multiple comparisons test. Different letters indicate significant differences (*P* < 0.05),* P* values were shown in Dataset 2

Although *Arabidopsis* chloroplast acetyltransferase NSI was identified a few years ago (Koskela et al. 2018), the molecular mechanisms underlying its action in state transitions are still largely unknown. In tomato, *GNAT2* displays 60% sequence identity with the *Arabidopsis NSI* gene which contains a GCN5-relative acetyltransferase domain (Fig. S1). We constructed *GNAT2* knockout tomato plants using the CRISPR/Cas9-mediated gene-editing technology, and successfully obtained two individual homozygous lines, *gnat2 - 1* (one base deletion line) and *gnat2 - 2* (one base insertion line) (Fig. S2A-G). The capacity of state transitions was assessed in vivo through measurements of fluorescence from PSII with a pulse amplitude fluorimeter (Jensen et al. 2000; Bellafiore et al. 2005). We observed that state transitions in two knockout lines were weaker than in WT and *GNAT2* overexpression lines (previously reported OX6 with fivefold *GNAT2* upregulation; Wang et al. 2020a, b) (Fig. 1A). Furthermore, the quantitation of qS also showed that state transitions were significantly different among these genotypes, indicating that the mutants are unable to perform normal state transitions (Fig. 1B).

The low-temperature fluorescence emission spectra (77 K) of WT and the transgenic seedlings were measured to determine the changes in fluorescence. Thylakoids were extracted from growth light (GL, 200 µmol photons m^−2^ s^−1^) adapted plants or far-red light (FR, 735 nm) adapted plants (Koskela et al. 2018). In WT and OX6, the PSI peak (735 nm) was notably decreased under FR light treatment (state 1) compared with GL treatment (state 2) (Fig. 1C), confirming the redistribution of the light excitation energy between PSII and PSI through state transitions. In contrast, knockout lines showed the nearly same fluorescence emission peak at 735 nm under the two light regimes (Fig. 1C). Indeed, the ratio of 735 nm to 685 nm increased significantly in WT and OX6 under state 2 conditions while this was not the case in *gnat2 - 1* and *gnat2 - 2* (Fig. 1D). Furthermore, it is widely assumed that state transitions affect the dynamics of thylakoid membrane organization, in particular thylakoids in state 1 have more stacked grana membranes (Dietzel et al. 2011). Examination of the structure of thylakoid grana by transmission electron microscopy (TEM) indeed revealed that the stacking of thylakoid grana was slightly more pronounced in WT and OX6 than in the *gnat2 - 1* and *gnat2 - 2* mutants (Fig. S3). These results suggest that plants lacking GNAT2 protein will stay in state 1, similar to the *stn7* and *nsi Arabidopsis* mutants (Bellafiore et al. 2005; Koskela et al. 2018).

Due to the defect of state transitions in *GNAT2* knockout plants, we examined the photosynthetic complexes of thylakoid membranes in different lines using Blue-native (BN)-PAGE. The PSI-LHCI-LHCII supercomplex band, which represents the characteristic state transition complex, could be detected in WT and OX6, but not in *gnat2 - 1* and it was very faint in *gnat2 - 2* under growth light. Additionally, there was a slight increase of PSI complex and LHCII trimer (Fig. 1E). In contrast, no substantial differences were detected among all lines under far-red light. These results clearly show that LHCII in *GNAT2* knockout plants cannot form a complex with PSI under state 2 conditions, consistent with the previous observations with the *nsi* and *stn7 Arabidopsis* mutants (Suorsa et al. 2015; Koskela et al. 2018).

The general model of state transitions assumes that the STN7-dependent phosphorylation of LHCII is required for the assembly of PSI-LHCI-LHCII supercomplex (Pietrzykowska et al. 2014). To test whether the kinase activity was affected in *GNAT2* knockout lines, thylakoid membrane proteins were separated by sodium dodecyl sulphate (SDS)-PAGE and immunoblotted against a phospho-threonine antibody. Interestingly, the phosphorylation levels of LHCII were increased in the knockout lines compared to WT and the overexpression lines under state 2 conditions (Fig. 1F-G). These results indicate that the deficiency of state transitions in *GNAT2* knockout plants is not due to a decrease of phosphorylation. Possibly, acetylation and phosphorylation might act collectively in state transitions.

### GNAT2 affects agronomic traits of tomato under fluctuating light

**Fig. 2:**
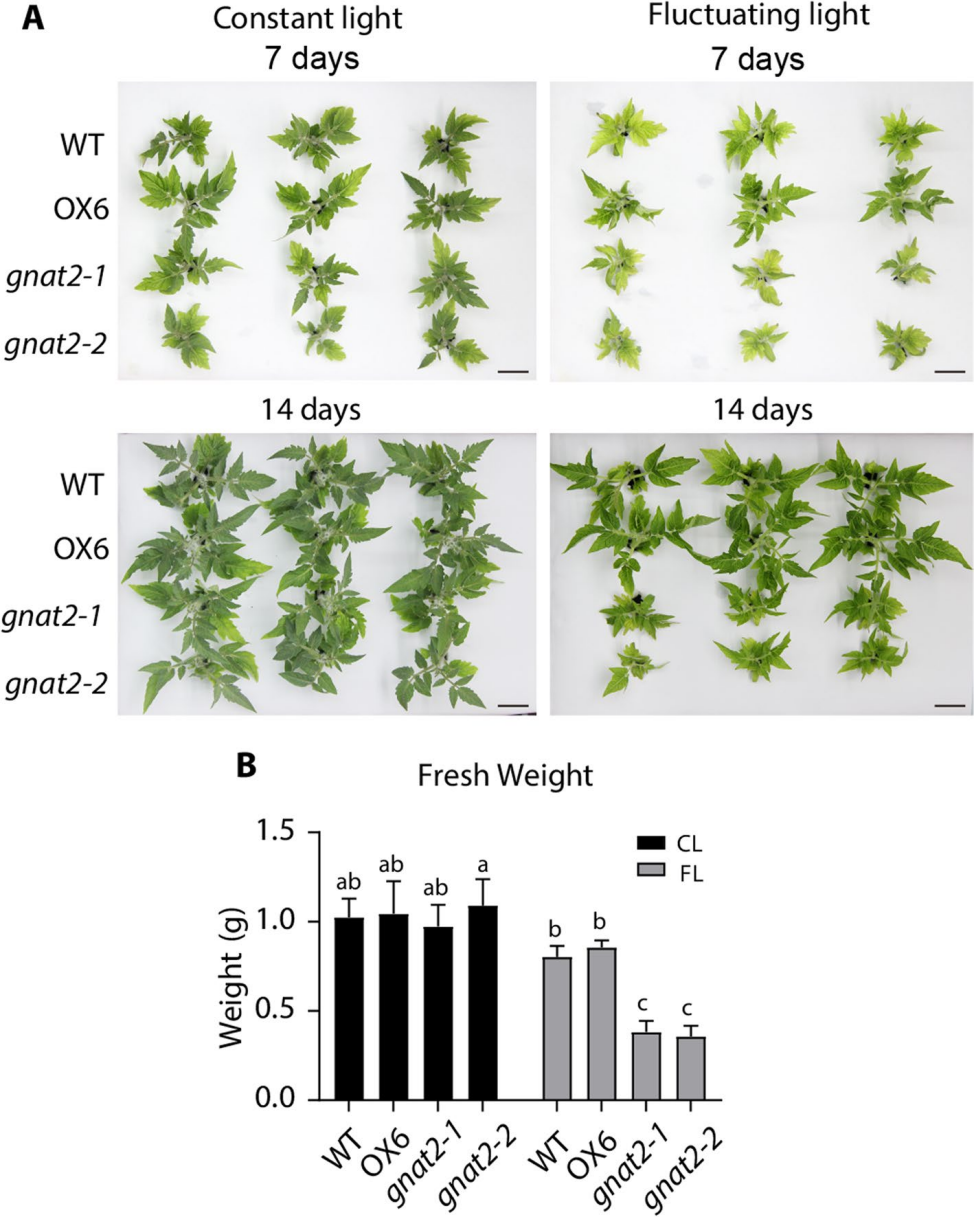
Growth of *GNAT2* knockout lines is impaired under fluctuating light. Phenotype of different lines grown under constant light (CL) and fluctuating light (FL). **A** For fluctuating light treatment, 3-week-old tomato seedlings were alternatively illuminated for 30 min at 200 μmol m^−2^ s^−1^ growth light and 30 min with far-red light (735 nm). For constant light treatment, tomato plants were illuminated with 200 μmol m^−2^ s^−1^ growth light. Phenotypes were observed after 7- and 14-days treatments, respectively. Scale bars, 2 cm. **B** Plant fresh weights were determined after 14 days treatments of different tomato lines. Values are means ± SD (*n* = 3 to 7). Different letters indicate significant differences (*P* < 0.05), as determined by two-way ANOVA using Tukey’s multiple comparisons test. *P* values were shown in Dataset 2

Growth of the *stn7* mutant is impaired under fluctuating light conditions, indicating that state transitions may play an important role in response to a changing light environment (Bellafiore et al. 2005; Tikkanen et al. 2010). To study whether the growth of *GNAT2* knockout lines was affected in fluctuating light, 2-week-old WT, OX6, *gnat2 - 1*, and *gnat2 - 2* plants were treated with fluctuating light (cycles of 30 min 200 µmol photons m^−2^ s^−1^ followed by 30 min of far-red light) for 2 weeks. As shown in Fig. 2A, growth of the *gnat2 - 1* and *gnat2 - 2* plants was slower compared to WT and OX6 lines after 7-days, and the differences became larger after 14-days, consistent with the change of fresh weight in all lines (Fig. 2B)(Koskela et al. 2020; Lee and Back 2021). Although the values of Fv/Fm were normal in the *GNAT2* knockout lines (Fig. S4A), the chlorophyll fluorescence parameter 1-qP was significantly higher under low light and especially after fluctuating light treatment (Fig. S4B-C), which reflects a more reduced PQ pool due to the impairment of state transitions in these lines. Furthermore, the chlorophyll (Chl) content (Chl *a* + *b*) and chlorophyll *a/b* ratio (Chl *a*/*b*) of the *GNAT2* knockout lines decreased more than in WT and OX6 after fluctuating light treatment (Fig. S4D-E).

To further explore the influence of GNAT2 on agronomic traits of tomato in a natural environment, *GNAT2* transgenic lines and WT were planted in a sunlight greenhouse without extra lighting facilities, and their phenotypes were examined for two consecutive growth seasons (Fig. S5A-C). Besides the determinate (set all fruit at once and suitable for one-time harvest) tomato cultivar Micro Tom, we also generated *GNAT2* overexpression and knockout lines in the indeterminate (set fruit until frost and provide prolonged harvest) tomato cultivar Ailsa Craig (AC) to obtain a more reliable phenotype of all tomato varieties (Fig. S5D-F). As shown in Fig. S5B-C and G-H, the ripening time of the overexpression lines in two growing seasons were not significantly different compared with WT, while the knockout lines had a significantly longer ripening time (about 5–10 days) than WT. Furthermore, expression analysis in tissue and organs showed that GNAT2 was mainly expressed in leaves (Fig. S5I). By continuously monitoring fruit development, we found that fruits of *gnat2* mutants had a slower growth rate and a lower fruit weight compared with WT before breaker stage (BR), when tomatoes reach physiologically maturity. After the BR stage, the fruit size and weight showed no substantial difference between these lines (Fig. S5J-K). Based on the above results, we propose that GNAT2 affects the fruit ripening process by influencing the accumulation of photosynthetic assimilates in plants. These results show that GNAT2 affects tomato fruit growth and ripening, a main agronomic trait of tomato.

### Essential role of ^131^Cys of GNAT2 for its involvement in state transitions

Analysis of the transient expression of GNAT2-GFP in tomato leaf protoplasts and immunolocalization analysis of chloroplast fractions indicated that GNAT2 is localized in the soluble fraction (Fig. S6 A-B). Since GNAT2 plays an important role in state transitions, a process governed by redox poise of the electron transfer chain (Rochaix 2013), we tested whether the function of GNAT2 is redox-regulated. First, due to the incomplete annotation of tomato data in the co-expression database, we analyzed the gene co-expression profiles of *NSI* from the ATTED-II database and found that several genes related to state transitions including *STN7*, *STN8* encoding the kinase of the PSII core proteins, *PPH1/TAP38* encoding the LHCII phosphatase and *PCBP* encoding the PSII core phosphatase are co-expressed with *NSI* based on the Pearson correlation coefficients (PCC). Interestingly, among these proteins, we found that some thioredoxin related-genes involved in chloroplast redox regulation, such as *LTO1*, *HCF164*, *Trx-f1* and *Trx-m4*, also have high correlation scores with *NSI* (Fig. S7), raising the possibility that *NSI* may be redox regulated.

**Fig. 3:**
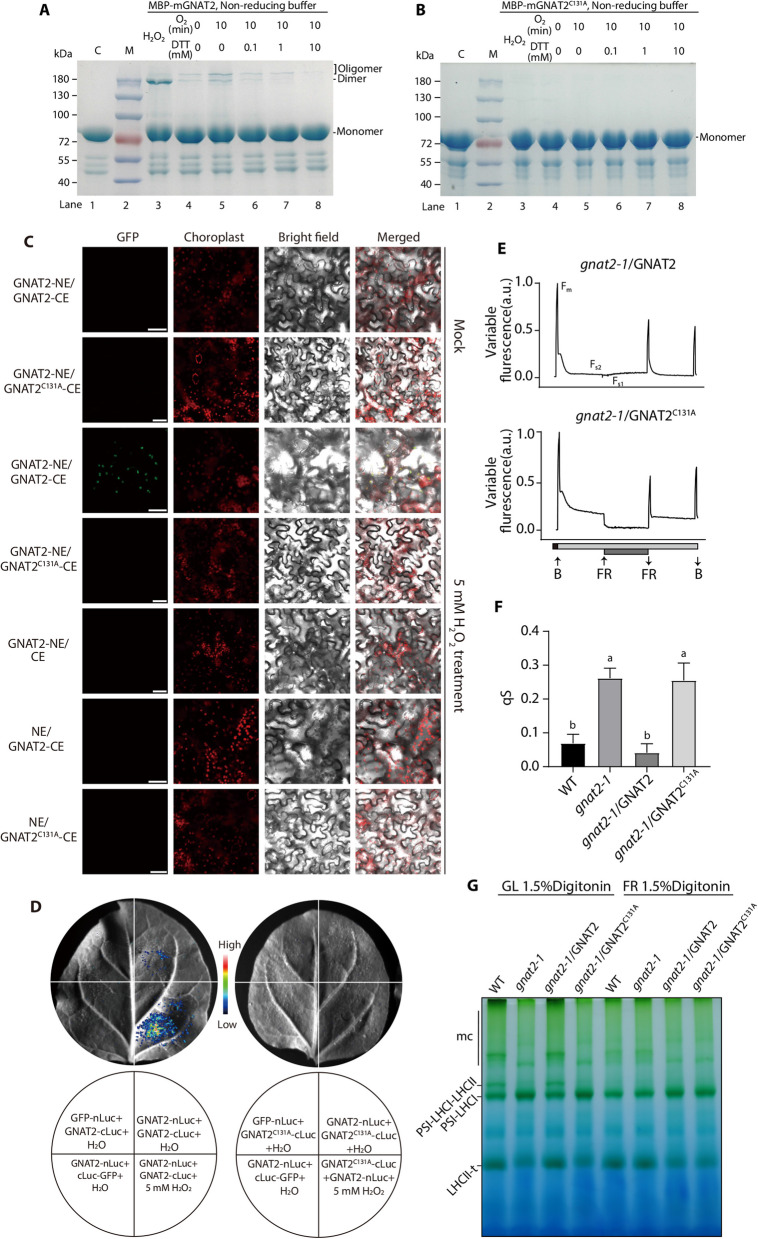
The conserved cysteine of GNAT2 is essential for state transitions by influencing its homodimerization. **A** DTT-dependent reduction of the cysteine in mGNAT2. Lane 1, mGNAT2 protein in reducing buffer as the control. Lane 2, marker. Lane 3, mGNAT2 incubated with 5 mM H_2_O_2_. Lane 4–8, O_2_-oxidized mGNAT2 was reduced with increasing concentrations of DTT for 90 min on ice. **B** mGNAT2^C131A^ protein cannot be redox-regulated. Lane 1, mGNAT2^C131A^ protein in reducing buffer as the control. Lane 3, mGNAT2^C131 A^ protein incubated with 5 mM H_2_O_2_. Lane 4–8, O_2_-oxidized mGNAT2^C131A^ was reduced with increasing concentrations of DTT for 90 min. **C** Interaction between GNAT2 homologous proteins confirmed by BiFC assay. Full-length GNAT2 protein was fused to the N terminus of EGFP (GNAT2-NE) and the C terminus of EGFP (GNAT2-CE), respectively. Fluorescence was observed after 3 h treatment with 5 mM H_2_O_2_. The mock group was treated with sterile water. Scale bars correspond to 40 µm. **D** Split-Luc assay for GNAT2 homologous proteins in tobacco. Full-length GNAT2 protein was fused to the N terminus of firefly Luc (GNAT2-nLuc) and the C terminus of firefly Luc (GNAT2-cLuc), respectively. And GFP protein was fused to nLuc or cLuc as negative control. Then, 5 mM H_2_O_2_ treatment was performed 3 h before fluorescence observation and sterile water was used as mock. **E** State transitions were measured from *gnat2 - 1*/GNAT2 and *gnat2 - 1*/GNAT2^C131A^. a.u., arbitrary units. **F** The value of qS. Values are means ± SD (*n* ≥ 5). Different letters indicate significant differences by one-way ANOVA using Tukey’s multiple comparisons test. **G** BN-PAGE of thylakoid complexes from WT, *gnat2 - 1*, *gnat2 - 1*/GNAT2 and *gnat2 - 1*/GNAT2^C131A^. mc, megacomplex; t, trimer

As cysteines play an essential role in the redox-regulated dimerization of STN7 (Wu et al. 2021), we further analyzed the sequence alignments of GNAT2 proteins from multiple species and found that only one cysteine (^131^Cys) in *GNAT2* is highly conserved (Fig. S8), which is localized on the outer side of the GNAT2 catalytic domain (Shirmast et al. 2021) (Fig. S9). We therefore checked whether the redox state influences the dimerization of GNAT2. The mature protein of GNAT2 was fused with the MBP tag which does not contain any cysteine residues and was used for the redox reactions. As shown in Fig. 3A, the mGNAT2 (mature GNAT2 protein with the transit peptide removed) can form a dimer and/or oligomer when oxidized under air or O_2_. The level of dimer was notably increased after treatment with the strong oxidant H_2_O_2_. When this protein was incubated with increasing concentrations of DTT, the dimer and oligomer levels of mGNAT2 were gradually decreased (Fig. 3A). Then, we mutated the highly conserved ^131^Cys of GNAT2 to alanine (C131 A) and found that it could no longer form a dimer or oligomer (Fig. 3B). To investigate whether ^131^Cys affects the formation of GNAT2 homomeric interactions in planta, we performed bimolecular fluorescence complementation (BiFC) and split-luciferase (Luc) assays in the leaves of *Nicotiana benthamiana* plants. The results showed that GNAT2 could form homomeric interactions under oxidative conditions in vivo, and this interaction was dependent on the ^131^Cys residue (Fig. 3C-D), which suggests that ^131^Cys of GNAT2 is critical for the redox-mediated formation of dimer/oligomer.

To further explore whether ^131^Cys-GNAT2 takes part in state transitions in vivo, we complemented the *gnat2 - 1* line with GNAT2 and GNAT2^C131A^ (a cysteine variant of GNAT2, Fig. S10 A-B). The Fv/Fm ratio was similar in WT, *gnat2 - 1*, *gnat2 - 1/*GNAT2 and *gnat2 - 1*/GNAT2^C131A^ (Fig. S10E). Then state transitions were examined by measuring the chlorophyll fluorescence. Interestingly, *gnat2 - 1*/GNAT2^C131A^ was similarly impaired in state transitions as *gnat2 - 1* plants while *gnat2 - 1*/GNAT2 had a similar state transition pattern as WT (Fig. 3E-F). Furthermore, the 77 K fluorescence emission spectra of *gnat2- 1*/GNAT2^C131A^ under growth light was reduced compared to WT and *gnat2 - 1*/GNAT2 (Fig. S11 A-C). And 1-qP value was significantly higher under low light in *gnat2 - 1*/GNAT2^C131A^ line (Fig. S11D). Moreover, BN-PAGE analysis showed that there was no PSI-LHCI-LHCII supercomplex in *gnat2 - 1*/GNAT2^C131 A^ plants, which was consistent with *gnat2 - 1* line, but *gnat2 - 1*/GNAT2 could form state transitions supercomplex in state 2 like WT (Fig. 3G), indicating that ^131^Cys plays a critical role in assembly of PSI-LHCI-LHCII supercomplex during state transitions. In addition, the phosphorylated levels of LHCII also were dramatically increased in *gnat2 - 1*/GNAT2^C131 A^, confirming that ^131^Cys residue is one of the important sites involved in the balance between acetylation and phosphorylation (Fig. S11E). Based on these results, we speculate that ^131^Cys is involved in the redox-mediated dimerization of GNAT2 to participate in state transitions.

### GNAT2 appears to be involved in the acetylation of.^6^Lys-Lhcb2

Based on the bio-informatics prediction, GNAT2 has a GCN5-related N-acetyltransferase domain, which is involved in lysine acetylation. Immunoblotting analysis showed that the acetylated protein levels decreased about 20% in *gnat2 - 2* and 40% in the *gnat2 - 1* lines, but increased about 10% in the *GNAT2* overexpression line compared with WT (Fig. S12), which suggests that GNAT2 is a lysine acetyltransferase.

Since GNAT2 is also involved in the synthesis of melatonin as a serotonin acetyltransferase (Back et al. 2016; Wang et al. 2020a), it was important to check whether melatonin plays a role in state transitions. Melatonin has been considered as a new plant hormone in recent years as its receptor CAND2/PMTR1 in plants was first reported in 2018, and low concentration of exogenous melatonin could induce stomatal closure (Wei et al. 2018). To assess the effectiveness of exogenous melatonin treatment, we investigated the stomatal aperture of different lines. The application of 10 μM melatonin significantly induced the stomatal closure in *Arabidopsis* plants Col- 0 and *nsi* while it had no effect in the melatonin receptor mutant *cand2 - 1*, indicating that melatonin treatment is working (Fig. S13 A). In a normal light environment, we found that the Fv/Fm ratio of Col- 0, *cand2 - 1, nsi,* and *nsi* treated with exogenous melatonin is not significantly different (Fig. S13B). In addition, exogenous melatonin could not alleviate the state transition-deficient phenotype of the *nsi* mutant (Fig. S13 C-D) suggesting that melatonin is not involved in state transitions. Thus, the impairment of state transitions of the *GNAT2* knockout lines results from the deficiency of GNAT2 acetyltransferase independent of its function in melatonin synthesis.

**Fig. 4:**
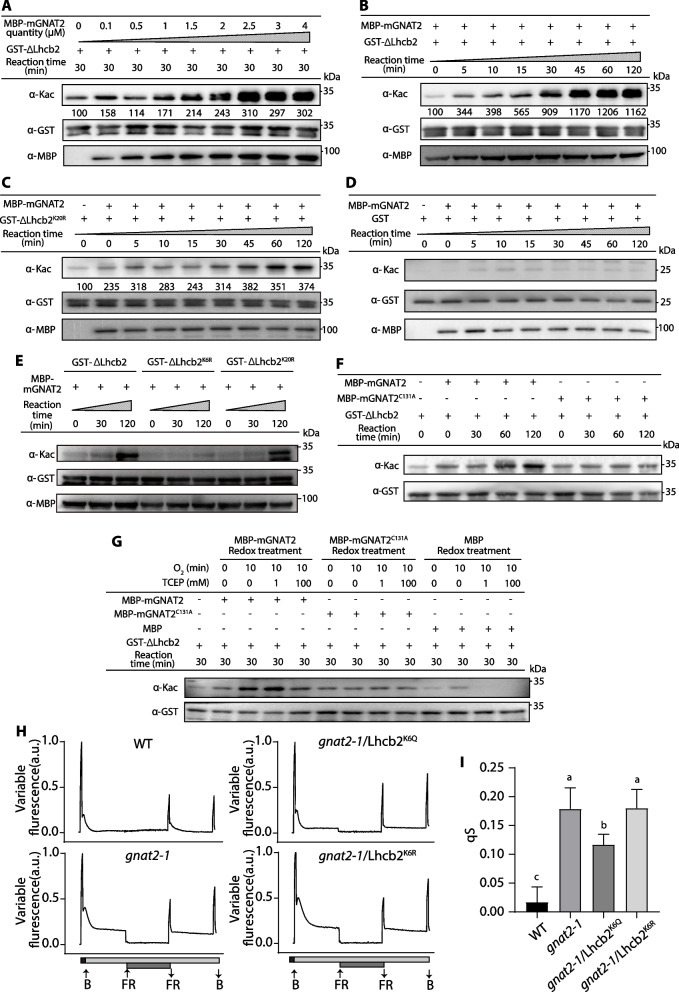
GNAT2 acetylates the N-terminus of Lhcb2 to affect state transitions. Acetylation of the N-terminus of Lhcb2 (ΔLhcb2) by the mature peptide (without chloroplast transit peptide) of GNAT2 (mGNAT2) is dependent on (**A**) mGNAT2 concentrations and (**B**) reaction time. The acetylated level of ΔLhcb2 was estimated by immunoblotting (Anti-acetyllysine antibody). The acetylated level of (**C**) ΔLhcb2^K20R^ and (**D**) GST protein by mGNAT2 at different reaction time. All the concentration of recombinant ΔLhcb2/ΔLhcb2^K20R^/GST as substrate was 1 µM. And in **B**-**D**, equal amount of recombinant mGNAT2 protein (1 µM) was incubated with substrate proteins. The abundance of ΔLhcb2/ΔLhcb2^K20R^/GST and mGNAT2 were detected with anti-GST and anti-MBP antibodies, respectively. “ + ” and “-” denote the presence or absence of the corresponding components in each reaction mixture, respectively. **E** The acetylation levels between ΔLhcb2, ΔLhcb2^K6R^ and ΔLhcb2^K20R^ protein. All the concentration of recombinant ΔLhcb2/ΔLhcb2^K6R^/ΔLhcb2^K20R^ as substrate was 1 µM. And equal amount of recombinant mGNAT2 protein (1 µM) was incubated with substrate proteins. **F** The conserved ^131^Cys of GNAT2 affects its catalytic activity for ΔLhcb2 in vitro. mGNAT2 or mGNAT2^C131A^ proteins (both 1 µM) were incubated with 1 µM GST-tagged ΔLhcb2 for increasing time at 30℃. Acetylated level of ΔLhcb2 was detected with Anti-acetyllysine antibody. **G** TCEP-dependent reduction affects the activity of mGNAT2 rather than mGNAT2^C131A^. mGNAT2/mGNAT2^C131A^ or MBP protein was firstly treated by O_2_ gas for 20 min, then reduced by 1 or 100 mM TCEP for 90 min on ice. After redox treatment, mGNAT2/mGNAT2^C131A^ /MBP proteins (both 1 µM) were incubated with 1 µM ΔLhcb2 for 30 min at 30℃, respectively. **H** State transitions were measured from WT, *gnat2 - 1*, *gnat2 - 1*/Lhcb2^K6Q^ and *gnat2 - 1*/Lhcb2.^K6R^. Tomato plants were dark-adapted for 30 min, then were exposed to blue light (B, 447 nm) for 15 min to induce state 2. Subsequently, FR light (735 nm) was turn on to induce state 1, and F_m1_ was measured after 15 min. Turn off the FR light and F_m2_ was measured after 15 min blue light treatment. a.u., arbitrary units. (I) The value of qS is inversely related to the extent of state transitions. Values are means ± SD (*n* ≥ 5). Different letters indicate significant differences (*P* < 0.05), as determined by one-way ANOVA using Tukey’s multiple comparisons test. *P* values were shown in Dataset 2

To explore the target proteins of the GNAT2 acetyltransferase, we screened by mass spectrometry (MS) the Lys acetylomes in the *GNAT2* knockout line *gnat2 - 1* and WT lines (Table. S1). Some of the identified acetylated proteins were found to be localized in chloroplasts and related to the light reactions of photosynthesis (Table 1, Dataset 3 A), including subunits of PSI and PSII. In particular, the acetylation of ^6^Lys site of Lhcb2 (SGN ID: Solyc07 g047850) had 18 peptide spectra in the Lys acetylome screen. Lhcb2 is a critical protein in state transitions because its phosphorylated ^4^Thr increases the affinity of the LHCII trimer to the PSI core in state 2 (Pan et al. 2018). The amino acid alignments of Lhcb2 isoforms from dicotyledons, monocotyledons, bryophytes, pteridophyte and algae reveal a highly conserved N-terminal region that contains ^4^Thr and ^6^Lys (Fig. S14). Furthermore, coincubation of the purified mGNAT2 (without signal peptide) and the N-terminal 50 amino acid domain of mature Lhcb2 protein (ΔLhcb2) in vitro showed that the acetylation level of ΔLhcb2 increased with the amount of added mGNAT2 (Fig. 4A) or when the reaction time was prolonged (Fig. 4B). The mass spectrometry analysis of ΔLhcb2 identified ^6^Lys as the acetylation site (Table. S2, Dataset 3B). To confirm this, we mutated the ^6^Lys and another Lys site (^20^Lys) to arginine (ΔLhcb2^K6R^ and ΔLhcb2^K20R^), which impaired the binding of the acetyl-lysine antibody. These experiments showed ΔLhcb2^K20R^ peptides had a weaker but the same acetylation pattern, while ΔLhcb2^K6R^ peptides were not acetylated by mGNAT2, and GST tag was used as control in the acetylation experiment (Fig. 4C-E).

**Table 1:** List of related acetylation sites of photosynthetic proteins

Protein name	Protein ID	Protein description	Subcellular localization	MS/MS Count	Position
CD4B	Solyc12g042060.1.1	ClpC; ATP-dependent Clp protease ATP-binding subunit ClpC	chloroplast	1	444
Lhcb1	Solyc03g005770.2	Chlorophyll a-b binding protein	chloroplast	2	290
Lhcb2	Solyc07g047850.3	Chlorophyll a-b binding protein	chloroplast	18	6
Lhcb4	Solyc09g014520.3	Chlorophyll a-b binding protein	chloroplast	4	34
NDP	Solyc06g005710.3	NAD(P)-binding domain-containing protein	chloroplast	2	460
				2	440
				1	356
				2	416
PsaN	Solyc08g013670.3.1	PsaN; photosystem I subunit PsaN	chloroplast	6	154
				22	132
PsbB	Solyc01g007500.2.1	Photosystem II CP47 reaction center protein	chloroplast	2	321
				4	304
PsbO	Solyc02g065400.3.1	PsbO; photosystem II oxygen-evolving enhancer protein 1	chloroplast	7	273
				8	157
PsbP	Solyc01g087040.2.1	PsbP domain-containing protein	chloroplast	1	115
				2	224
PsbQ	Solyc02g079950.3	psbQ; photosystem II oxygen-evolving enhancer protein 3	chloroplast	2	184

GNAT2 belongs to the GNAT family, and several structural studies revealed that many members of this family can form dimers or oligomers (Ud-Din et al. 2016; Shirmast et al. 2021). Another study reported that assembly into oligomeric complexes was necessary for NSI function in *Arabidopsis* (Carvalho et al. 2006). Thus, it was important to check whether the redox-mediated dimerization of GNAT2 affects its acetyltransferase activity and participates in state transitions. Accordingly, mGNAT2 and mGNAT2^C131A^ were used in parallel to test their activity on the substrate ΔLhcb2. In contrast to the wild-type protein, the degree of acetylation of ΔLhcb2 protein did not increase after co-incubation with mGNAT2^C131A^ (Fig. 4F), suggesting that ^131^Cys is involved in the regulation of the catalytic activity of GNAT2. Then we explored the effect of redox-related dimerization on the catalytic activity of GNAT2. The oxidized mGNAT2 had a higher catalytic activity for ΔLhcb2 compared to the reduced form, and the degree of acetylation of ΔLhcb2 protein did not increase after co-incubation with mGNAT2^C131A^ whether under reduced or oxidized conditions (Fig. 4G), indicating that the redox-mediated dimerization of GNAT2 is required for its acetyltransferase activity. In conclusion, these results suggest that the redox state controls the catalytic activity of lysine acetyltransferase in a ^131^Cys-dependent way.

Moreover, total protein of WT, OX6 and *gnat2* mutant plant was extracted, and incubated with the ΔLhcb2^K20R^ protein, to ensure that the detected acetylation modifications are all attributable to ^6^Lys rather than confounding effects from ^20^Lys. After immunoprecipitation, ΔLhcb2^K20R^ protein showed highest acetylation levels after incubation with OX6, and lower acetylation levels with mutants (Fig. S15 A). Further research shows that ^6^Lys could be acetylated in *gnat2 - 1*/GNAT2 mutants but not in *gnat2 - 1*/GNAT2^C131A^ mutants (Fig. S15B). These results indicate that ^6^Lys of Lhcb2 is a GNAT2 acetylation substrate in vitro and in vivo, and is associated with ^131^Cys of GNAT2.

Then we checked whether ^6^Lys-Lhcb2 plays a role during state transitions in vivo. The phenotype of the Lhcb2^K6Q^ (an acetylation-mimetic site mutant) and the Lhcb2^K6R^ (an acetylation-deficiency site mutant) transgenic line in *gnat2 - 1* background (*gnat2 - 1*/Lhcb2^K6Q^ and *gnat2 - 1*/Lhcb2^K6R^) showed that the Fv/Fm ratio was similar in WT and *gnat2 - 1* (Fig. S10 C-E). State transitions were examined by measuring F_S1_ and F_S2_ (Fig. 4H). As shown in Fig. 4I, the qS value of *gnat2 - 1*/Lhcb2^K6Q^ lines was significantly lower than for *gnat2 - 1* and *gnat2 - 1*/Lhcb2^K6R^ although it was still higher compared to WT, while the qS values of *gnat2 - 1*/Lhcb2^K6R^ was not different from *gnat2 - 1*, indicating that the continuous acetylation of ^6^Lys-Lhcb2 could partly restore state transitions of *gnat2 - 1*. Furthermore, the phosphorylated levels of LHCII were increased in the complemented lines compared with WT (Fig. S16), indicating that *gnat2 - 1*/Lhcb2^K6Q^ and *gnat2 - 1*/Lhcb2^K6R^ can still be phosphorylated. This result supports the proposal that acetylation of ^6^Lys-Lhcb2 favors state transitions.

## Discussion

Most research on light acclimation responses was performed with plants grown in a stable and controlled environment. However, plants must cope with dynamic and changing light conditions during the day in a natural environment. State transitions are a dynamic mechanism to balance the excitation energy of PSI and PSII within minutes (Vialet-Chabrand et al. 2017; Slattery et al. 2018). Studies of state transitions have been reported for cyanobacteria and algae, but their role has been less studied in higher plants except for the model plant *Arabidopsis*. Tomato was chosen for this study because it serves as both a model plant for molecular research and a major vegetable crop for Agricultural production. While the mechanism may be generalizable, testing it in tomato directly demonstrates its potential to improve photosynthetic efficiency in a crop where even small gains could yield significant economic and nutritional benefits.

NSI/SNAT1/GNAT2 is generally considered as a key enzyme in the melatonin synthesis pathway, and its role as a plastid lysine acetyltransferase in state transitions was only found recently (Koskela et al. 2018). Melatonin is an indoleamine synthesized mainly in chloroplasts and participates in the regulation of plant growth, biological rhythm, cell cycle and ROS scavenging (Tan et al. 2013). In particular, melatonin plays a protective role in the photosynthetic electron transport chain and D1 protein synthesis, enhances the tolerance to salt stress, and regulates the level of chloroplast proteins Trx-f and Trx-m, which affect the electron transport efficiency (Zhou et al. 2016). However, whether melatonin is involved in state transitions has not been discussed in previous work (Koskela et al. 2018). To explore the relationship between melatonin and state transitions, we checked the phenotypes of *cand2 - 1*, the first reported phytomelatonin receptor mutant of *Arabidopsis*, and found that state transitions of *cand2 - 1* were similar to those of WT plants. Furthermore, exogenous feeding of melatonin to *nsi* mutants did not restore state transitions which proved that loss of melatonin is not the direct cause of the impairment of state transitions in *nsi* mutants.

State transition, an important light acclimation process, restores the balance of the light excitation energy between the two photosystems and is also regulated by the chloroplast redox state under fluctuating light (Rochaix 2013). As a major regulatory protein of state transitions, the STN7 kinase is regulated by the redox state of the PQ pool and the FD/Trx system in different light environments (Bellafiore et al. 2005; Lemeille et al. 2009). Under normal light, LTO1 interacts with STN7 to maintain STN7 in an oxidized state through the lumenal Trx-like domain, while under high light, the Trx-m might act as a stromal electron donor for the CcdA/HCF164 trans-thylakoid redox pathway to reduce the disulfide bonds of STN7, thereby inactivating its kinase activity (Lennartz et al. 2001; Ancin et al. 2019; Wu et al. 2021). As a lysine acetyltransferase is required for state transitions, it was necessary to explore the redox-dependent regulatory mechanisms of GNAT2 in tomato. First, we found that there was substantial co-expression between *NSI* and a large number of redox-related genes (Fig. S7) which is similar to the co-expression profiles of STN7 (Ancin et al. 2019; Wu et al. 2021). Additionally, a previous report showed that the oligomeric complexes of NSI are important for acetyltransferase function in *Arabidopsis*. Examples of dimer/oligomer structure affecting enzyme activity have also been described for other members of GNAT family (Carvalho et al. 2006; Srivastava et al. 2014; Tsimbalyuk et al. 2020). Noticeably, inter-molecular disulfide bonds mediating the dimerization of STN7 are also essential for its kinase activity (Wu et al. 2021) indicating a similarity between STN7 and GNAT2 that are both involved in the redox regulation of state transitions. The latest crystal structure of OsGNAT2 in rice (*Oryza Sativa*) reveals that the unique cysteine (^128^Cys-OsGNAT2, corresponding to ^131^Cys-GNAT2) is located on the outer side of the OsGNAT2 dimer (Fig. S9). These results suggest that the conserved cysteine might affect the catalytic activity of GNAT2 through conformational changes to participate in state transitions. However, we could not identify the redox factor(s) specifically involved in regulating the redox state of GNAT2. Based on the results obtained in this study, we propose that the protein involved in regulating GNAT2 activity should be an oxidase with its redox regulated domain located on the stroma side of the thylakoid membrane so that it can interact with GNAT2.

Acetylation is one of the more conservative post-translational modifications of proteins in plants, and a large number of chloroplast proteins are acetylated (Wu et al. 2011). Lys acetylomes in the *gnat2 - 1* mutants identified some proteins localized in chloroplasts and related to the light reactions of photosynthesis. Some proteins partially overlap the set of proteins whose acetylation is affected in *nsi* although the modified sites are not completely consistent (Koskela et al. 2018). We verified that ^6^Lys of Lhcb2 is a target site and can be acetylated by ^131^Cys-dependent GNAT2 in vitro and in vivo (Fig.S15 A-B), and that acetylation of this site is required, at least to some extent, in state transitions in vivo (Fig. 4H-I). Since the complementation with Lhcb2^K6Q^ only partially restored the defect of state transitions in *GNAT2* knockout plants, we speculate that other chloroplast proteins might be acetylated by GNAT2, such as the oxygen-evolving complex (OEC) proteins (PsbO, PsbP and PsbQ) and PsaN (Table 1), which in turn take part in the assembly of PSI-LHCI-LHCII complex and thus participate in state transitions together.

**Fig. 5:**
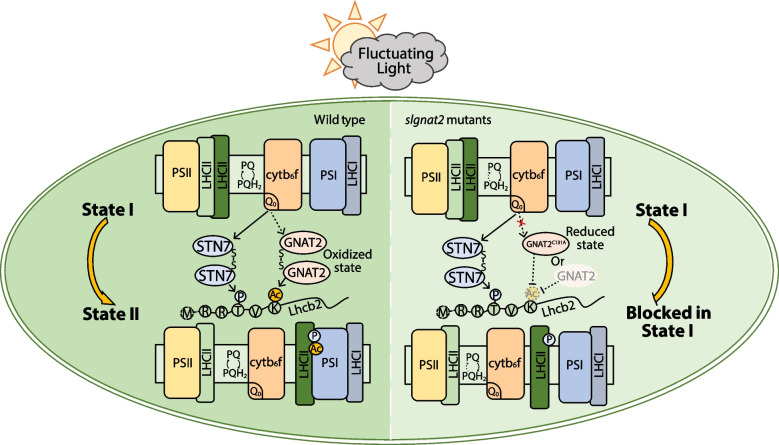
Working model of GNAT2 during state transitions in tomato. This model is modified from (Rochaix, 2011). Under fluctuating light, the redox status of the plastoquinone (PQ) pool regulates STN7 kinase activity, driving LHCII phosphorylation. Meanwhile, the dimerization of GNAT2 is also redox-regulated via the ^131^Cys residue, and has acetyltransferase activity in the oxidized state. Furthermore, ^6^Lys of Lhcb2 is acetylated by GNAT2 to enhance the binding of the LHCII trimer to PSI during the transition from state 1 to state 2. In *gnat2* knockout mutant or *gnat2 - 1*/GNAT2.^C131 A^ mutant, GNAT2 is unable to form disulfide bonds and thus in reduced or inactivated state. Although phosphorylation can occur, it still affects the formation of the PSI-LHCI-LHCII supercomplex, leaving the plant locked in state 1

Based on our results, we speculated the following working model. In wild-type plants, when PSII is over excited, PQ pool is reduced, then STN7 and GNAT2 are activated by the redox system to form homodimers which increases their enzyme activity of phosphorylation and acetylation on the Lhcb2 protein, respectively. The post-translational modifications of Lhcb2 and other candidate targets could change the structural conformation of photosynthetic proteins to promote the assembly of the PSI-LHCI-LHCII complex essential for the transition from state 1 to state 2. In *gnat2* mutants or *gnat2 - 1*/GNAT2 line, GNAT2 is unable to form disulfide bonds and thus in reduced or inactivated state. Lhcb2 cannot be completely acetylated while STN7 may be over-stimulated to increase the phosphorylation level of LHCII through a compensatory effect or an unknown feedback regulatory pathway. However, LHCII is still unable to bind to PSI due to the lack of acetylation and the plant is blocked in state 1 (Fig. 5). Although the relationship between lysine acetylation and state transitions was explored in this work, numerous questions will require further investigation in the future. For example, are there still other acetylation target sites of GNAT2 which participate in state transitions? Is Lhcb2 regulated by other chloroplast acetyltransferases? What are the enzymes that regulate the redox state of GNAT2 in chloroplasts?

## Materials and Methods

### Plant materials and fluctuating light treatments

Seeds of tomato cultivar Micro Tom obtained from Ball Horticultural Company (Lot 0020224203, U.S.A.), and seeds of tomato cultivar Ailsa Craig (AC) from our laboratory were used. Tomato seedlings were grown in a growth-chamber (Ningbo Jiangnan GXM- 1008) as follow: 12 h light/12 h darkness at PPFD of 200 µmol m^−2^ s^−1^ (light source: full spectrum light emitting diodes [LED] used were model LZJ021200360 [OPPLE, China], which emit wavelengths spanning 400–700 nm with peaks at 450 nm (blue) and 660 nm (red), simulating natural sunlight spectra), 60% humidity, and 24 °C light/18 °C darkness. The 2-week-old seedlings were transferred to polyethylene pots filled with 0.8 L Hoagland solution (Arnon and Hoagland 1939). The nutrient solution was refreshed every week and growth conditions were the same as described previously. For fluctuating light treatment, the 3-week-old tomato plants adapted for 1 week in Hoagland solution, were illuminated under fluctuating light cycles of 30 min under 200 µmol m^−2^ s^−1^ irradiance (light source: full spectrum LED and 660 nm red light) followed by 30 min of illumination with far-red light treatment (light source: 735 nm far-red light LED) in a growth-chamber.

### Plant transformation

To construct *GNAT2*-knockout mutant tomato plants, a potential guide sequence (Guide sequence: CAGTAGAAGTGGATACTAAG, PAM site: TGG) for the CRISPR/Cas9 system of *GNAT2* was predicted by the website CRISPOR (http://crispor.tefor.net/) (Concordet and Haeussler 2018). The *sg*RNA driven by *AtU6* promoter were cloned into the binary vector pBSE402 which confers resistance to Basta (Xing et al. 2014). Recombinant plasmids were transferred into *Agrobacterium tumefaciens* GV3101 and then transferred into Micro Tom and AC tomato using the transformation and regeneration methods (Van Eck et al. 2006). Prediction and detection of potential off-target sites caused by CRISPR/Cas9-editing of *GNAT2* are shown in Table. S3, and primers used for construction of CRISPR constructs and screening of transgenic plants for gene editing were listed in Table S4.

In addition, Micro Tom *GNAT2* overexpression line OX6 was maintained in our laboratory (Wang et al. 2020a), and *GNAT2* overexpression lines of AC were generated by the *pCAMBIA1305*-*35S*_*pro*_:*GNAT2*: Flag vector. The primers were listed in Table. S4.

### State transitions fluorescence measurements

A FluorCam800MF kinetic imaging fluorimeter (Photon Systems Instruments) was used for state transition fluorescence measurements. After at least 30 min dark adaptation, an intact leaf was fixed to the detection device. First, the maximum fluorescence yield (F_m_) was measured with a saturated light pulse (0.8 s, 3000 µmol m^−2^ s^−1^). Then, the leaf was illuminated for 15 min with blue LED (447 nm, 67 µmol m^−2^ s^−1^) to reach steady-state fluorescence F_S2_. Subsequently, a far-red light LED (735 nm) was switched on for 15 min to reach steady-state F_S1_. Then the maximal fluorescence yield in state 1 (F_m1_) was determined. The far-red light was turned off and the maximum fluorescence yield in state 2 (F_m2_) was measured after 15 min of blue light irradiation. The state transitions parameter qS were calculated according to the formula: qS = (F_S2_-F_S1_)/F_S2_ (Wu et al. 2021), the value of qS was inversely related to the extent of state transitions. Measurements for each tomato line were performed with at least three biological replicates on the fifth leaf of 6-week-old tomato plants.

### Plant protein extraction and immunoblot analysis

Six-week-old tomato leaves were ground in liquid nitrogen into powder and then transferred to a new centrifuge tube. Total protein was extracted using a protein extraction buffer (50 mM HEPES, pH 7.5, 150 mM KCl, 1 mM Ethylene Diamine Tetraacetic Acid [EDTA], 0.5% Triton X- 100, 1 mM DTT and 1 × protease inhibitor cocktail [CWBIO, Beijing]). The samples were centrifuged at 12,000 g at 4 °C for 20 min. The upper liquid phase was transferred to a new centrifuge tube and the protein concentration was determined with the BCA kit according to the manufacturer’s instruction (Coolaber, Beijing, Lot. SK1070).

More details for immunoblotting and all original images of the western bolt membranes and Coomassie-Brilliant Blue (CBB) staining are provided in Dataset 1.

### Thylakoid membrane extraction

Thylakoid membranes were extracted from fresh tomato leaves or protoplasts after transient transformation in cold extraction buffer (400 mM sucrose, 10 mM NaCl, 2 mM MgCl_2_, 50 mM HEPES–KOH, pH 7.8 and 10 mM NaF) (Wu et al. 2021). The homogenate was filtered through Miracloth (Millipore), and centrifuged for 4 min, 5000 g, 4 °C. The pellet composed of chloroplasts and thylakoids was resuspended in hypotonic lysis buffer (5 mM sucrose, 10 mM HEPES–KOH, pH 7.6, 5 mM MgCl_2_) to break the chloroplasts. The lysate was centrifuged at 18,000 g for 5 min, 4 °C, the supernatant was collected to analyze the soluble protein fraction of chloroplasts, and the pellet which contained thylakoid membrane proteins was resuspended in storage buffer (100 mM sucrose, 10 mM HEPES–KOH, pH 7.6, 10 mM MgCl_2_ and 10 mM NaF) (Koskela et al. 2018).

### 77 K fluorescence emission spectra

Chlorophyll fluorescence emission spectra of thylakoid membrane suspensions were measured at 77 K (liquid nitrogen). Thylakoids were diluted with storage buffer (100 mM sucrose, 10 mM HEPES–KOH, pH 7.6, 10 mM MgCl_2_ and 10 mM NaF) to 5 μg/mL chlorophyll concentration. Chlorophyll fluorescence emission spectra were recorded with a Hitachi F- 7000 fluorescence spectrophotometer (Serial number: 2137–002; ROM version: 5 J14000 06). Measurement type was wavelength scan, the scan mode and data mode were emission and fluorescence, respectively. Excitation wavelength was 435 nm, increment 0.2 nm and scan speed was 240 nm/min. The spectra were normalized at 685 nm, corresponding to the peak of PSII fluorescence, and smoothed by GraphPad Prism 8.0 software.

### Detection of thylakoid membrane protein complexes by Blue-native (BN)-PAGE

Purified thylakoid membranes were quantified by chlorophyll concentration, and equal amounts of thylakoid membranes (300 µg chlorophyll) were diluted to a final concentration of 1 µg chlorophyll/µL buffer (25 mM Tris–HCl, 0.2% [*v/v*] glycerol, 10 mM NaF and 1 × protease inhibitor cocktail). Then the thylakoids were solubilized *with* 1.5% (*w/v*) digitonin (Sigma) for 15 min at 4 °C. The supernatant was collected after centrifugation at 12,000 g at 4 °C for 10 min. Serva Blue G Buffer (100 mM Tris–HCl, pH 7.0, 0.5 M 6-amino-*n*-caproic acid, 50 mg/mL Serva Blue G, 30% [*w/v*] sucrose) was used to stain thylakoids. The samples were applied to 5–12% acrylamide gradient gels with a gradual increase in voltage during electrophoresis at 4 °C.

### Lys acetylome screening

For preparation of Lys acetylome, 6-week-old WT and *gnat2 - 1* seedlings were used for screening. Frozen leaf material was ground to powder in liquid nitrogen then transferred to a 5-mL centrifuge tube. After that, four volumes of lysis buffer (2 mM EDTA, 1% Triton X- 100, 10 mM dithiothreitol, 3 μM Trichostatin A, 50 mM Nicotinamide [NAM] and 1% Protease Inhibitor Cocktail VI [Calbiochem], pH 8.0) was added to the cell powder, followed by sonication three times on ice using a high intensity ultrasonic processor (Scientz). Then an equal volume of tris-saturated phenol was added and the mixture was vortexed for 5 min. The remaining sediment was removed by centrifugation at 5,500 g at 4 °C for 10 min. The protein was precipitated with at least four-volume of icy ammonium sulfate-saturated methanol for 6 h at − 20 °C. The supernatant was discarded after centrifugation at 12,000 g at 4 °C for 10 min. The remaining precipitate was washed with cold methanol, followed by cold acetone for three times. Then the protein was redissolved in 8 M urea and the concentration was determined with BCA kit (Beyotime, P0010) according to the manufacturer’s instructions.

After adjusting the volume of the same amount of protein with the lysate, the final concentration of 20% TCA was added and precipitated overnight at 4 °C. After centrifugation at 5,500 g 4 °C for 10 min, the supernatant was discarded. Then washed and precipitated with pre-cooled acetone three times, the protein precipitate was dried, a certain volume of 200 mM Triethyl Ammonium bicarbonate (TEAB) was added, followed by ultrasonic dispersion precipitation. Trypsin was added at 1:50 trypsin-to-protein mass ratio for the first digestion overnight and 1:100 trypsin-to-protein mass ratio for a second 4 h-digestion. After trypsin digestion, peptides were desalted by Strata X C18 SPE column (Phenomenex) and vacuum-dried. Peptide was reconstituted in 0.5 M TEAB and processed according to the manufacturer’s protocol for TMT 6 PLEX kit (Thermo Fisher Scientific Inc, Rockford, IL, USA, Batch: Lot#WE330299) according to the manufacturer’s instructions. Then the tryptic peptides were fractionated by high pH reverse-phase HPLC using Thermo Betasil C18 column (5 μm particles, 10 mm ID, 250 mm length). The peptide fragments were then co-incubated with anti-acetyllysine antibody conjugated agarose beads (PTM BioLab, Inc., Hangzhou, China, Lot. PTM- 104) overnight to enrich the modified peptides.

### Protein expression and purification

The nucleotide sequence encoding the mature peptide (without chloroplast transit peptide) of GNAT2 (mGNAT2 protein) was amplified with mGNAT2-S/A primers and the nucleotide sequence encoding the first 50 amino acid of the N-terminal SlLhcb2 mature peptide (ΔLhcb2 protein) was amplified with ΔSlLhcb2-S/A primers. For the site-directed mutagenesis assay, the ^131^Cys of GNAT2 protein was replaced with Ala and the ^20^Lys of Lhcb2 protein was replaced with Arg using QuickMutation™ Plus Site-Directed Mutagenesis Kit (Beyotime, D0208). Related primers are listed in Table. S4. The PCR fragments were subcloned into the pMAL-C2 vector in the *Bam*HI and *Eco*RI sites (mGNAT2 proteins), and pGEX- 4 T- 1 vector in the *Bam*HI site (ΔLhcb2 proteins). To induce the proteins, vectors were transformed into *E. coli* strain Rosetta (DE3) and induced with 1 mM isopropyl-beta-D-thiogalactopyranoside (IPTG) for overnight at 16 °C. Cells were harvested by centrifugation at 5,000 g for 10 min at 4 °C, and the pellets of recombinant proteins were resuspended in buffer (20 mM Tris–HCl, 200 mM NaCl, 1 × protease inhibitor cocktail, pH 7.8), then cells were disrupted by Ultrasonicator. The recombinant mGNAT2 proteins with maltose-binding protein (MBP) tag were purified by MBP Sepharose (NEB, E8021 V) and recombinant SlLhcb2 proteins with Glutathione S-transferase (GST) tag were purified by Glutathione Sepharose™ 4B (GE Healthcare, 17–0756 - 01) as manual.

### Assay of acetyltransferase activity

For acetylation, in general, each reaction contained equal molarity (1 µM) of purified recombinant MBP-mGNAT2 protein (3.5 µg in 50 µL buffer) as catalyst, and purified recombinant GST-ΔLhcb2/GST-ΔLhcb2^K20R^ protein (1.6 µg in 50 µl buffer) or purified GST protein (1.3 µg in 50 µL buffer) as substrate in assay buffer containing 50 mM Tris–HCl (pH 8.0), 1 mM acetyl-CoA (Roche), 50 mM NaCl, 5 mM MgCl_2_, 2 mM NAM, 1 mM sodium butyrate, and 5% glycerol, as described by Gao (Gao et al. 2016). All reactions were performed at 30 °C for different times as described. After incubation, proteins were fractionated by 12% SDS-PAGE with 2 × reducing loading buffer (4% [*w/v*] SDS, 100 mM Tris–HCl, pH 6.8, 20% [*v/v*] glycerol, 0.2% [*w/v*] bromophnol blue and 1% [*w/v*] 2-Mercaptoethanol) and visualized by CBB staining (20 µL per lane). The anti-acetyllysine antibody (PTM Biolabs, Hangzhou, Lot. PTM- 101) was used to determine the acetylation level of substrate protein.

For the semi-vitro experiment to verify acetylation level, ΔLhcb2^K20R^ protein was coupled to Glutathione Sepharose™ 4B. Then the ΔLhcb2^K20R^ agarose (40 µL/sample) was incubated with total protein (0.2 mg/sample) of different lines for 30 min at 30 °C. After incubation, the ΔLhcb2^K20R^ agarose was washed 7 times with 1 × PBS buffer and then eluted with 10 mM reduced glutathione. Acetylation level was detected by acetyllysine antibody.

### In *vitro* redox assays

In vitro redox assays were performed according to Wu (Wu et al. 2021). Briefly, recombinant MBP-mGNAT2 was incubated with 200 mM DTT for 1 h on ice to ensure its reduced state, and then the protein was treated with oxygen gas to obtain the weak oxidation state or incubated with 0.15% H_2_O_2_ for 1 h on ice to obtain maximal oxidation. Incubation with DTT at different concentration for 1 h was used to assess the change from dimer to monomer (reduced state). Proteins were fractionated by 12% SDS-PAGE with 2 × nonreducing loading buffer (4% [*w/v*] SDS, 100 mM Tris–HCl pH 6.8, 20% [*v/v*] glycerol, 0.2% [*w/v*] bromophenol blue) and visualized by CBB staining.

For in vitro redox assay of acetyltransferase activity, purified recombinant MBP-mGNAT2, MBP-mGNAT2^C131 A^ and MBP proteins were oxidized by oxygen gas for 20 min, then treated by different concentrations of Tris(2-carboxyethyl) phosphine (TCEP) for 90 min to induce the reduced state. The whole process took place on ice. All tubes and solutions were treated with nitrogen gas to protect the proteins from oxidation by air. For acetylation, each reaction contained equal molarity (1 µM) of redox-treatment MBP-mGNAT2 or MBP-mGNAT2^C131 A^ or MBP protein as catalyst, and 1 µM GST-ΔLhcb2 protein as substrate in assay buffer containing 50 mM Tris–HCl (pH 8.0), 1 mM acetyl-CoA (Roche), 50 mM NaCl, 5 mM MgCl_2_, 2 mM NAM, 1 mM sodium butyrate, and 5% glycerol. All reactions were performed at 30 °C for 30 min. After incubation, proteins were fractionated by 12% SDS-PAGE with 2 × reducing loading buffer. The anti-acetyllysine antibody (PTM Biolabs, Hangzhou, Lot. PTM- 101, dilution 1:5000) was used to determine the acetylation level of substrate protein.

### Statistical analyses

GraphPad Prism 8.0 and Microsoft Office Excel were used for statistical analysis of the numerical data. Significant differences between two groups were analyzed by using the two-tailed Student's *t*-test. * *P* < 0.05 was considered to indicate statistical significance, ** *P* < 0.01 was considered highly significant, *** *P* < 0.001 was considered extremely significant and ns indicates not significant. Significant differences between multiple samples were determined by one-way or two-way analysis of variance (ANOVA) followed by Tukey's multiple range test with GraphPad Prism 8.0 software and different letters are used to indicate significant differences. Statistical data are provided in Dataset 2.

## Supplementary Information

Supplementary Material 1.

Supplementary Material 2.

Supplementary Material 3.

Supplementary Material 4.

Supplementary Material 5.

## Data Availability

The mass spectrometry proteomics data have been deposited to the ProteomeXchange Consortium (http://proteomecentral.proteomexchange.org) via the iProX partner repository with the dataset identifier PXD040401 and PXD054007. All data are available in the main text or the supplementary materials.

## Abbreviations

CEF: Cyclic electron flow
Cys: Cysteine
ETR: Electron transport rate
GNAT: GCN5-related N-acetyltransferase
LEF: Linear electron flow
LHCI: Light harvesting complex I
LHCII: Light harvesting complex II
Lys: Lysine
NPQ: Non-photochemical quenching of fluorescence
NSI: Nuclear shuttle interacting
OEC: Oxygen-evolving complex
P700: Primary electron donor to PSI
PC: Plastocyanin
PQ: Plastoquinone
PSI: Photosystem I
PSII: Photosystem II
SNAT: Serotonin N-acetyltransferase
STN7: Serine/Threonine-protein kinase 7
*Sl*: *Solanum lycopersicum* L.
WT: Wild type
