# Supplementary material for: Chloroplast acetyltransferase GNAT2 acts as a redox-regulated switch for state transitions in tomato
Source: Mol Hortic. 2025 Aug 6;5:39. doi: 10.1186/s43897-025-00164-0 (PMC12326663; doi:10.1186/s43897-025-00164-0)
Supplement: Supplementary file 1 — Supplementary Material 1. [file 43897_2025_164_MOESM1_ESM.pdf]

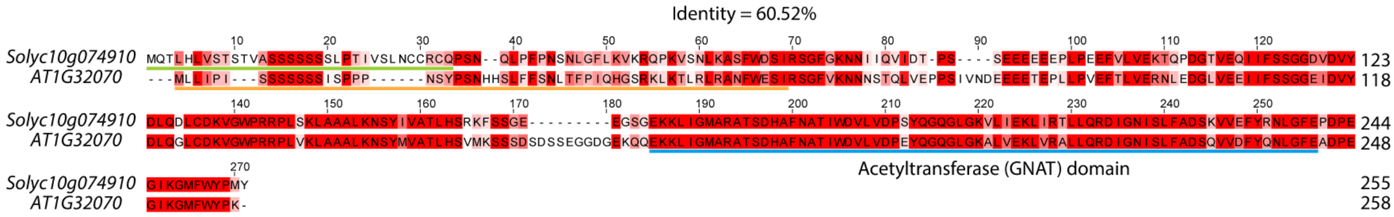

Supplemental Figure 1. Amino acid sequence alignment of SlGNAT2 and NSI.

Amino acid sequence alignment of SlGNAT2 (Solyc10g074910) and NSI (AT1G32070) was performed with Clustal Omega (https://www.ebi.ac.uk/Tools/msa/clustalo/) and displayed with Jalview. GNAT domain was predicted with the NCBI-CDD website (https://www.ncbi.nlm.nih.gov/Structure/cdd/wrpsb.cgi) and is underlined with blue. The transit peptides of SlGNAT2 (33 aa underlined with green) and NSI (57 aa underlined with orange) were predicted by TargetP-2.0 website (https://services.healthtech.dtu.dk/services/TargetP-2.0/).

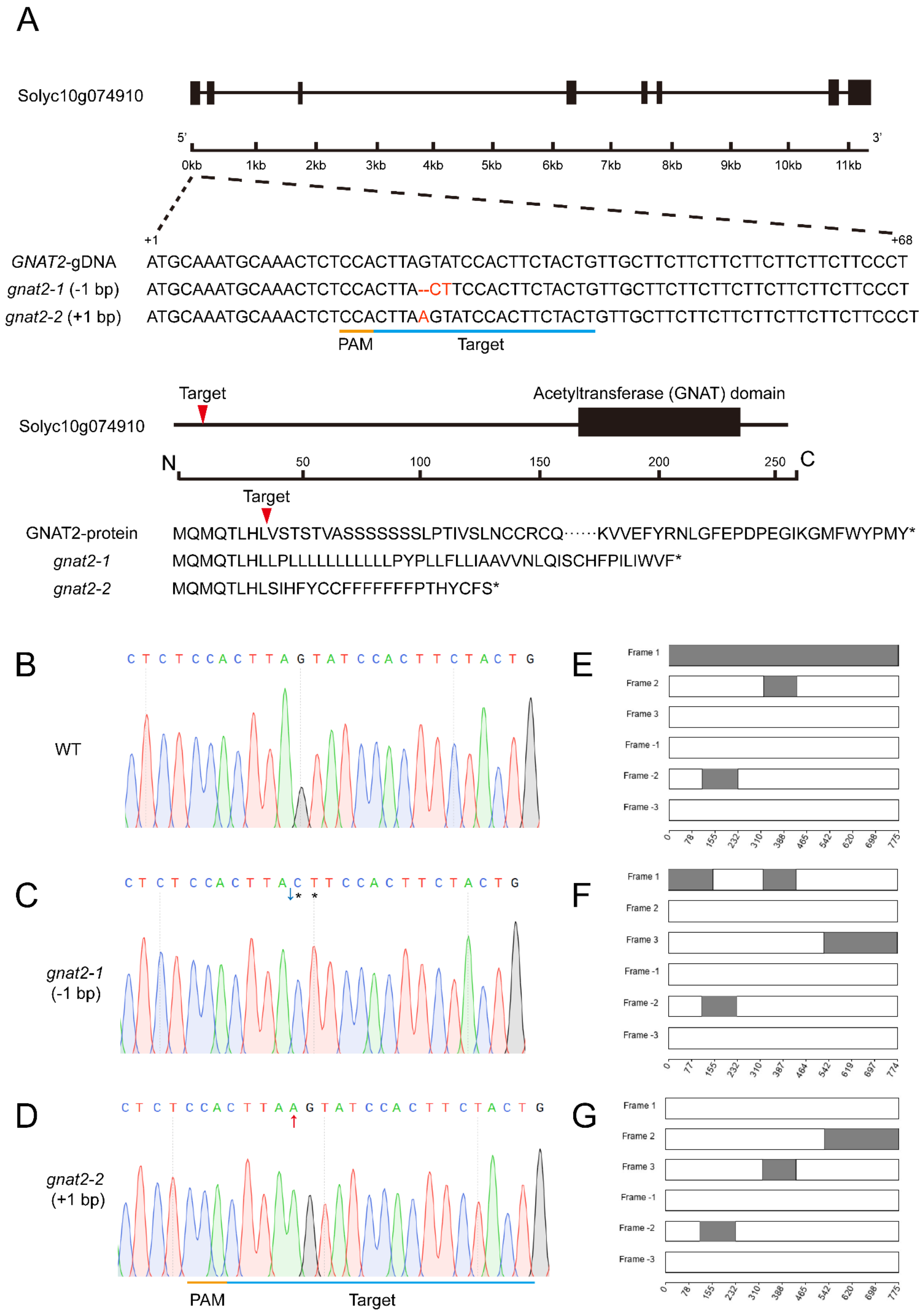

Supplemental Figure 2. Sequence confirmation and ORF prediction of *GNAT2* CRISPR lines.
(A) Gene editing patterns and translated-protein prediction of *GNAT2* CRISPR/Cas9 knockout lines. Target and PAM sequences are underlined with blue and orange, respectively. Red letters indicate the changed bases. Sequence confirmation of (B) WT, (C) *gnat2-1*, and (D) *gnat2-2* lines in individual T2 plants. Substitutions, deletions and insertions of nucleotides are marked with stars, blue and red arrows, respectively. Open reading frame predictions of (E) WT, (F) *gnat2-1*, and (G) *gnat2-2* lines are performed with TBtools software (version 1.068, South China Agr Univ, Guangzhou, China).

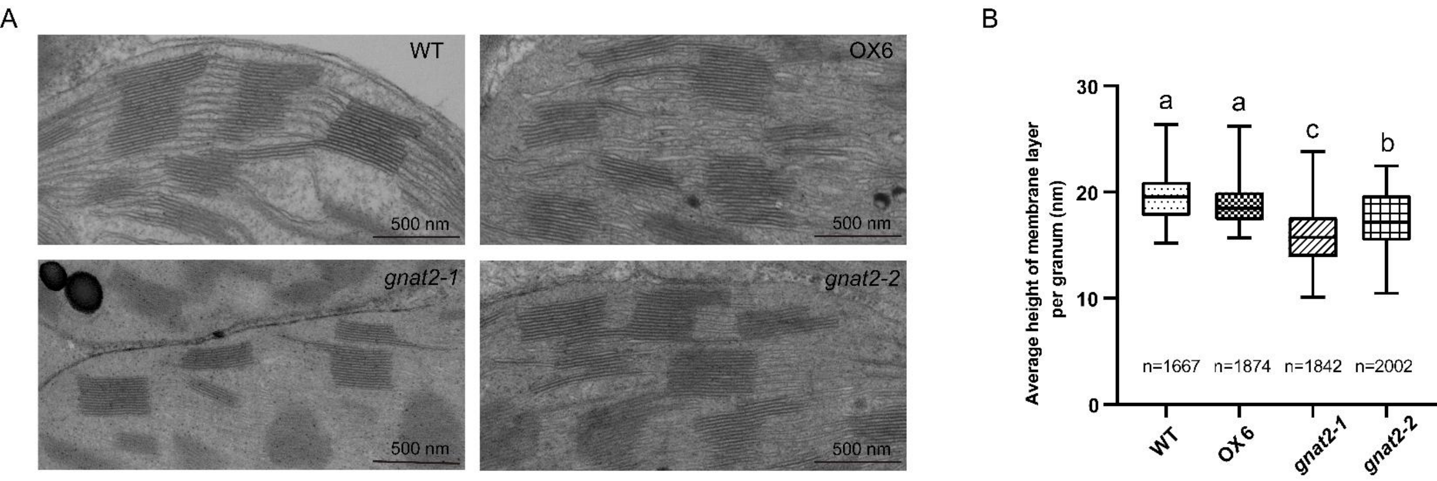

Supplemental Figure 3. Electron microscopy of thylakoids of different lines.
(A) Transmission electron microscopy (TEM) analysis of the WT, OX6, *gnat2-1* and *gnat2-2* chloroplasts. Leaves of different lines in a normal light environment were prepared as thin section samples. Bar = 500 nm. (B) Average heights per granum membrane layer for different lines. Values are means ± SD. Different letters indicate significant differences ($P < 0.05$), as determined by one-way ANOVA using Tukey's multiple comparisons test.

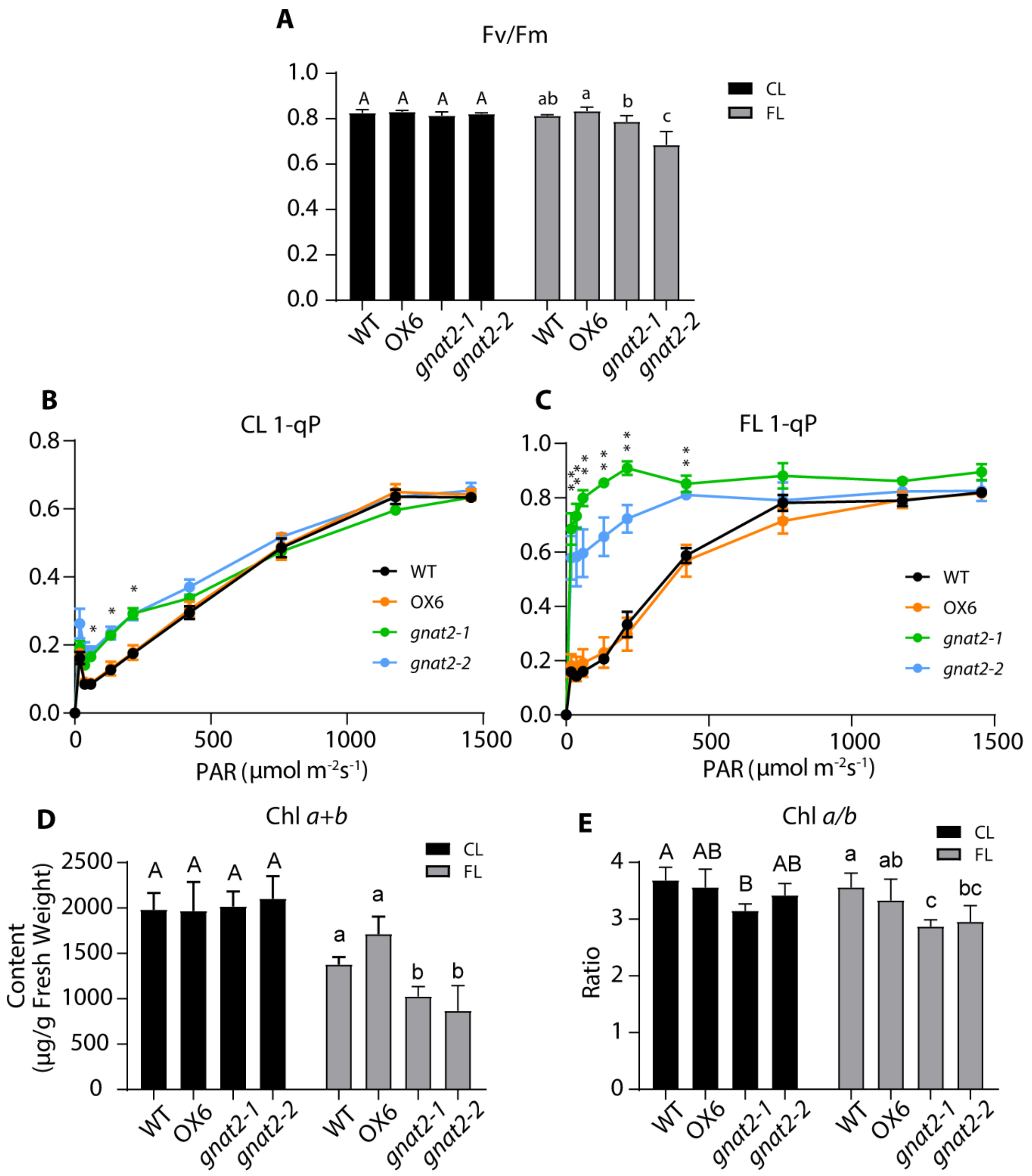

Supplemental Figure 4. Chlorophyll fluorescence and chlorophyll content measurements of leaves of *GNAT2* transgenic plants under different light conditions.

(A) Fv/Fm values of different lines under different light conditions. Error bars show the mean ± SD (n = 3 to 5). Different letters are significantly different from each other (P < 0.05). Data were analyzed by two-way ANOVA using Tukey's multiple comparisons test. (B) Chlorophyll fluorescence measurements of 1-qP for different lines under constant or (C) fluctuating light environment. Standard deviations were determined from three independent plants. Data are means ± SEM (n = 3 to 5). P-values are from two-sided Student's t-tests; * indicates P < 0.05. The content of (D) total chlorophyll and (E) chlorophyll *a/b* ratio were determined after 14 days treatment for different tomato lines. Error bars show the mean ± SD (n = 4 to 5). Different letters are significantly different from each other in groups (P < 0.05). Data were analyzed by two-way ANOVA using Tukey's multiple comparisons test.

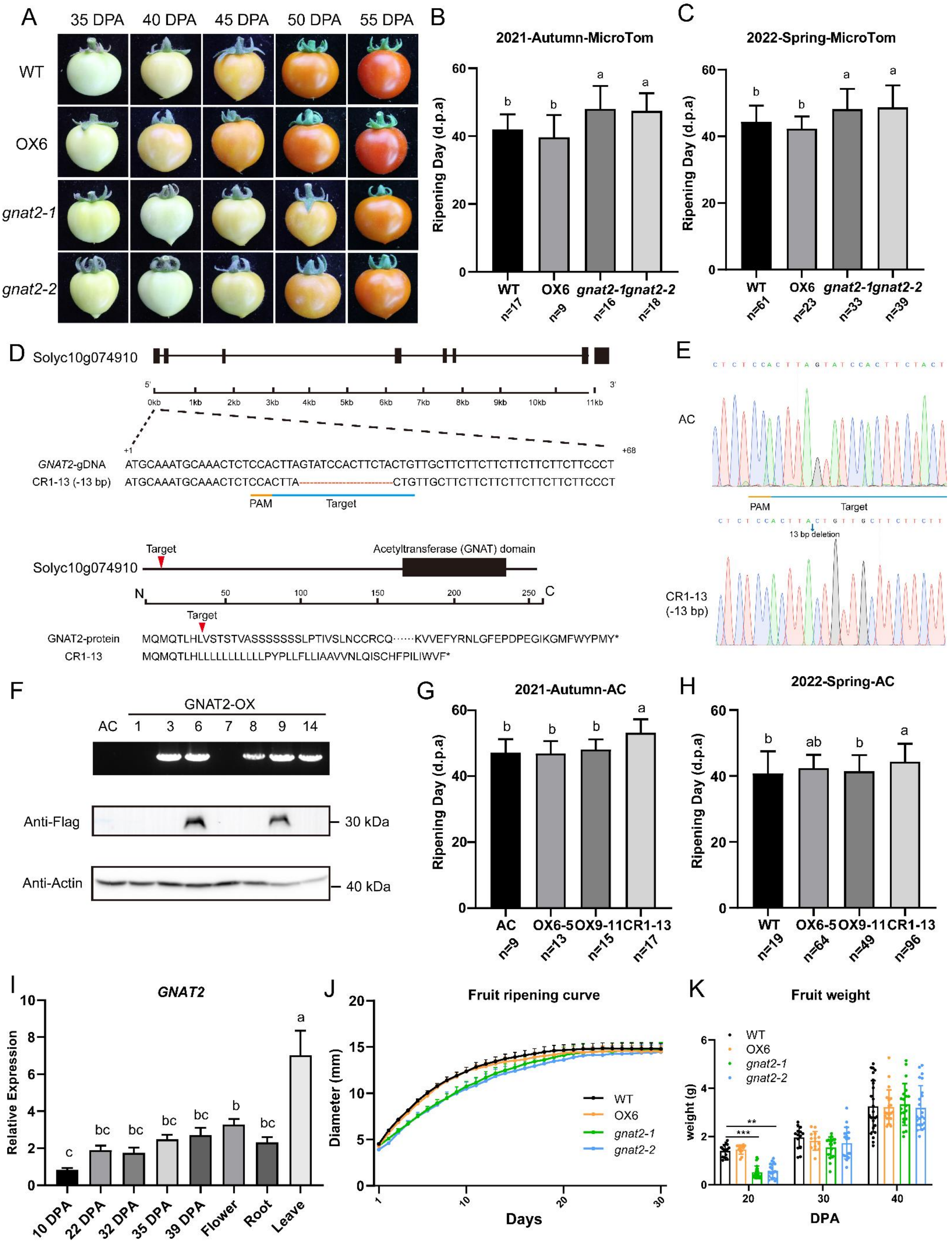

Supplemental Figure 5. Identification of *GNAT2* transgenic lines in tomato cultivar Micro Tom and Ailsa Craig (AC) and statistics of fruit ripening time.

(A) Phenotype of different Micro Tom lines in tomato fruit ripening. OX6, *gnat2-1* and *gnat2-2* fruits were compared with WT fruits at 35, 40, 45, 50 and 55 DPA (days post-anthesis) ripening stages, showing a different color from 35 DPA. The statistics of tomato fruit ripening time in (B) Autumn 2021 and (C) Spring 2022, tomato cultivar Micro Tom were analysis. d.p.a., day post anthesis. (D) Gene editing patterns and translated-protein prediction of *GNAT2* CRISPR/Cas9 knockout AC lines. Target and PAM sequences are underlined with blue and orange, respectively. (E) Sequence confirmation of AC and CR1-13 line in individual T2 plants. (F) PCR (top panel) and immunoblot analysis (medium panel) of T0 generation of *GNAT2* overexpression AC lines. The *GNAT2* overexpression lines 6 and 9 were both positive based on PCR and immunoblot analysis. T2 homozygous lines OX6-5 and OX9-11 were used for subsequent experiments. The statistics of tomato fruit ripening time in (G) Autumn 2021 and (H) Spring 2022 in tomato cultivar AC. Different letters indicate significant differences by one-way ANOVA using Tukey's multiple comparisons test. (I) qRT-PCR analysis of *GNAT2* expression in different tomato tissues. Tomato housekeeping gene *SlActin* (Solyc11g005330) and *SlEFa* (Solyc06g005060) were used as internal control. At least three independent biological replicates were measured for each line. Error bars show the mean ± SEM. Different letters are significantly different from each other in groups ($P < 0.05$). Data were analyzed by two-way ANOVA using Tukey's multiple comparisons test.(J) Fruit ripening curve of different Micro Tom lines. Flowers were tagged at the date of anthesis and the fruit diameter was counted at same time daily for 30 days when the ovary expanded to 4-5 mm (about 7-10 DPA). At least ten fruits in each line were measured. Error bars show the mean ± SEM. (K) Fruit weight of different Micro Tom lines. Flowers were tagged at the date of anthesis, then were harvested and weighted after 20, 30 or 40 days. Error bars show the mean ± SD (n = 12 to 25). Asterisks indicate significant differences (**$P < 0.01$, ***$P < 0.001$) compared to WT in 20 DPA, as determined by two-way ANOVA using Tukey's multiple comparisons test. All *P* values were shown in Supplemental Dataset 2.

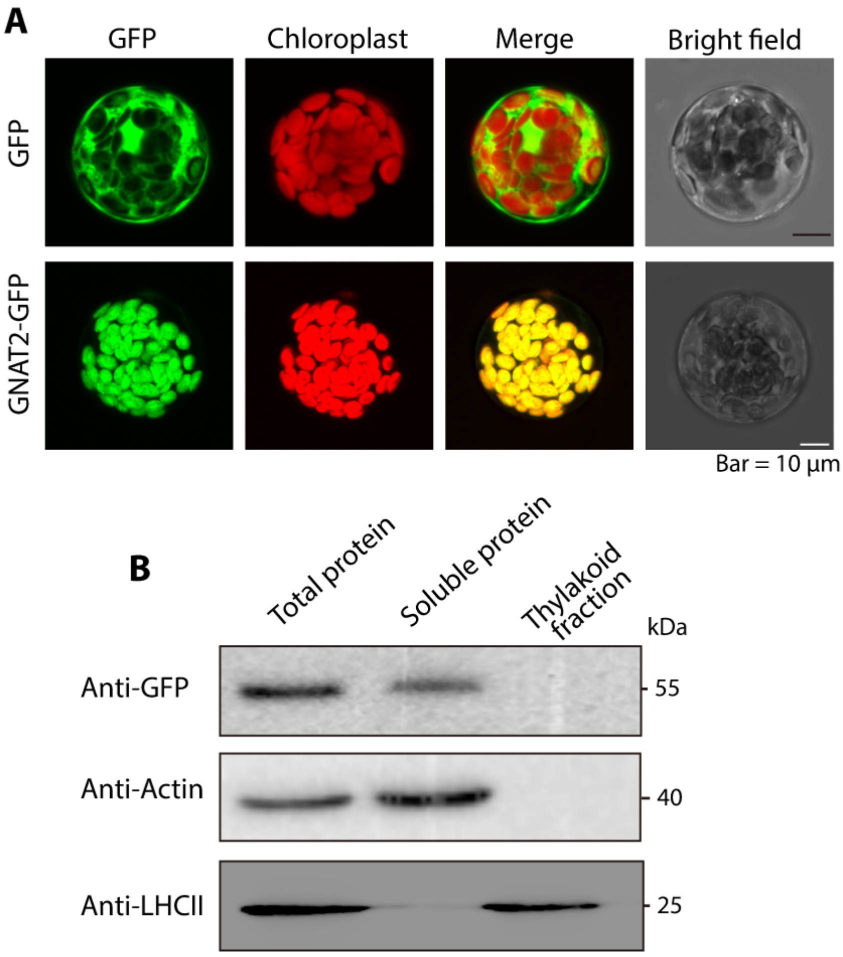

Supplemental Figure 6. Subcellular localization of GNAT2.
(A) Subcellular localization of GNAT2 by GFP fluorescence. Green indicates GFP, red shows chloroplast autofluorescence and yellow shows co-localization of the merged images. GFP (*$35S_{pro}$:GFP*) protein and GNAT2-GFP (*$35S_{pro}$:GNAT2:GFP*) fusion protein were transiently expressed in tomato protoplasts, respectively. Bar = 10 µm. (B) Immunolocalization analysis of GNAT2. Total protein, soluble protein and thylakoid fractions were isolated from transient-transfected tomato protoplasts expressing GNAT2-GFP (*$35S_{pro}$:GNAT2:GFP*) fusion protein and were subjected to immunoblot analysis with specific antibody or antisera again GFP, actin and LHCII. Equal amounts of protein (10 µg) were loaded in each lane.

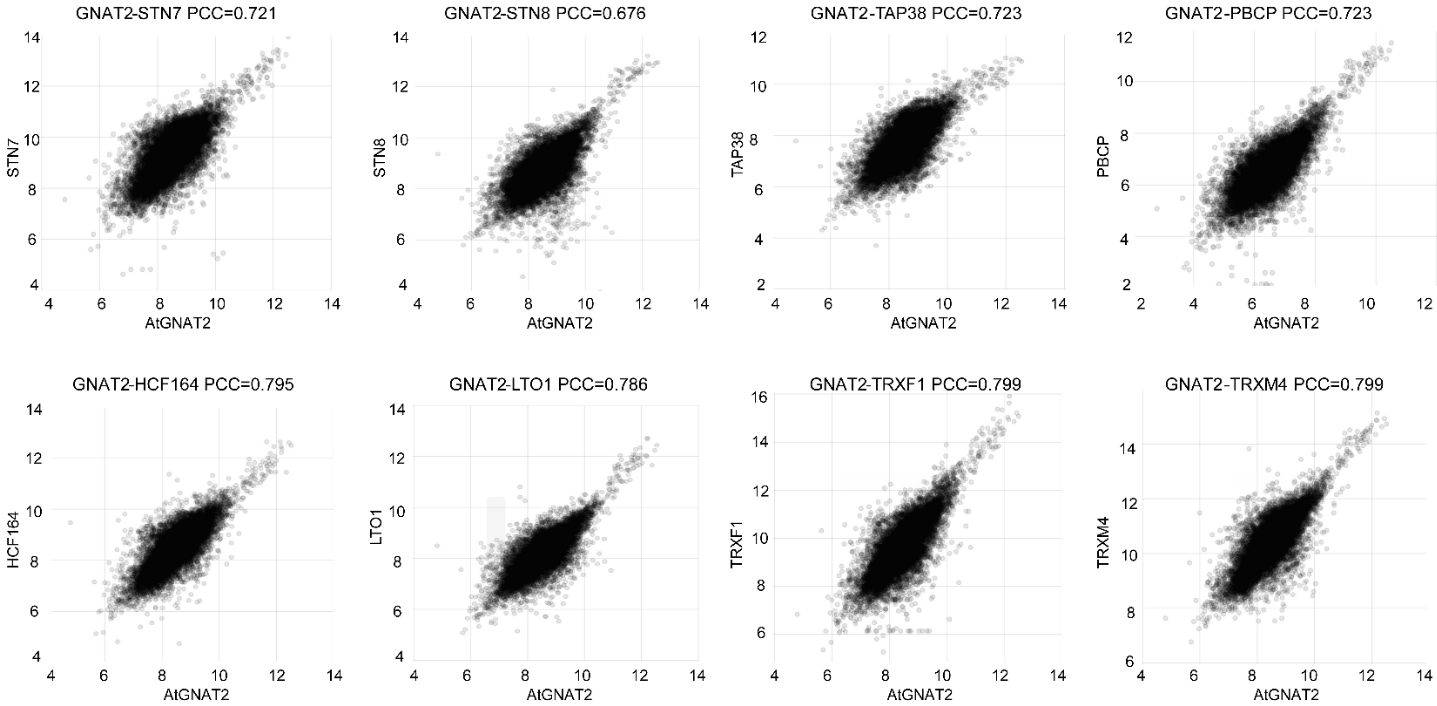

Supplemental Figure 7. Co-expression analysis based on the RNAseq database of *NSI*.

Co-expression data with RNAseq of *NSI* were obtained from the current version (11.1) of the ATTED-II website (http://atted.jp/). STN7, LHCII kinase; STN8, PSII core kinase; TAP38, LHCII phosphatase; PCBC, PSII core phosphatase; HCF164, thioredoxin superfamily protein; LTO1, thioredoxin-like protein; TRXF1, thioredoxin F-type protein ; TRXM4, thioredoxin M-type protein. The PCC values of RNAseq database are shown at the top of each panel.

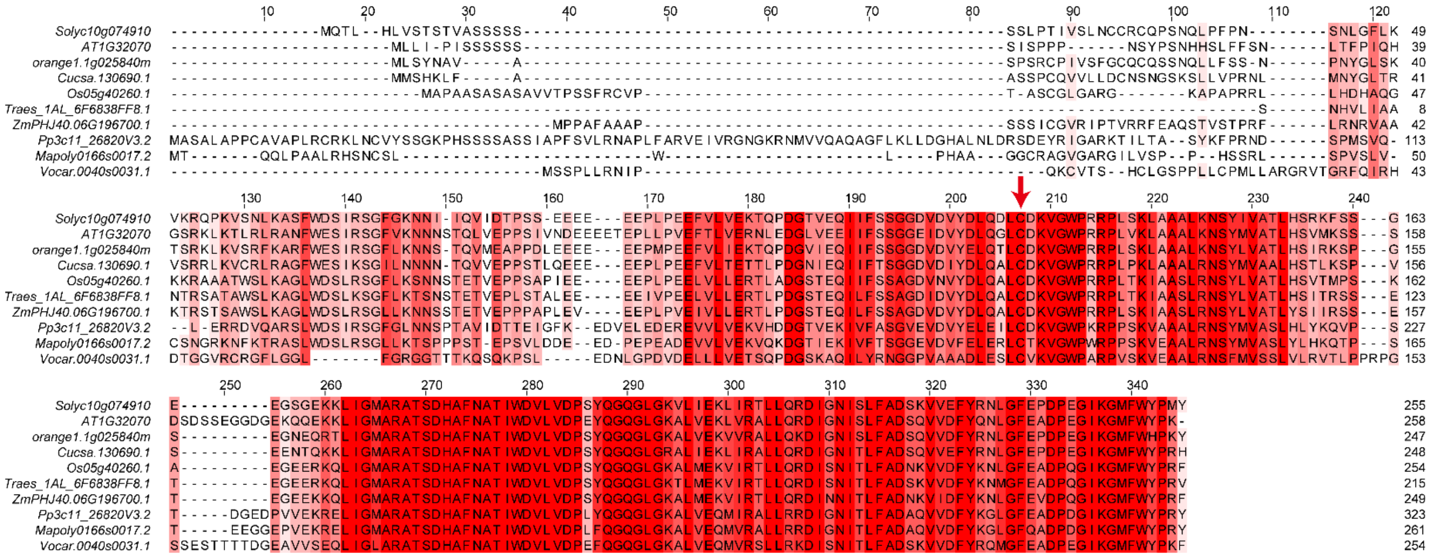

Supplemental Figure 8. Multiple amino acid sequence alignment of GNAT.
Multiple amino acid sequence alignment of tomato GNAT2 (Solyc10g074910) and homologs from *Arabidopsis thaliana* (AT1G32070), *Citrus sinensis* (orange1.1g025840m), *Cucumis sativus* (Cucsa.130690.1), *Zea mays* (ZmPHJ40.06G196700.1), *Triticum aestivum* (Traes_1AL_6F6838FF8.1), *Oryza sativa* (Os05g40260.1), *Volvox carteri* (Vocar.0040s0031.1), *Marchantia polymorpha* (Mapoly0166s0017.2) and *Physcomitrium patens* (Pp3c11_26820V3.2). Identical amino acids are marked in red; similar amino acids are pink. The amino acid sequence alignment was performed with Clustal Omega (https://www.ebi.ac.uk/Tools/msa/clustalo/) and displayed with Jalview.

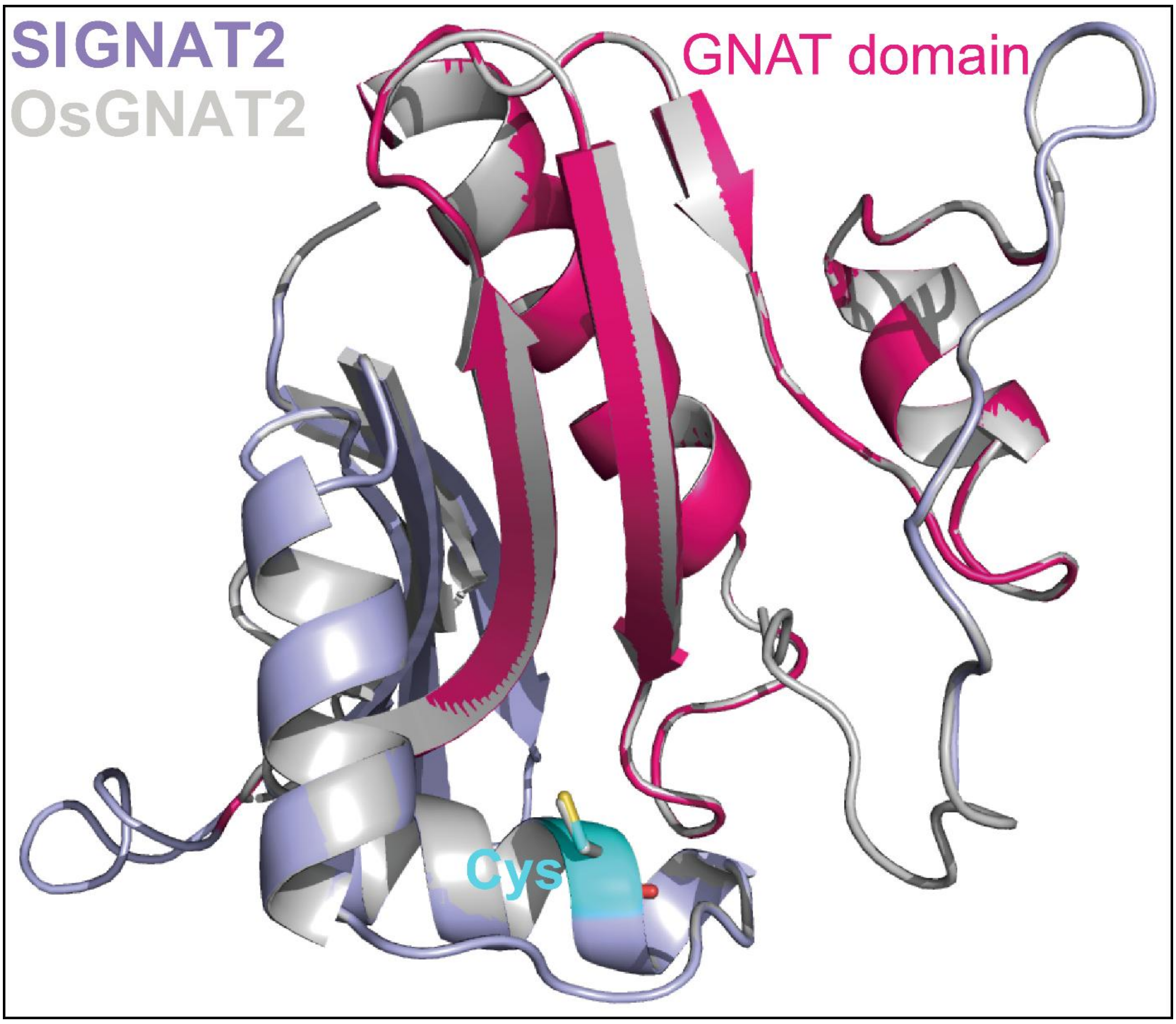

Supplemental Figure 9. Structure of GNAT2 predicted by SWISS-MODEL (https://swissmodel.expasy.org/). The SlGNAT2 structure was predicted by SWISS-model website (https://swissmodel.expasy.org) with the template of OsGNAT2 (6K5M). Structural comparison of these two structures was performed with PyMOL software as cartoon model. The conserved cysteine and GNAT domain of SlGNAT2 are marked with cyan sticks and hotpink cartoon, respectively.

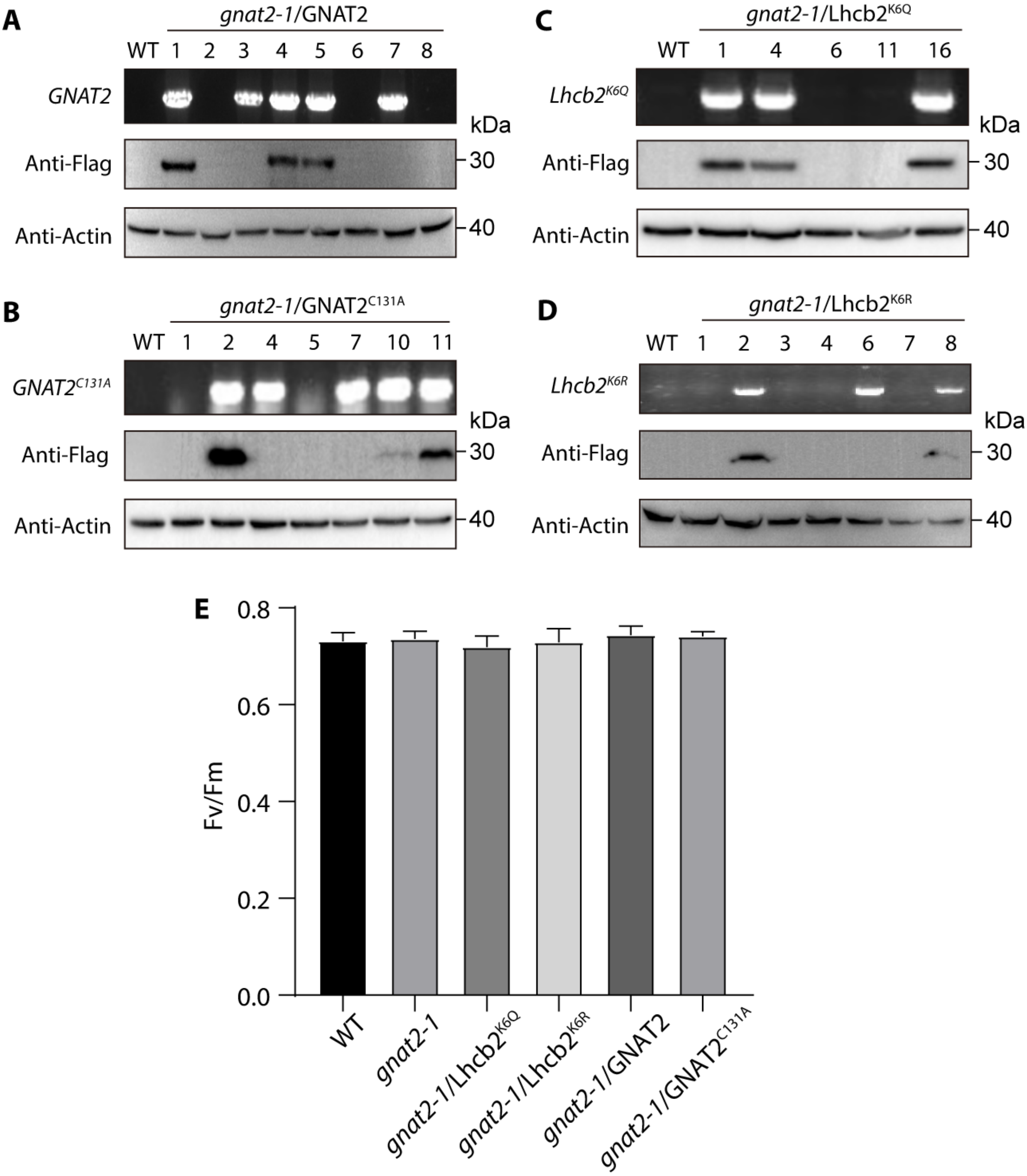

Supplemental Figure 10. Identification of *gnat2-1*/Lhcb2^{K6Q}, *gnat2-1*/Lhcb2^{K6R}, *gnat2-1*/GNAT2 and *gnat2-1*/GNAT2^{C131A} transgenic lines.

PCR (top panel) and immunoblot analysis (medium panel) of (A) *gnat2-1*/GNAT2, (B) *gnat2-1*/GNAT2^{C131A}, (C) *gnat2-1*/Lhcb2^{K6Q} and (D) *gnat2-1*/Lhcb2^{K6R}. Lines 1,4 and 5 of *gnat2-1*/GNAT2; lines 2 and 11 of *gnat2-1*/GNAT2^{C131A}; lines 1, 4 and 16 of *gnat2-1*/Lhcb2^{K6Q} and lines 2 and 8 of *gnat2-1*/Lhcb2^{K6R} were both positive based on PCR and immunoblot analysis. (E) Fv/Fm fluorescence ratios of WT, *gnat2-1*, *gnat2-1*/Lhcb2^{K6Q}, *gnat2-1*/Lhcb2^{K6R}, *gnat2-1*/GNAT2 and *gnat2-1*/GNAT2^{C131A} are not significantly different. At least eight independent biological replicates were measured for each line. Values are means ± SD. *P* values were shown in Supplemental Dataset 2 (one-way ANOVA using Tukey's multiple comparisons test).

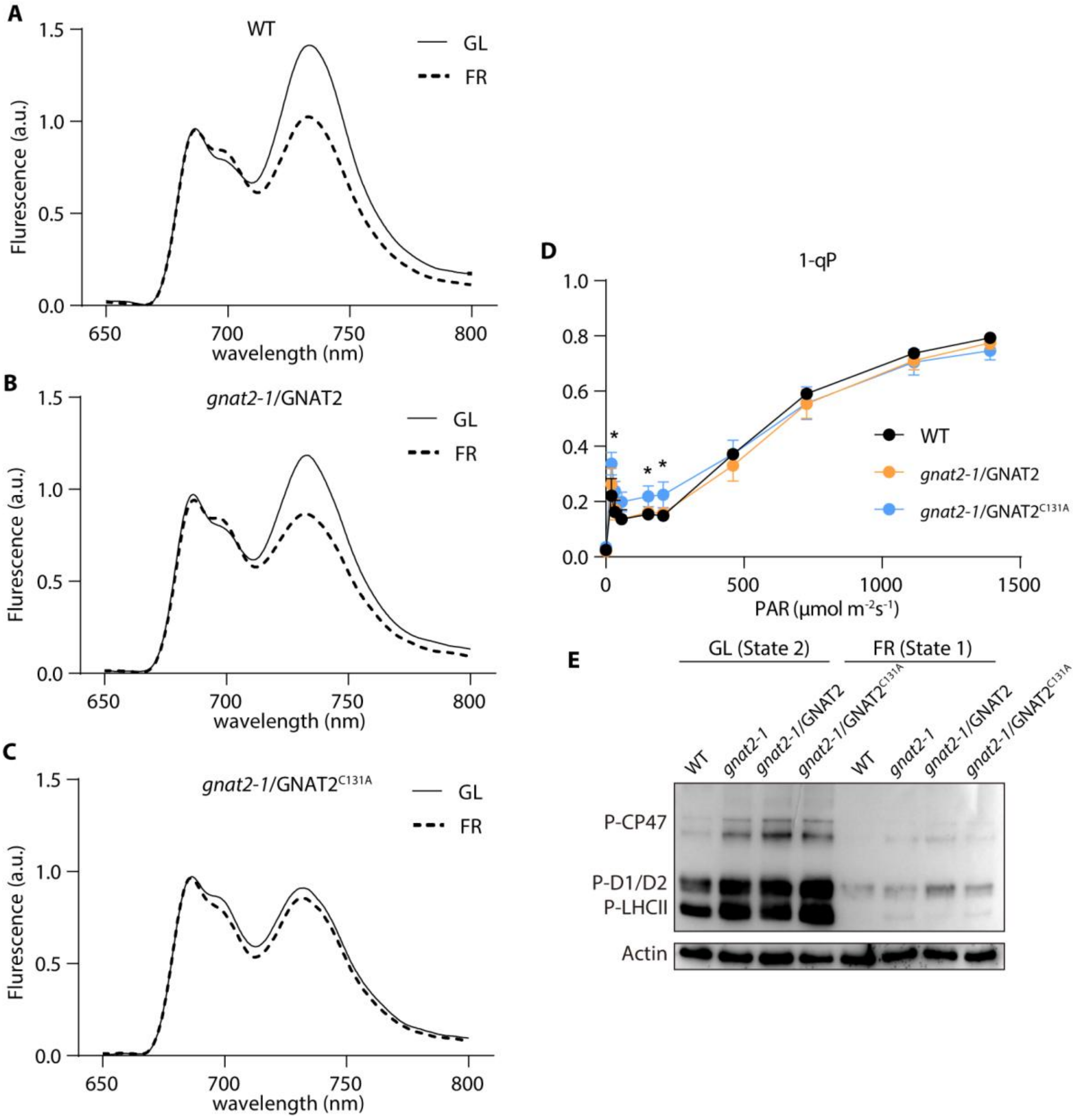

Supplemental Figure 11. [131]Cys of GNAT2 affect state transitions *in vivo*.

77K fluorescence emission spectra of thylakoids from (A) WT, (B) *gnat2-1*/GNAT2 and (C) *gnat2-1*/GNAT2^{C131A} under growth light (GL, solid lines) and far-red light (FR, dashed lines). Fluorescence emission around 685 nm originates from PSII and fluorescence emission around 735 nm originates from PSI. The 77K fluorescence spectra were normalized at 685 nm. a.u., arbitrary units. (D) 1-qP values of different lines. (E) Thylakoid membrane proteins extracted from WT, *gnat2-1*, *gnat2-1*/GNAT2 and *gnat2-1*/GNAT2^{C131A} in the GL or in the FR treatment were separated by 10% SDS-PAGE and immunoblotted with an anti-phosphothreonine antiserum (Cell Signaling Technology, 1:10000).

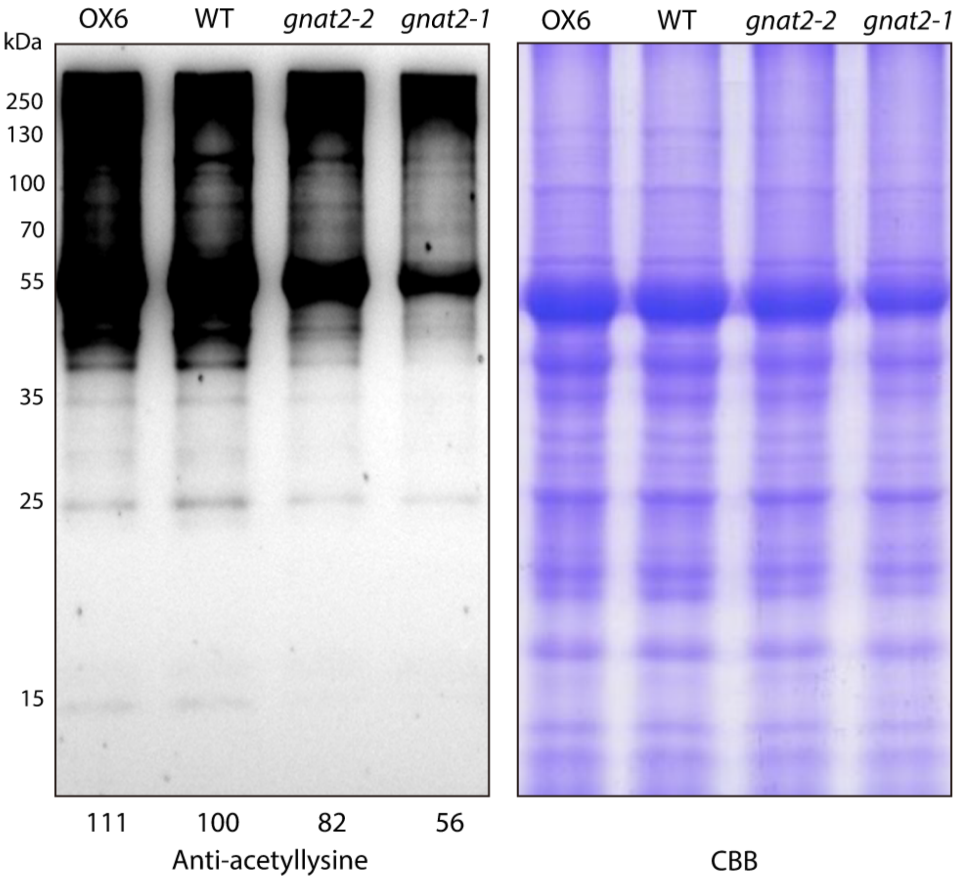

Supplemental Figure 12. Lysine Acetylation Activity of GNAT2.
The lysine acetylation levels of total protein were detected from 4-week-old GNAT2 transgenic and wild-type plants. Proteins were separated by 12% SDS-PAGE gel and immunoblotted with an anti-acetyllysine antibody (PTM-101; Lot:22838591HB14; 1:1000 dilution). Equal amounts of protein (20 µg) were loaded in each lane. Coomassie bright blue staining was used for the control.

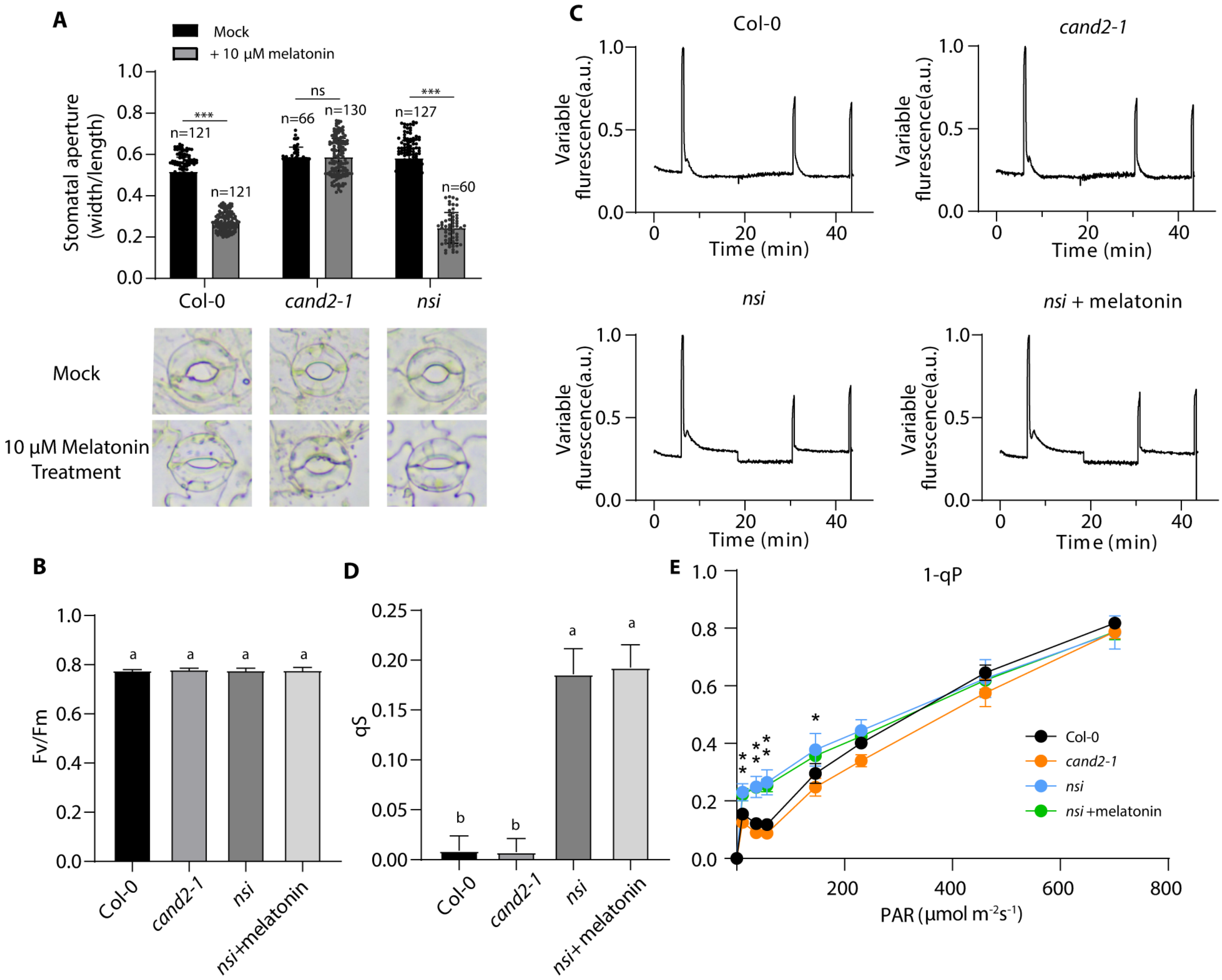

Supplemental Figure 13. Melatonin is not involved in state transitions.

(A) Effect of 10 µM melatonin treatment in stomatal aperture of Col-0 (*Arabidopsis* wild type plant), *cand2-1 and nsi*. (B) Fv/Fm values of Col-0, *cand2-1*, *nsi* and *nsi* treated with exogenous melatonin under growth light conditions (200 µmol m^{-2} s^{-1}). (C) Fluorescence curve analyses of state transitions in Col-0, *cand2-1, nsi* and *nsi* treated with exogenous melatonin. (D) qT values. Each treatment included at least 3 independent biological replicates. Values denote the means ± SD. Significant differences were determined using one-way ANOVA using Tukey's multiple comparisons test. *P* values were shown in Supplemental Dataset 2. (E) 1-qP values.

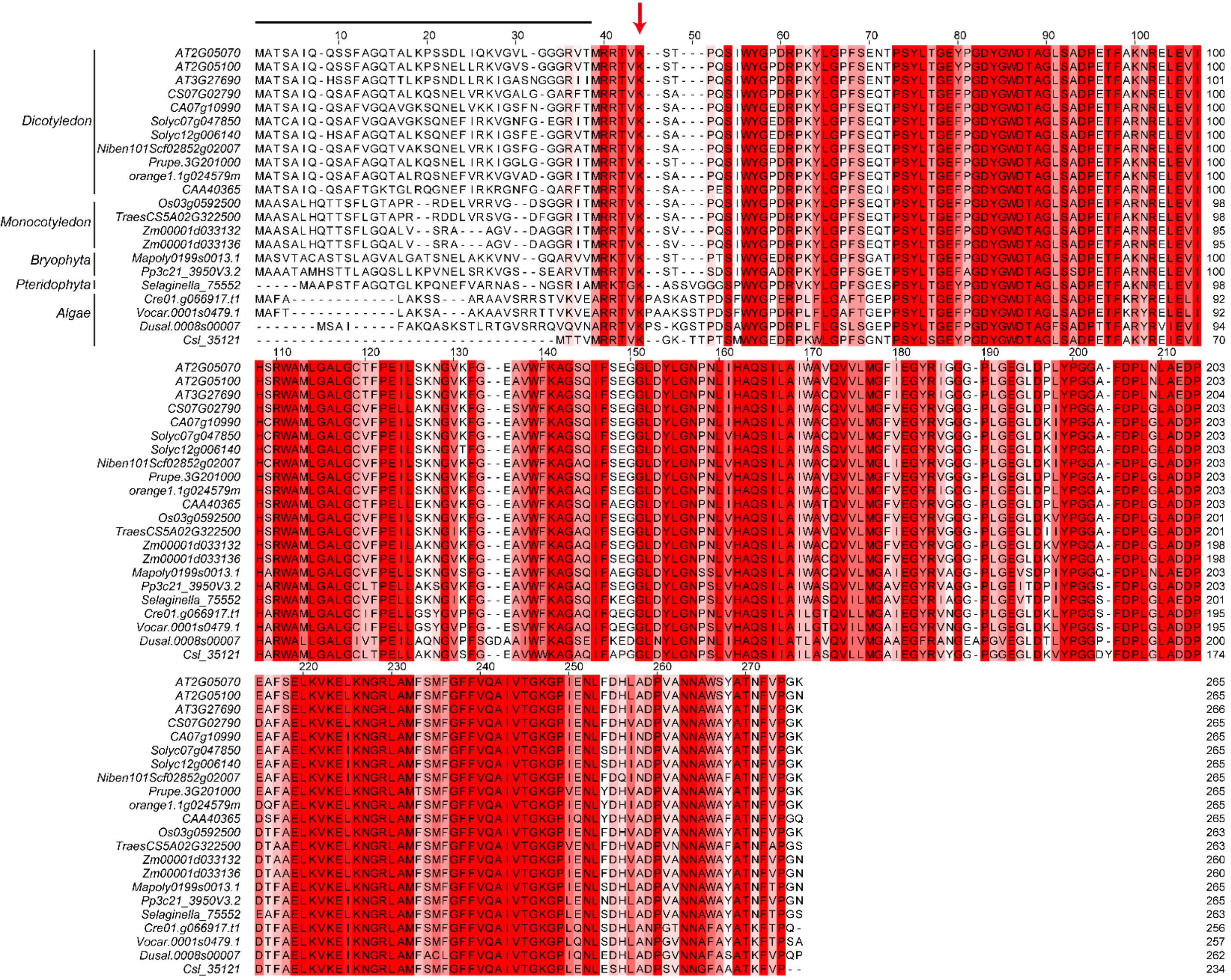

Supplemental Figure 14. Multiple amino acid sequence alignments of Lhcb2 isoforms.
Multiple amino acid sequence alignments of tomato Lhcb2 (SlLhcb2.1, Solyc07g047850 and its homologs SlLhcb2.2 with ID Solyc12g006140) and homologs from *Arabidopsis thaliana* (AtLhcb2.1, AtLhcb2.2 and AtLhcb2.3, with IDs AT2G05100, AT2G05070 and AT3G27690, respectively, *Cucumis sativus* (Cs07G02790), *Nicotiana tabacum* (Niben101Scf02852g02007), *Pisum sativum* (CAA40365), *Capsicum annuum* (Ca07g10990), *Prunus persica* (Prupe.3G201000), *Citrus sinensis* (orange1.1g024579m), *Zea mays* (Zm00001d033132 and Zm00001d033136), *Oryza sativa* (Os03g0592500), *Triticum aestivum* L. (TraesCS5A02G322500*),* *Chlamydomonas reinhardtii* (Cre01.g066917.t1), *Coccomyxa subellipsoidea* C-169 (Csl_35121), *Dunaliella salina* (Dusal.0008s00007), *Volvox carteri* (Vocar.0001s0479.1), *Marchantia polymorpha* (Mapoly0199s0013.1), *Physcomitrium patens* (Pp3c21_3950V3.2) and *Selaginella moellendorffii* (Selaginella_75552). Botanical classifications are listed on the left. Identical amino acids are marked with red; similar amino acids are pink. The predicted transit peptide of Lhcb2 is marked by a black line on top, and the red arrow indicates the conserved lysine ([6]Lys-AtLhcb2) site in different Lhcb2 isoforms. The amino acid sequence alignment was performed with Clustal Omega (https://www.ebi.ac.uk/Tools/msa/clustalo/) and displayed with Jalview.

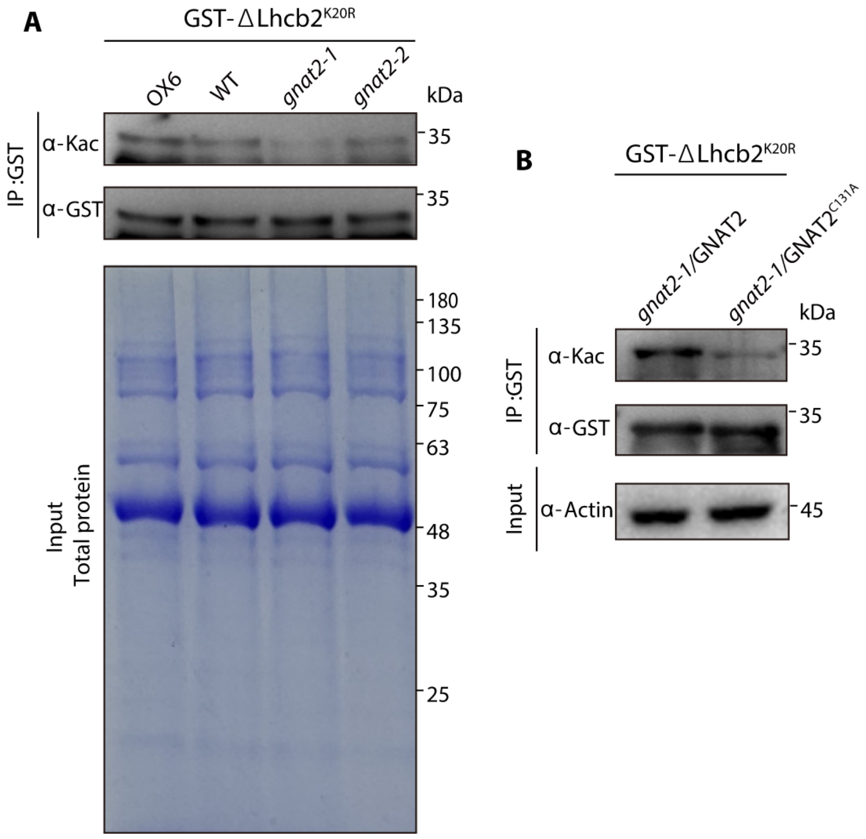

Supplemental Figure 15. The acetylation levels of ^{6}Lys in different lines *in vivo*.

(A) Assay of the acetylation level of ΔLhcb2^{K20R} protein in WT, OX6, *gnat2-1* and *gnat2-2* lines. (B) Assay of the acetylation level of ΔLhcb2^{K20R} protein in *gnat2-1*/GNAT2 and *gnat2-1*/GNAT2^{C131A} lines. ΔLhcb2^{K20R} protein, which containing only one lysine residue of ^{6}Lys, was used in subsequent experiments had coupled with GST beads was co-incubated with total protein of different lines for 30 min at 30℃, then analyzed by immunoblotting. CBB staining was used for the control.

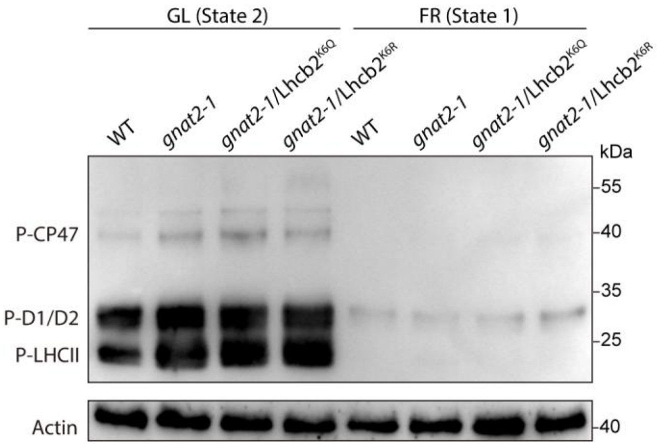

Supplemental Figure 16. Phosphorylation patterns of complementation plants.
Thylakoid membrane proteins extracted from WT, *gnat2-1*, *gnat2-1*/Lhcb2^{K6Q} and *gnat2-1*/Lhcb2^{K6R} in the GL (State 2) or in the FR (State 1) treatment were separated by 10% SDS-PAGE and immunoblotted with an anti-phosphothreonine antiserum (Cell Signaling Technology, 1:10000).
