# Supplementary material for: Chloroplast acetyltransferase GNAT2 acts as a redox-regulated switch for state transitions in tomato
Source: Mol Hortic. 2025 Aug 6;5:39. doi: 10.1186/s43897-025-00164-0 (PMC12326663; doi:10.1186/s43897-025-00164-0)
Supplement: Supplementary file 3 — Supplementary Material 3. [file 43897_2025_164_MOESM3_ESM.pdf]

Supplemental Data Set 1. Details and original figures for immunoblotting

Details for immunoblotting

Marker: 180 kD PageRuler Prestained Protein Ladder, Thermo Scientific, Cat.26616

Membrane: 0.45 µm pore size, Hydrophobic PVDF Transfer Membrane, Merck Millipore, IPVH00010

Blocking buffer: 0.5% nonfat dry milk in TBST (20 mM Tris-HCl, pH 7.5, 150 mM NaCl, and 0.1% Tween 20)

Secondary antibody: HRP Goat Anti-Mouse IgG, ABclonal, AS003, 1:10000 dilution (If no special explanation)

HRP detection: Pro-Light HRP Kit, Tiangen Biotech, PA112

CCD system: Azure Biosystems C300 / Tanon 5200 Multi

Striping buffer: CWBIO, CW0056

Figure 1F

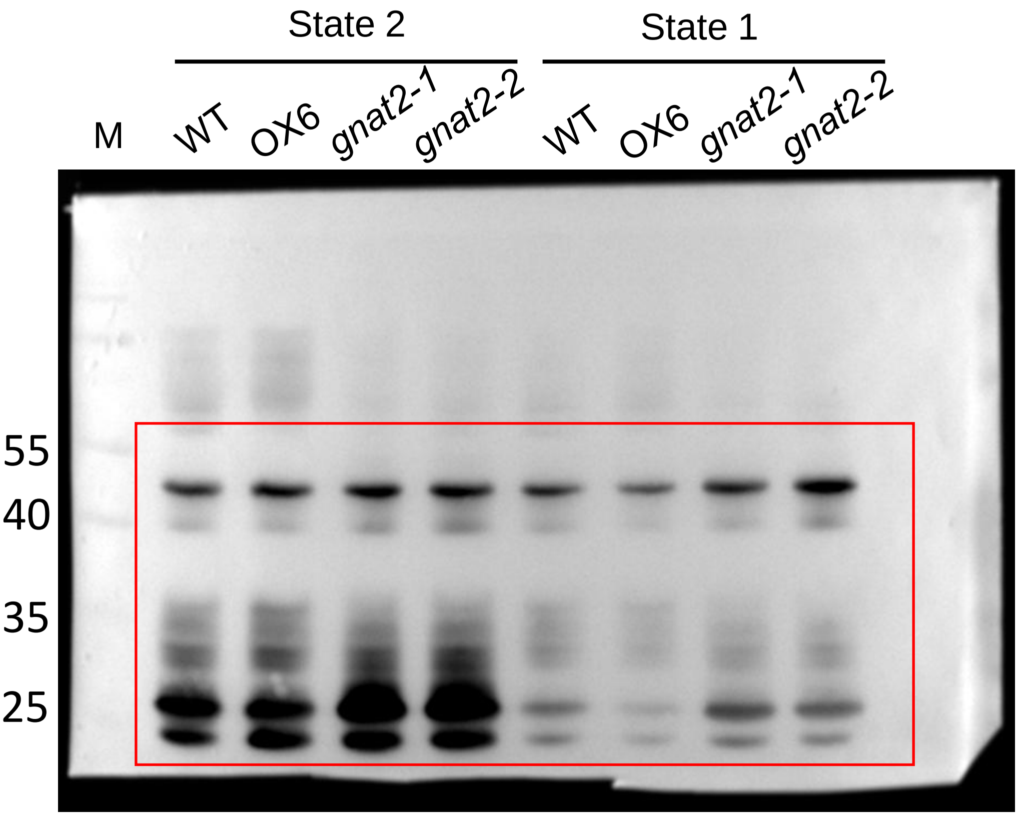

Antibody: Phospho Threonine/Tyrosine
Producer: Cell Signaling Technology
Product code: 9381S
Dilution:1:10000
Exposure time:1 min

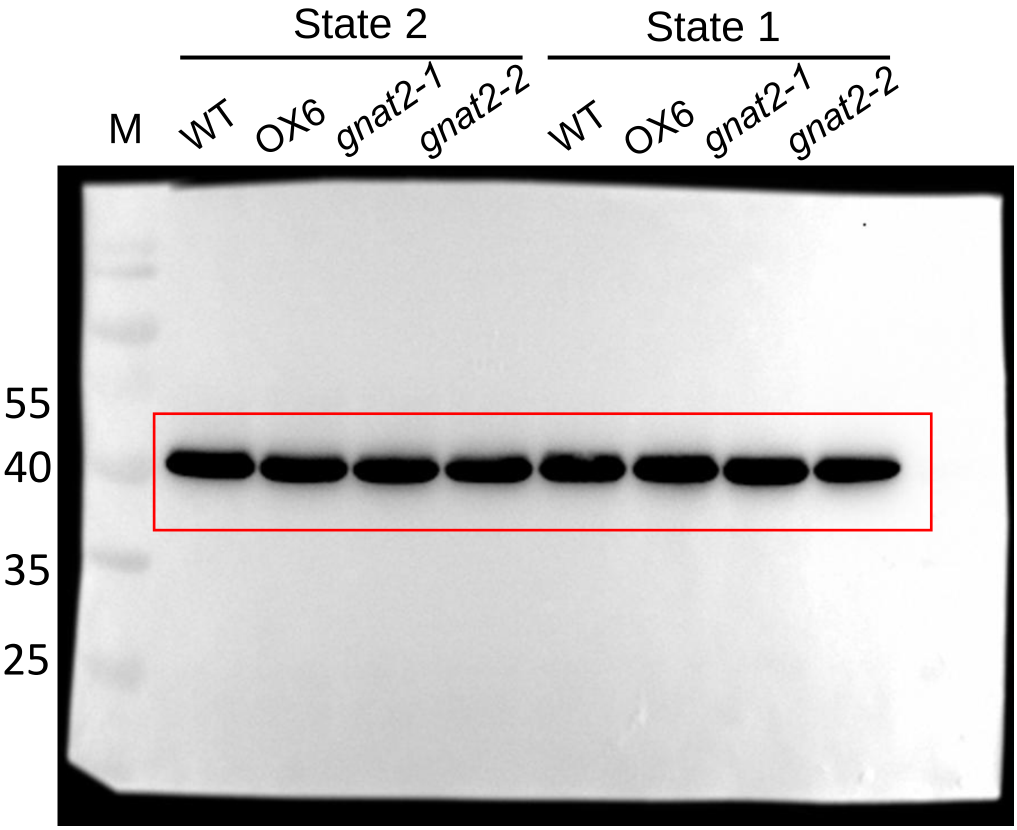

Antibody: Actin
Producer: Abclonal, Wuhan
Product code:AC009
Dilution:1:10000
Exposure time:10 s

Figure 3A

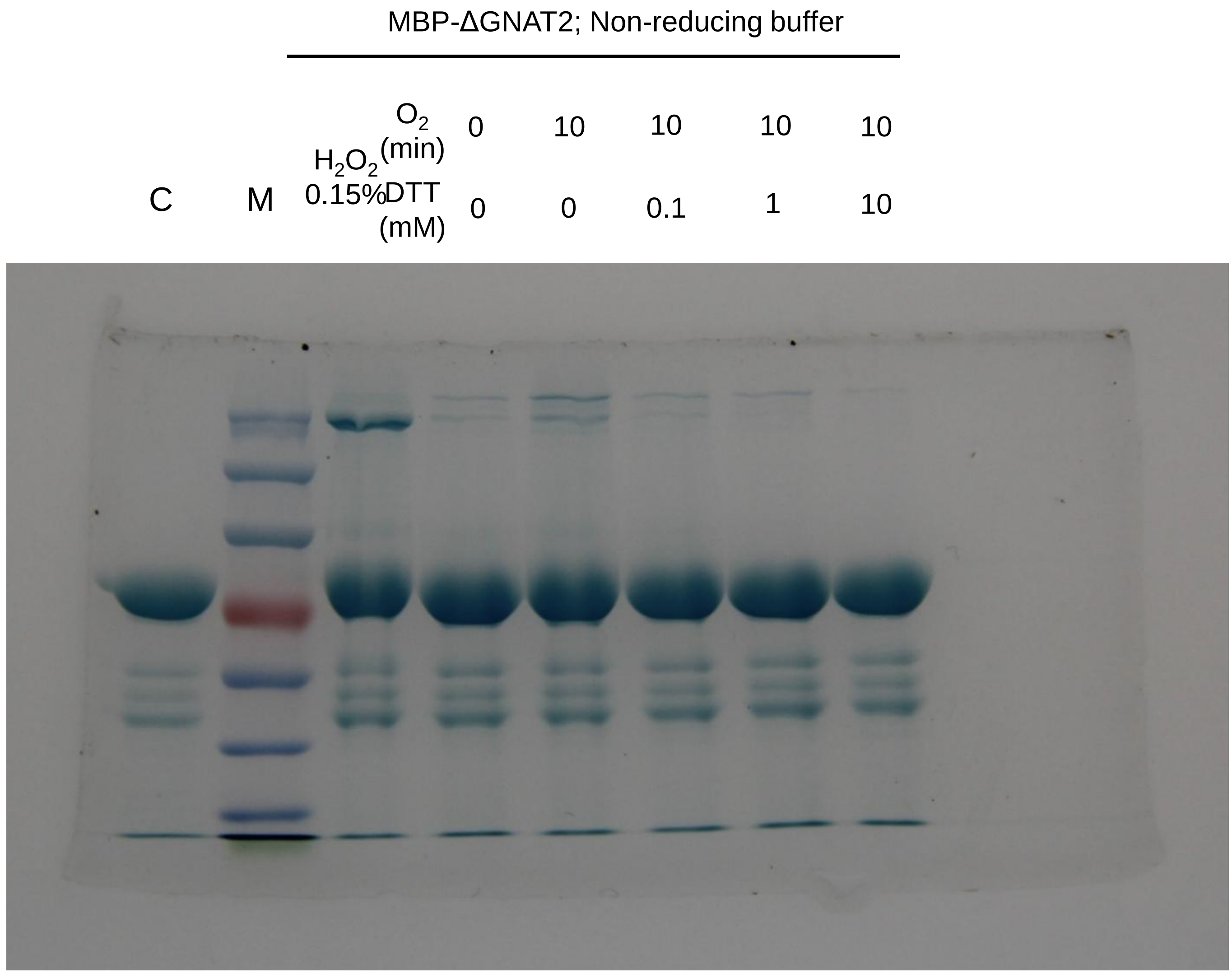

MBP-ΔGNAT2; Non-reducing buffer
O2 (min) 0 10 10 10 10
H2O2 0.15%
DTT (mM) 0 0 0.1 1 10
C
M

Figure 3B

MBP-ΔGNAT2[C131A]; Non-reducing buffer

			O_2 (min)	0	10	10	10	10
C	M	H_2O_2 0.15%	DTT (mM)	0	0	0.1	1	10

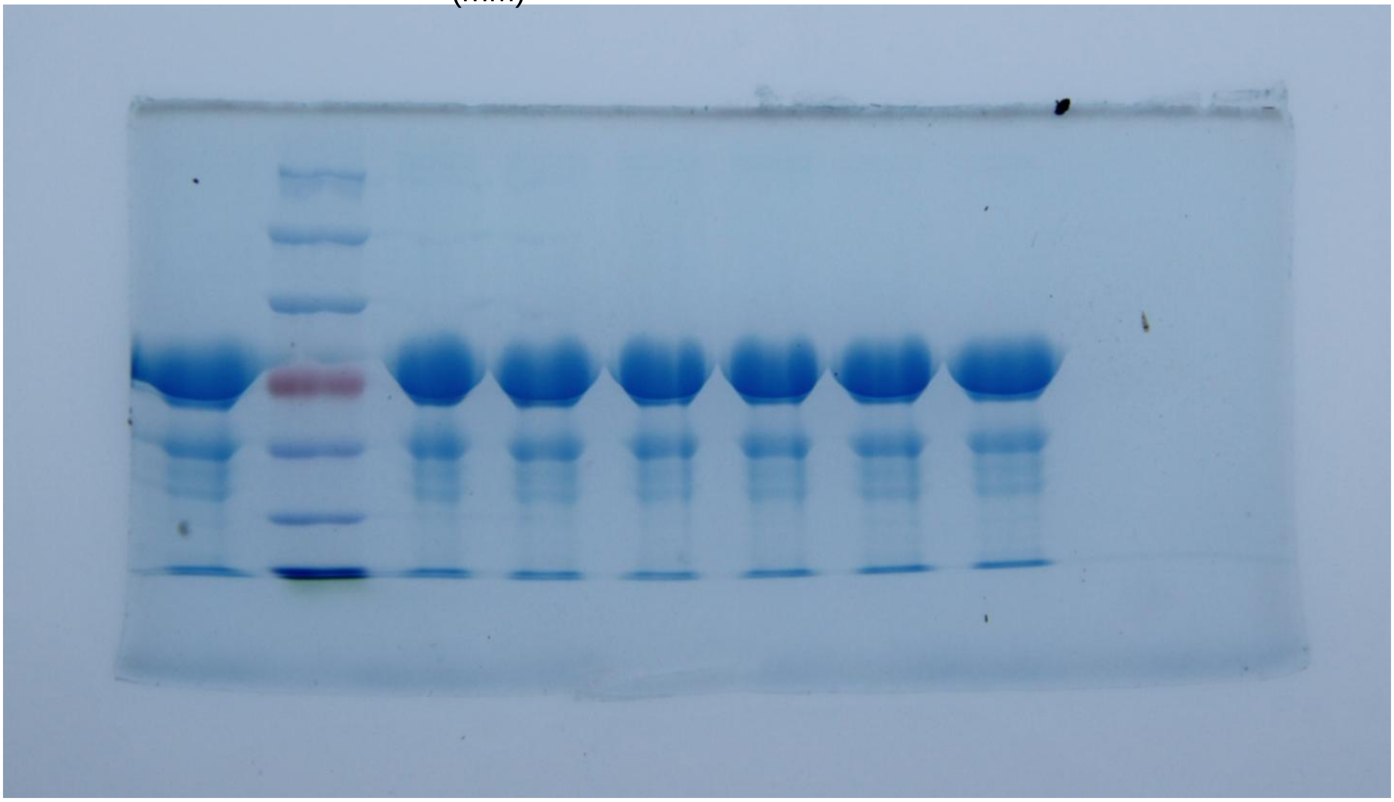

Figure 4A

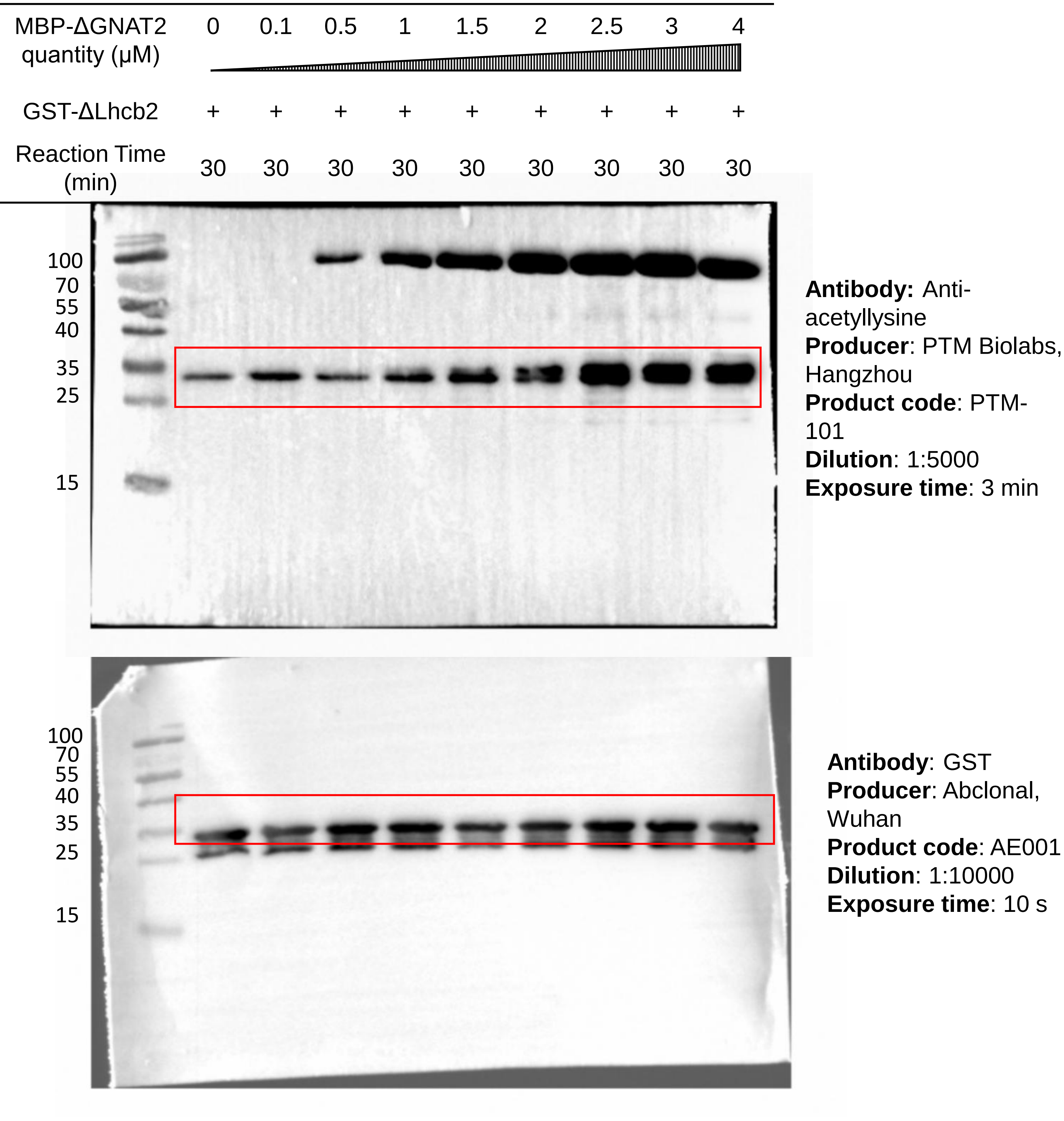
MBP-ΔGNAT2 quantity (μM)
0
0.1
0.5
1
1.5
2
2.5
3
4
GST-ΔLhcb2
+ + + + + + + + +
Reaction Time (min)
30 30 30 30 30 30 30 30 30
100
70
55
40
35
25
15
Antibody: Anti-acetyllysine
Producer: PTM Biolabs, Hangzhou
Product code: PTM-101
Dilution: 1:5000
Exposure time: 3 min
100
70
55
40
35
25
15
Antibody: GST
Producer: Abclonal, Wuhan
Product code: AE001
Dilution: 1:10000
Exposure time: 10 s

Figure 4A

MBP-ΔGNAT2 quantity (μM)	0	0.1	0.5	1	1.5	2	2.5	3	4
GST-ΔLhcb2	+	+	+	+	+	+	+	+	+
Reaction Time (min)	30	30	30	30	30	30	30	30	30

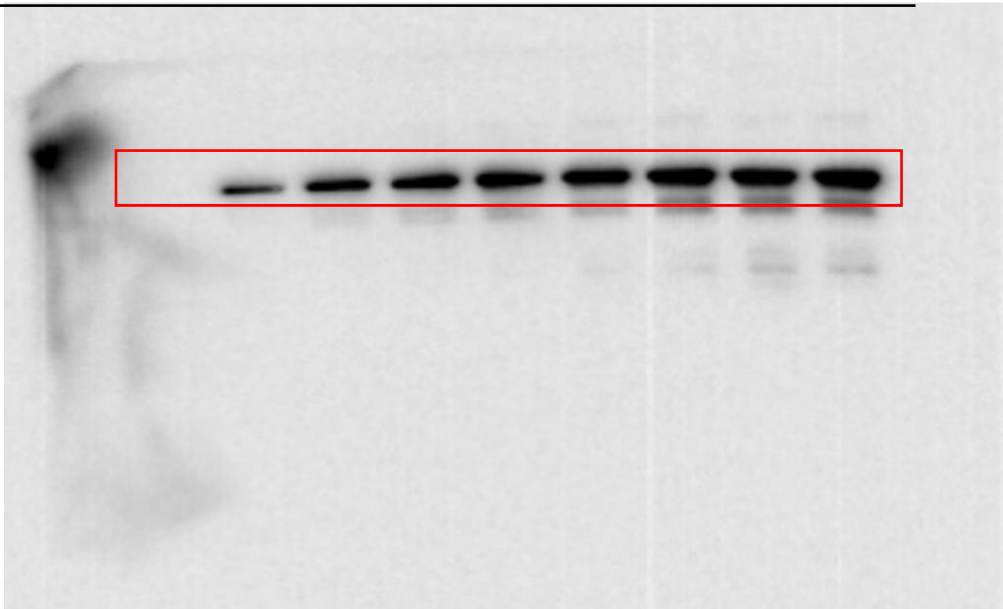

Antibody: MBP
Producer: Abclonal, Wuhan
Product code: AE016
Dilution: 1:10000
Exposure time: 10 s

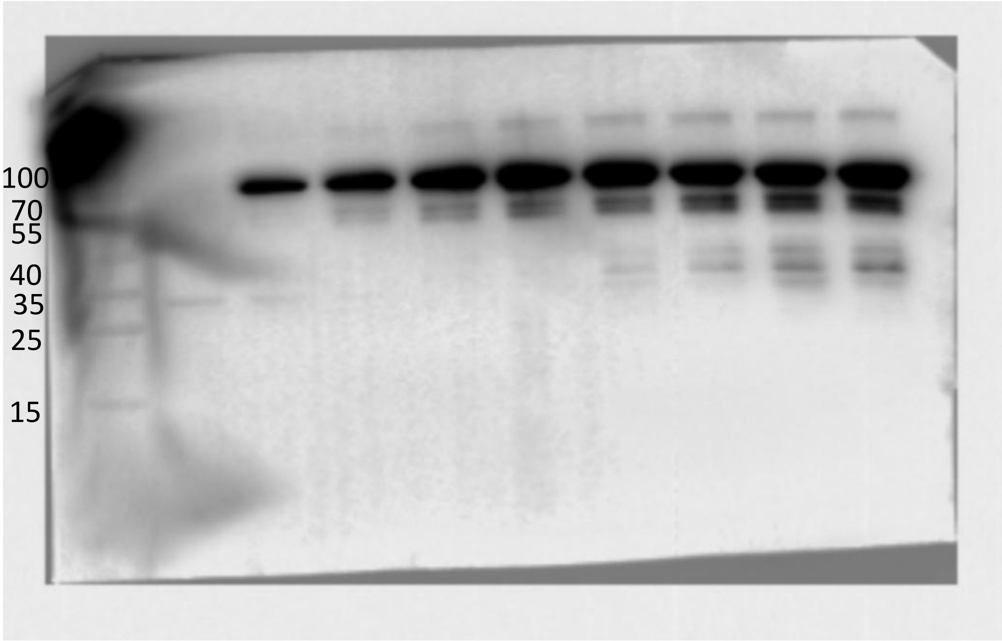

Merged with marker
Exposure time: 30 s

Figure 4B

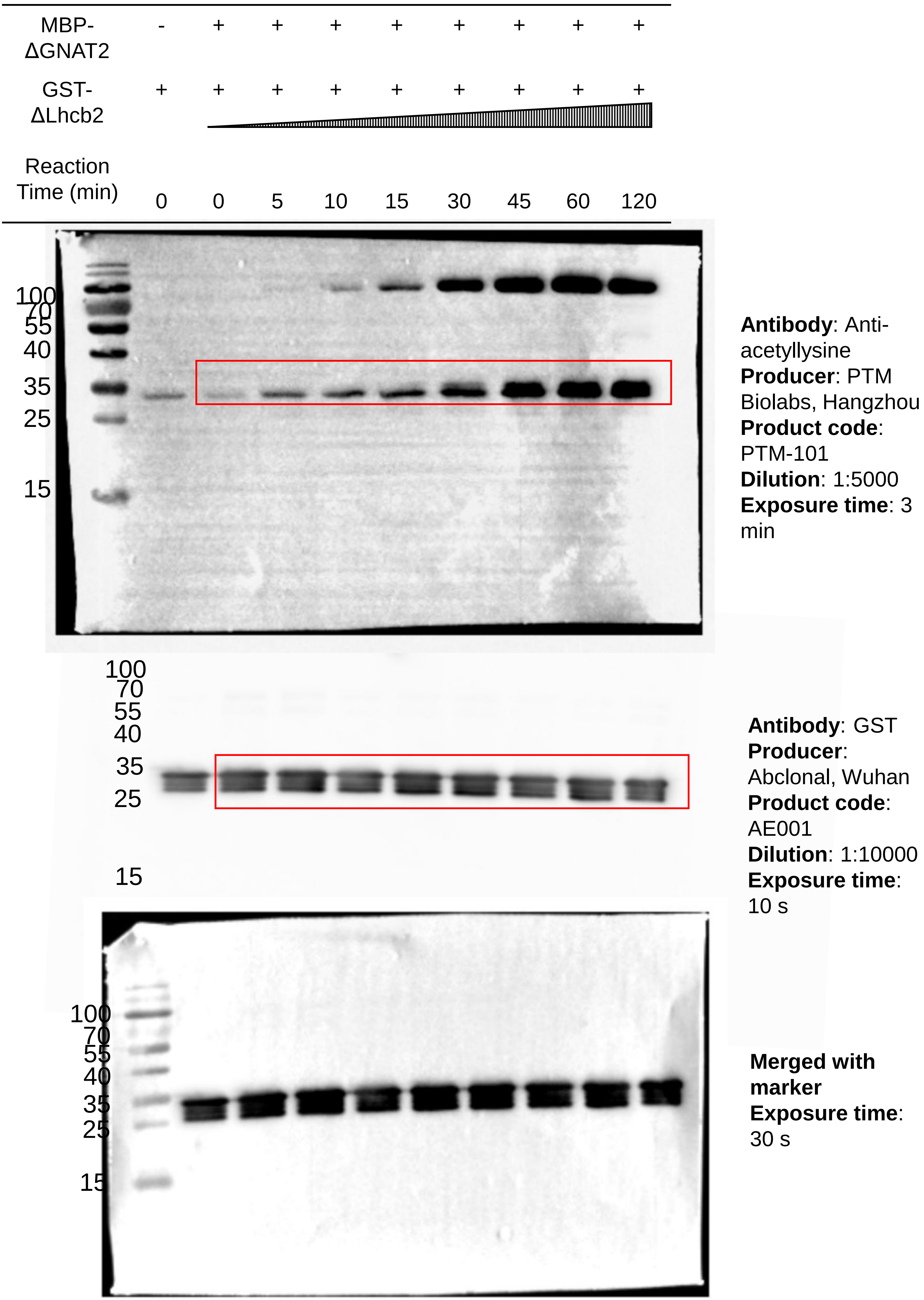

MBP-ΔGNAT2
- + + + + + + + +
GST-ΔLhcb2
+ + + + + + + + +
Reaction Time (min)
0 0 5 10 15 30 45 60 120
100
70
55
40
35
25
15
Antibody: Anti-acetyllysine
Producer: PTM Biolabs, Hangzhou
Product code: PTM-101
Dilution: 1:5000
Exposure time: 3 min
Antibody: GST
Producer: Abclonal, Wuhan
Product code: AE001
Dilution: 1:10000
Exposure time: 10 s
Merged with marker
Exposure time: 30 s

Figure 4B

MBP-ΔGNAT2	-	+	+	+	+	+	+	+	+
GST-ΔLhcb2	+	+	+	+	+	+	+	+	+
Reaction Time (min)	0	0	5	10	15	30	45	60	120

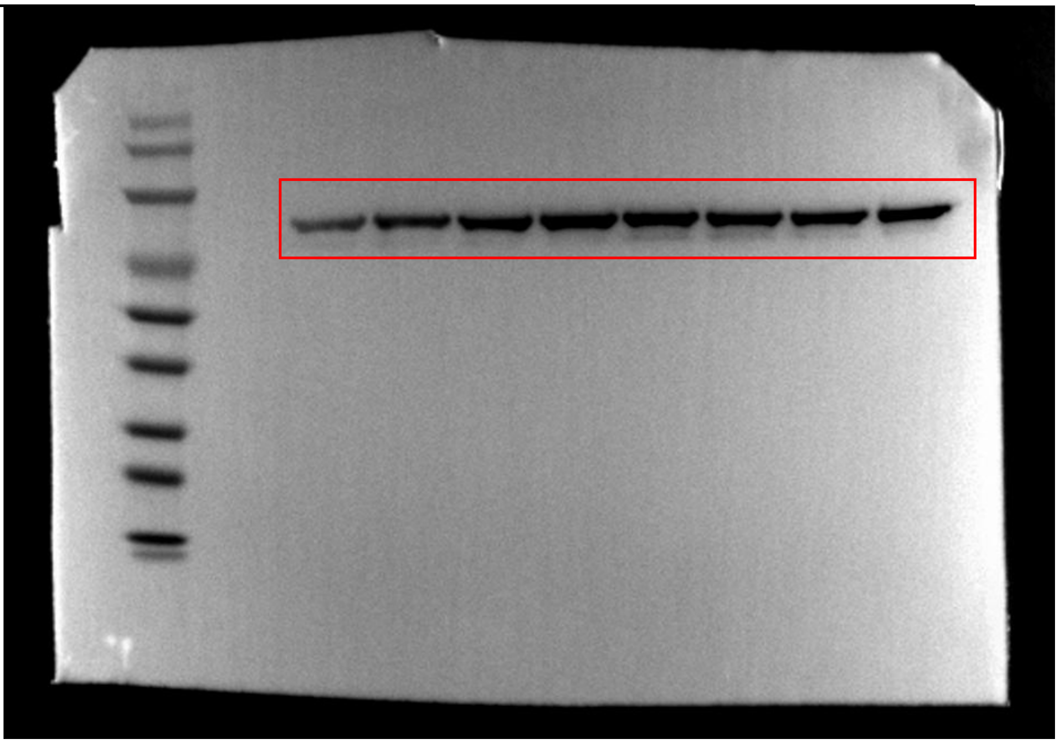

Antibody: MBP
Producer: Abclonal, Wuhan
Product code: AE016
Dilution: 1:10000
Exposure time: 10 s

Figure 4C

MBP-ΔGNAT2	-	+	+	+	+	+	+	+	+
GST-ΔLhcb2^{K20R}	+	+	+	+	+	+	+	+	+
Reaction Time (min)	0	0	5	10	15	30	45	60	120

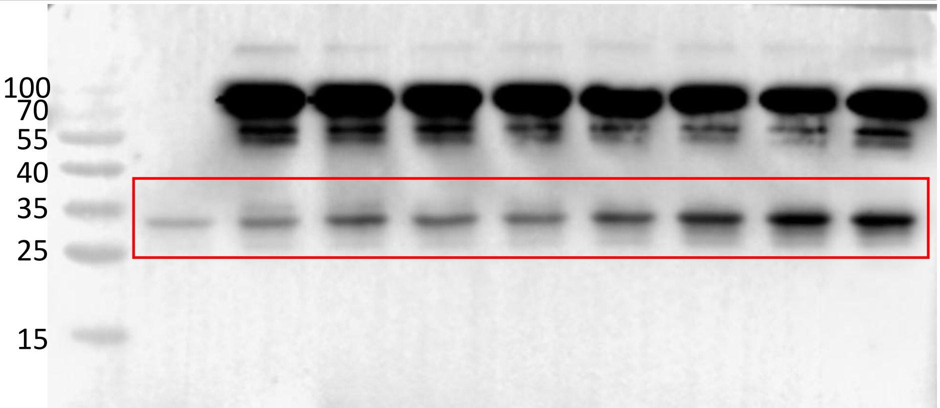

Antibody: Anti-acetyllysine
Producer: PTM Biolabs, Hangzhou
Product code: PTM-101
Dilution: 1:5000
Exposure time: 3 min

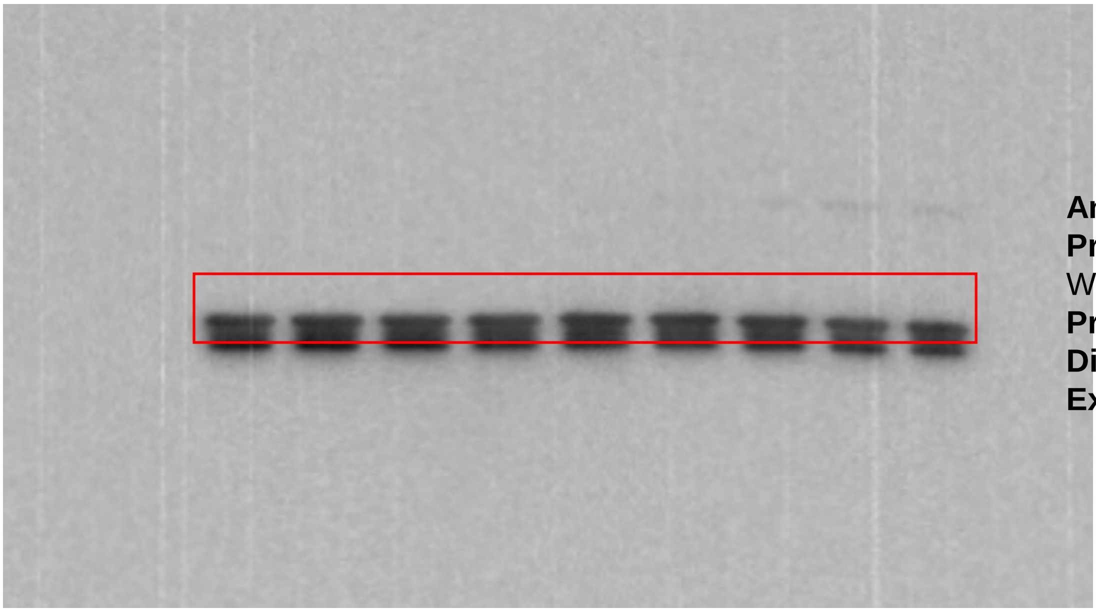

Antibody: GST
Producer: Abclonal, Wuhan
Product code: AE001
Dilution: 1:10000
Exposure time: 10 s

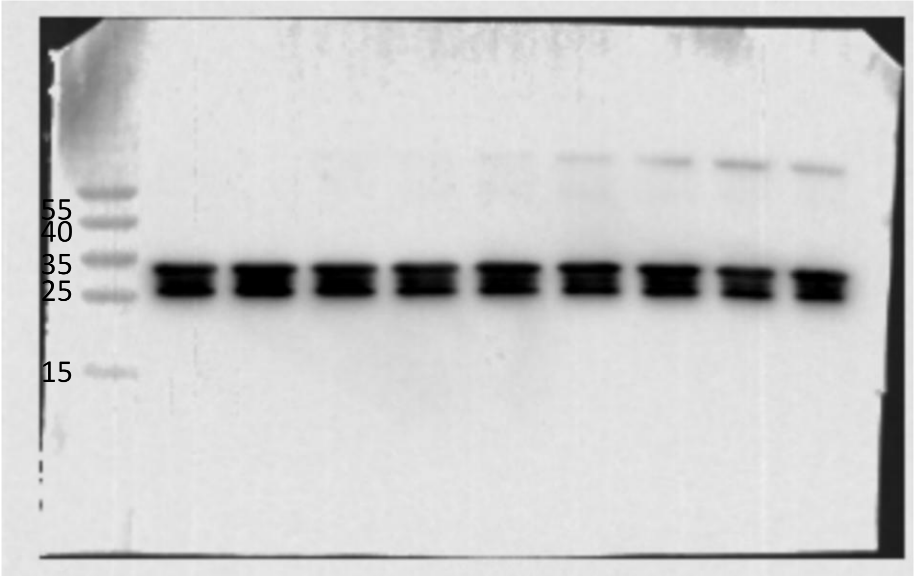

Merged with marker
Exposure time: 30 s

Figure 4C

MBP-ΔGNAT2	-	+	+	+	+	+	+	+	+
GST-ΔLhcb2^{K20R}	+	+	+	+	+	+	+	+	+
Reaction Time (min)	0	0	5	10	15	30	45	60	120

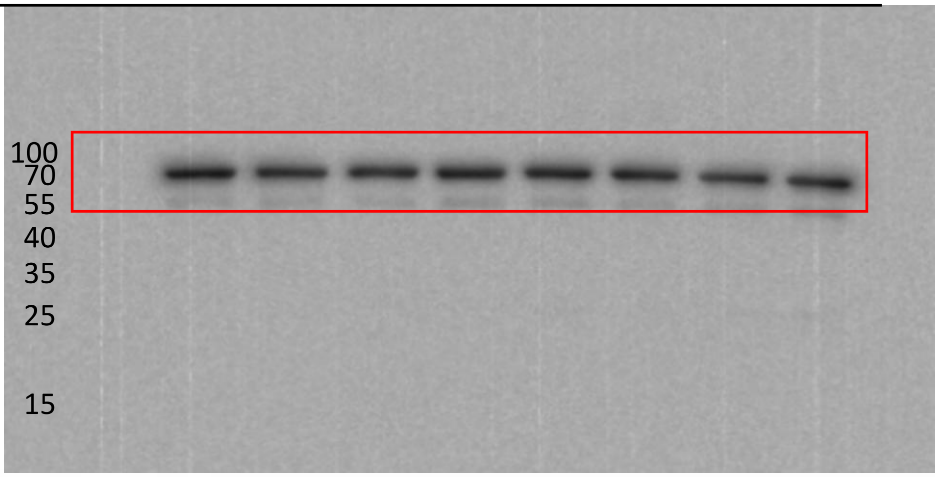

Antibody: MBP
Producer: Abclonal, Wuhan
Product code: AE016
Dilution: 1:10000
Exposure time: 10 s

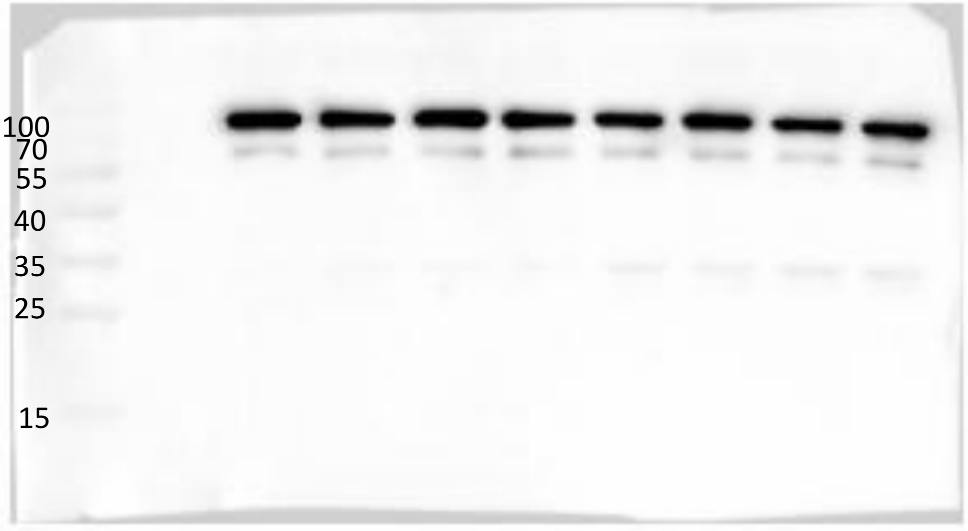

Merged with marker
Exposure time: 30 s

Figure 4D

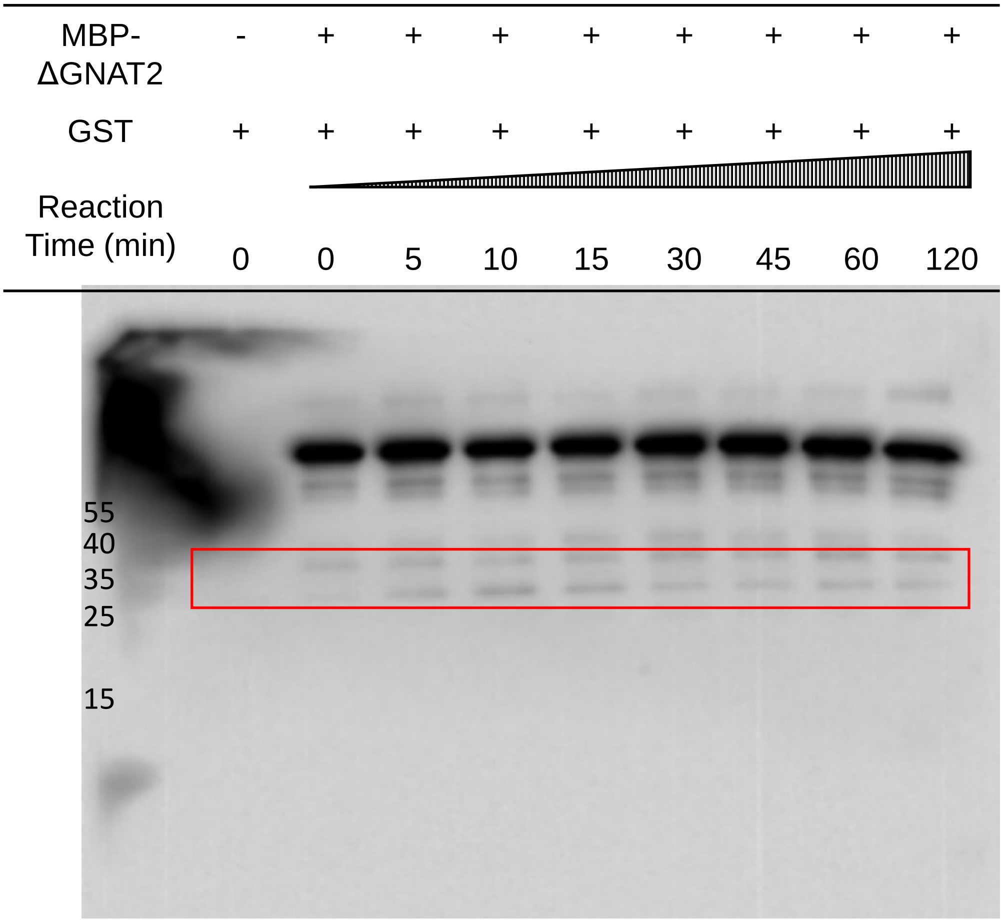

Antibody: Anti-acetyllysine
Producer: PTM Biolabs, Hangzhou
Product code: PTM-101
Dilution: 1:5000
Exposure time: 1 min

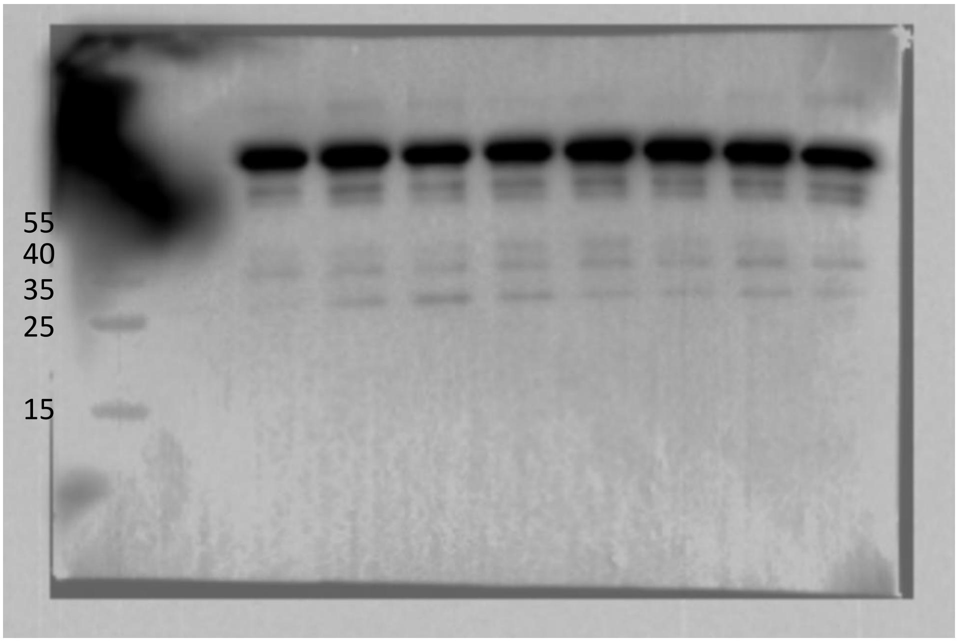

Merged with marker

Figure 4D

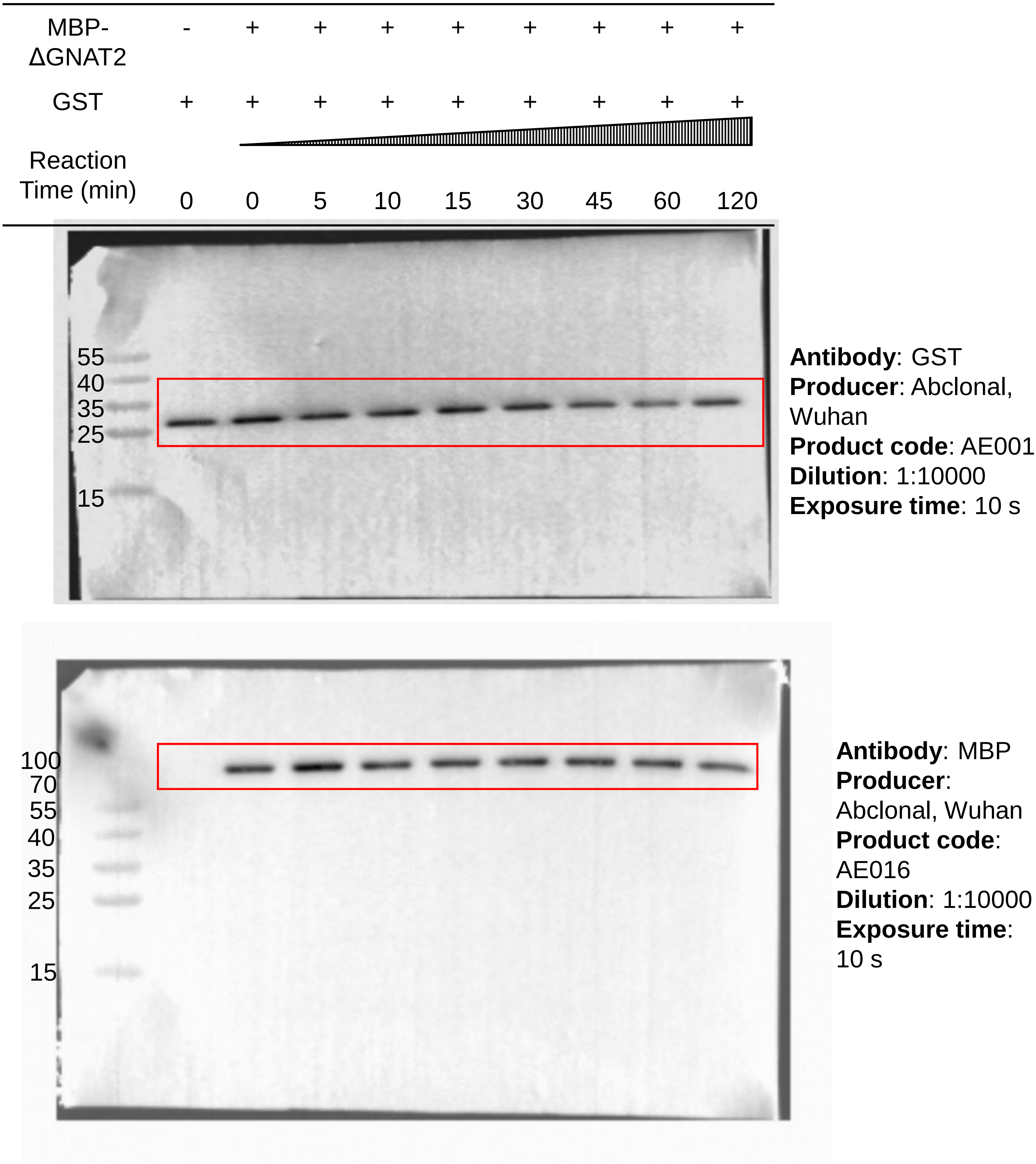
MBP-ΔGNAT2 - + + + + + + + +
GST + + + + + + + + +
Reaction Time (min) 0 0 5 10 15 30 45 60 120
55
40
35
25
15
Antibody: GST
Producer: Abclonal, Wuhan
Product code: AE001
Dilution: 1:10000
Exposure time: 10 s
100
70
55
40
35
25
15
Antibody: MBP
Producer: Abclonal, Wuhan
Product code: AE016
Dilution: 1:10000
Exposure time: 10 s

Figure 4E

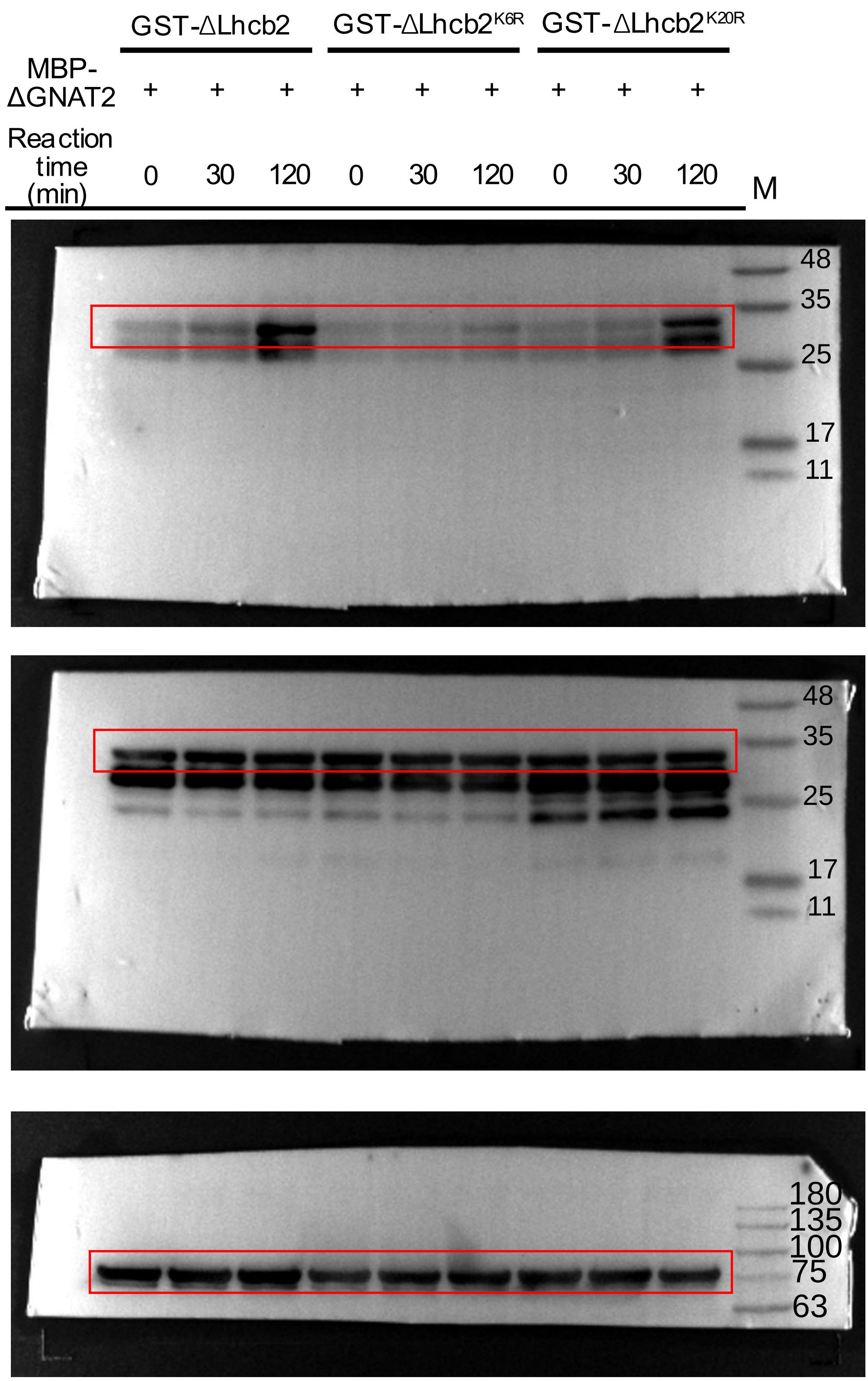

Antibody: Anti-acetyllysine
Producer: PTM Biolabs, Hangzhou
Product code: PTM-101
Dilution: 1:5000
Exposure time: 30 s
Marker: ColorMixed Protein Marker (11-180 kD), Solarbio, PR1910

Antibody: GST
Producer: TransGen Biotech, Beijing
Product code: HT601-01
Dilution: 1:10000
Exposure time: 2 s
Marker: ColorMixed Protein Marker (11-180 kD), Solarbio, PR1910

Antibody: MBP
Producer: Abmart, Shanghai
Product code: M20051
Dilution: 1:10000
Exposure time: 2 s
Marker: ColorMixed Protein Marker (11-180 kD), Solarbio, PR1910

Figure 4F

MBP-ΔGNAT2	-	+	+	+	+	-	-	-	-
MBP-ΔGNAT2^{C131A}	-	-	-	-	-	+	+	+	+
GST-ΔLhcb2	+	+	+	+	+	+	+	+	+
Reaction Time (min)	0	0	30	60	120	0	30	60	120

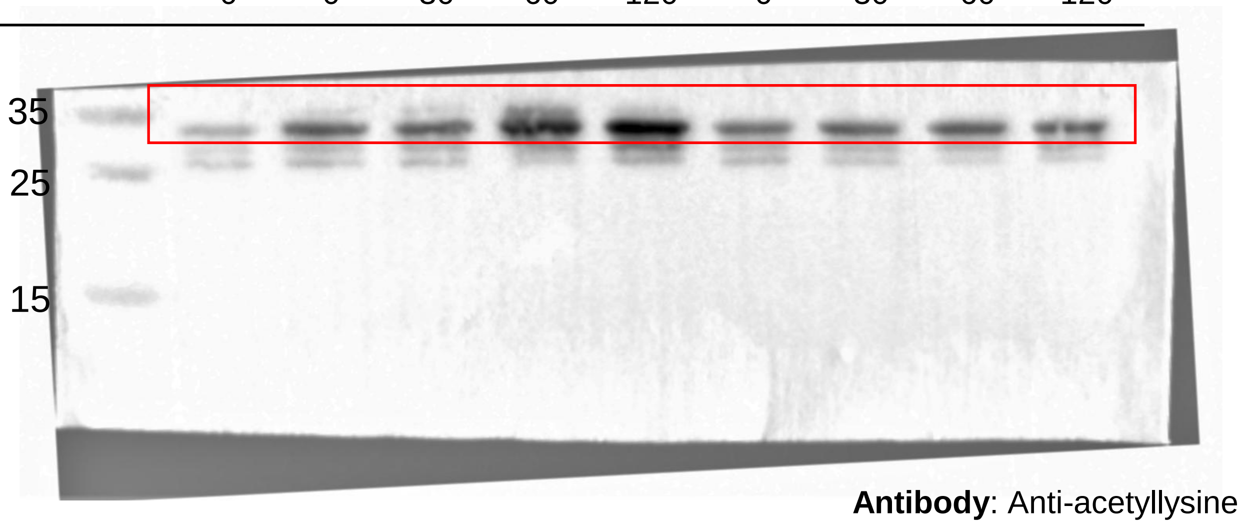

Antibody: Anti-acetyllysine
Producer: PTM Biolabs, Hangzhou
Product code: PTM-101
Dilution: 1:5000
Exposure time: 3 min

MBP-ΔGNAT2	-	+	+	+	+	-	-	-	-
MBP-ΔGNAT2^{C131A}	-	-	-	-	-	+	+	+	+
GST-ΔLhcb2	+	+	+	+	+	+	+	+	+
Reaction Time (min)	0	0	30	60	120	0	30	60	120

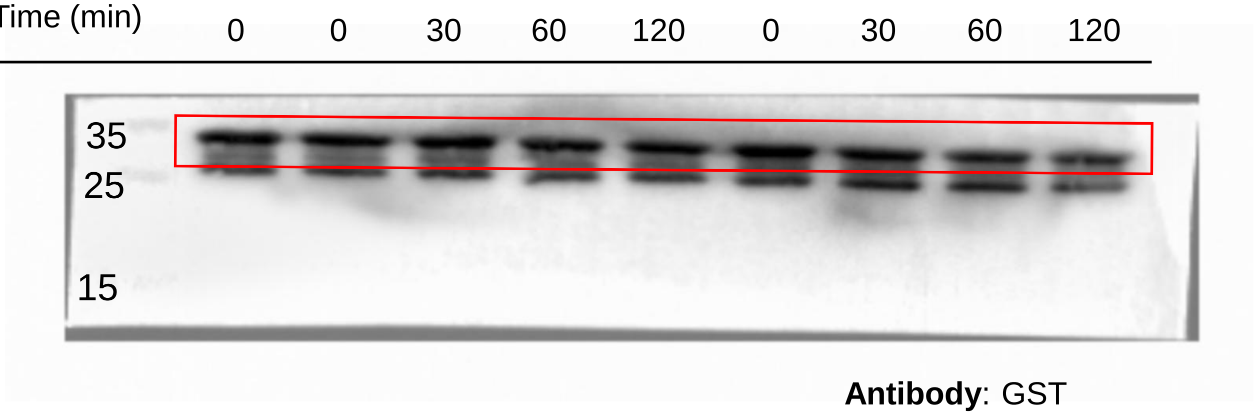

Antibody: GST
Producer: Abclonal, Wuhan
Product code: AE001
Dilution: 1:10000
Exposure time: 10 s

Figure 4G

		MBP-ΔGNAT2 Redox treatment				MBP-ΔGNAT2^{C131A} Redox treatment				MBP Redox treatment			
O_2 (min)		0	20	20	20	0	20	20	20	0	20	20	20
TCEP (mM)		0	0	1	100	0	0	1	100	0	0	1	100
MBP-ΔGNAT2	-	+	+	+	+	-	-	-	-	-	-	-	-
MBP-ΔGNAT2^{C131A}	-	-	-	-	-	+	+	+	+	-	-	-	-
MBP	-	-	-	-	-	-	-	-	-	+	+	+	+
GST-ΔLhcb2	+	+	+	+	+	+	+	+	+	+	+	+	+
Reaction Time (min)	0	30	30	30	30	30	30	30	30	30	30	30	30

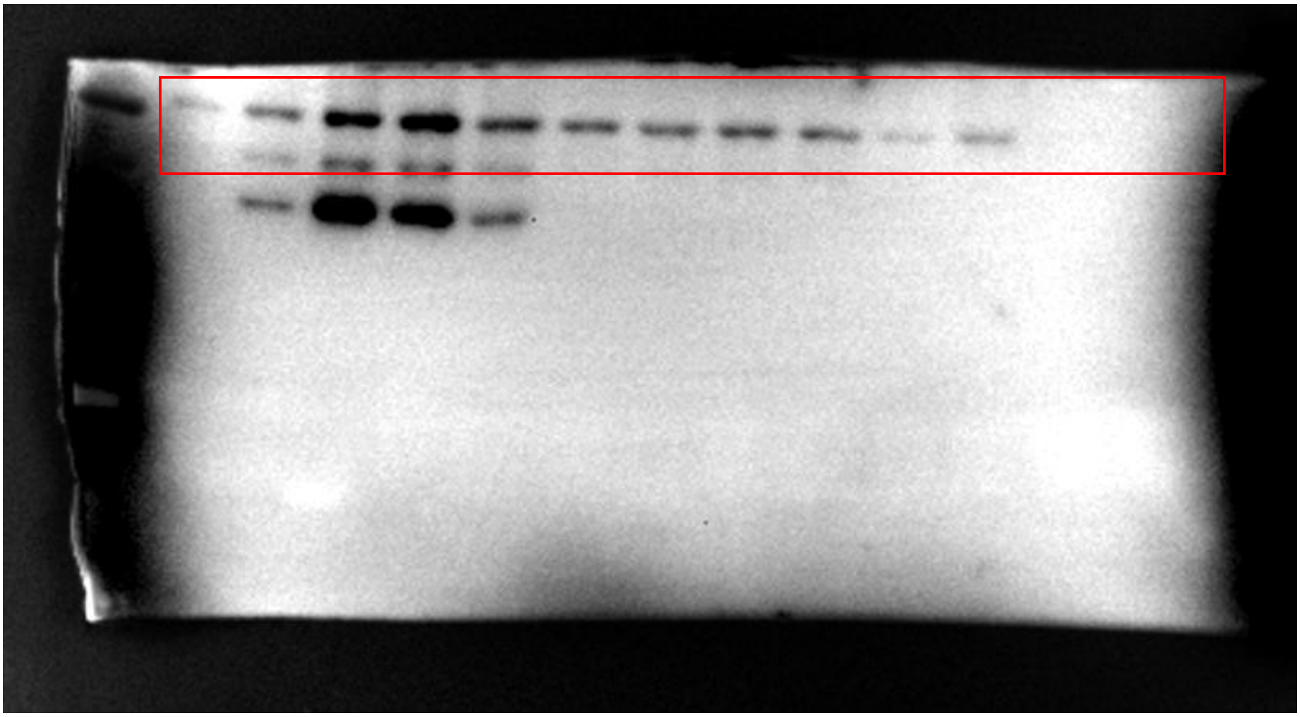

Antibody: Anti-acetyllysine
Producer: PTM Biolabs, Hangzhou
Product code: PTM-101
Dilution: 1:5000
Exposure time: 3 min
Marker:180 kDa Prestained Protein Marker, Vazyme, MP102-01

Figure 4G

		MBP-ΔGNAT2 Redox treatment				MBP-ΔGNAT2^{C131A} Redox treatment				MBP Redox treatment			
O_2 (min)		0	20	20	20	0	20	20	20	0	20	20	20
TCEP (mM)		0	0	1	100	0	0	1	100	0	0	1	100
MBP-ΔGNAT2	-	+	+	+	+	-	-	-	-	-	-	-	-
MBP-ΔGNAT2^{C131A}	-	-	-	-	-	+	+	+	+	-	-	-	-
MBP	-	-	-	-	-	-	-	-	-	+	+	+	+
GST-ΔLhcb2	+	+	+	+	+	+	+	+	+	+	+	+	+
Reaction Time (min)	0	30	30	30	30	30	30	30	30	30	30	30	30

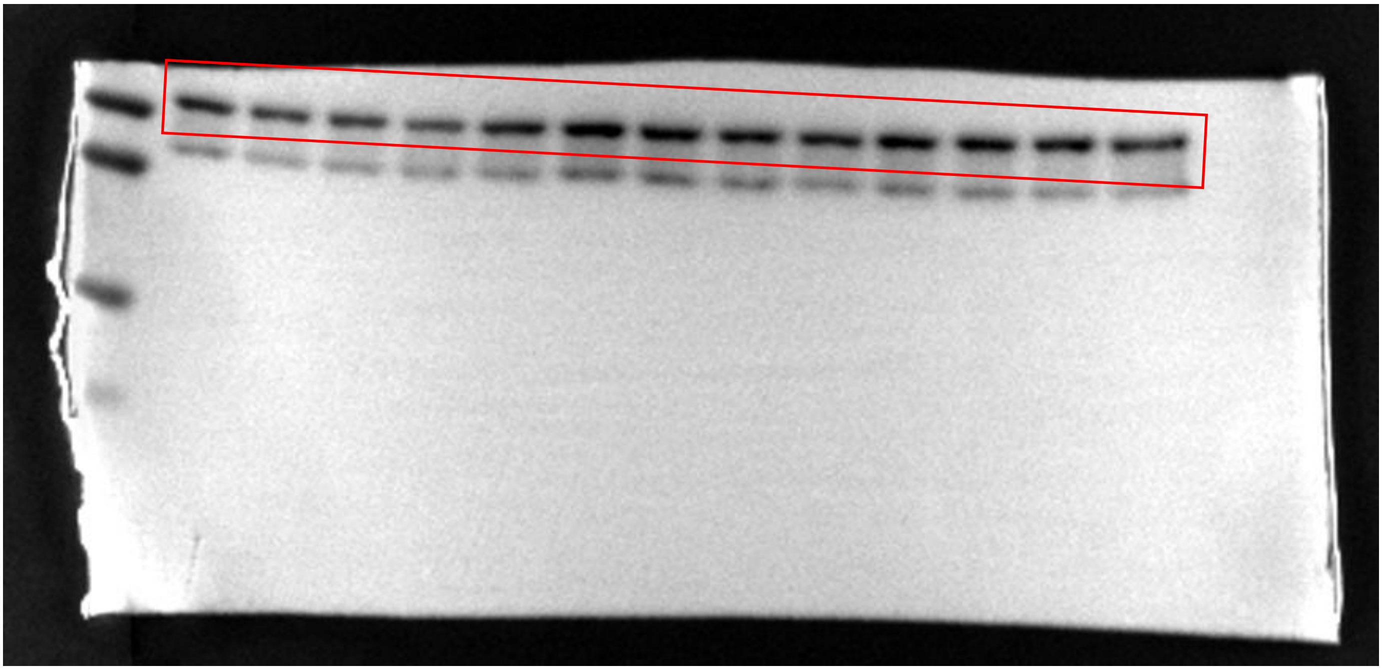

Antibody: GST
Producer: Abclonal, Wuhan
Product code: AE001
Dilution: 1:10000
Exposure time: 10 s
Marker:180 kDa Prestained Protein Marker, Vazyme, MP102-01

Supplemental Figure 5F

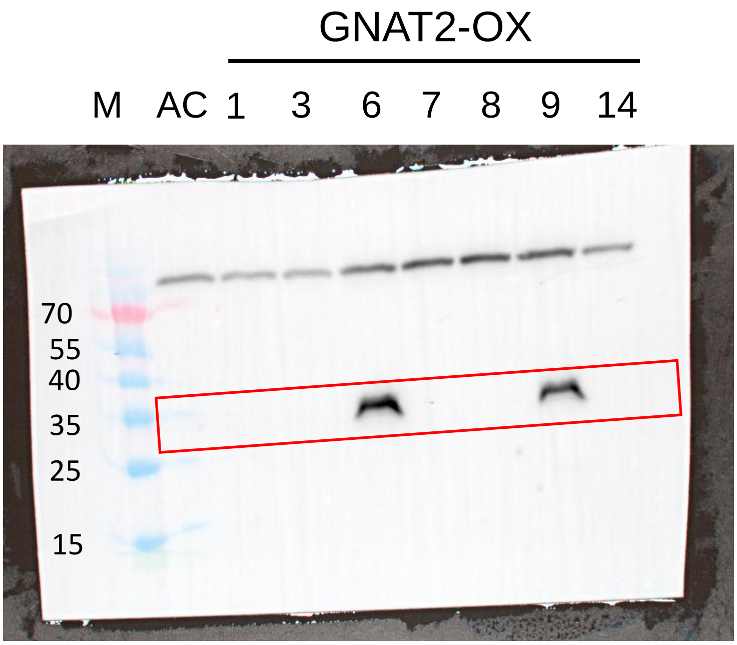

Antibody: Monoclonal ANTI-FLAG® M2-Peroxidase (HRP)
Producer: Sigma-Aldrich
Product code:A8592
Dilution:1:10000
Exposure time:1 min

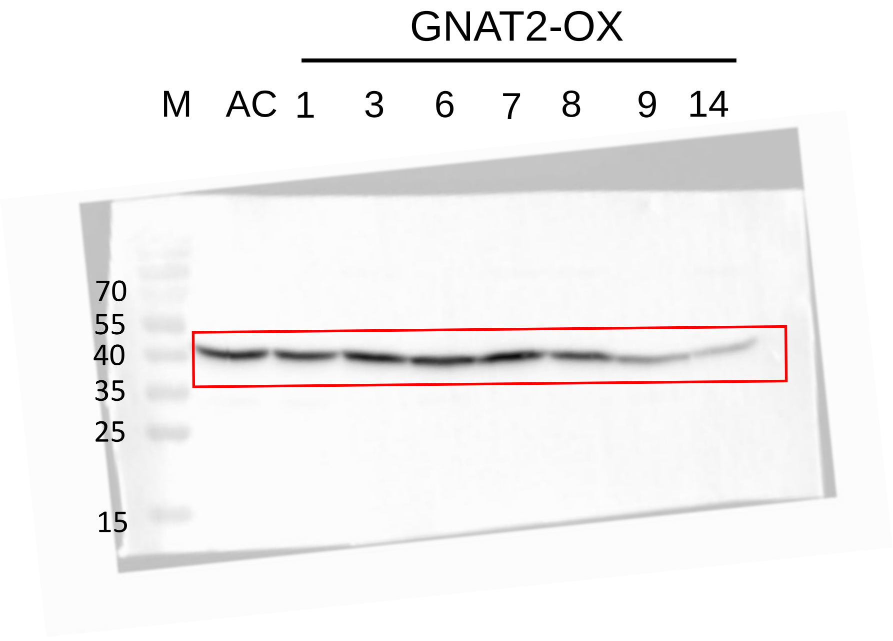

Antibody: Actin
Producer: Abclonal, Wuhan
Product code:AC009
Dilution:1:10000
Exposure time:10 s

Supplemental Figure 6B

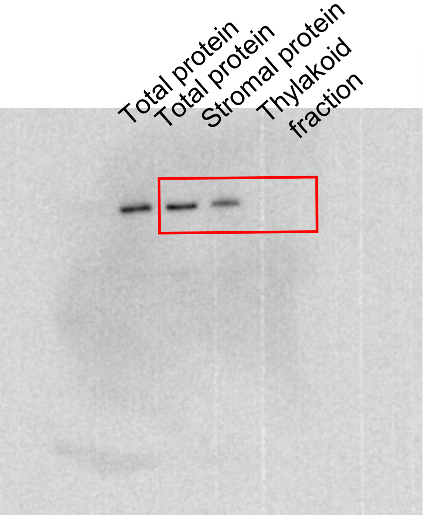

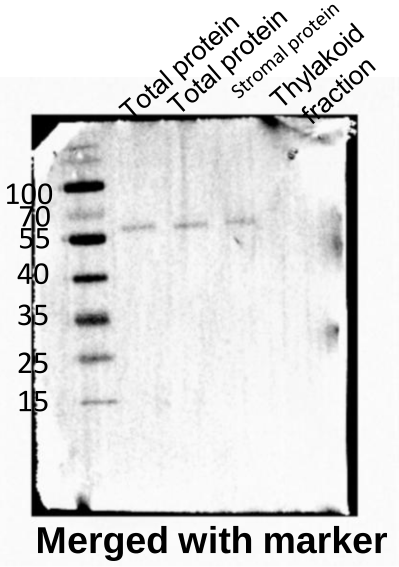

Antibody: GFP
Producer: Abclonal
Product code:AE012
Dilution:1:10000
Exposure time: Left-5 min /Right-1 min

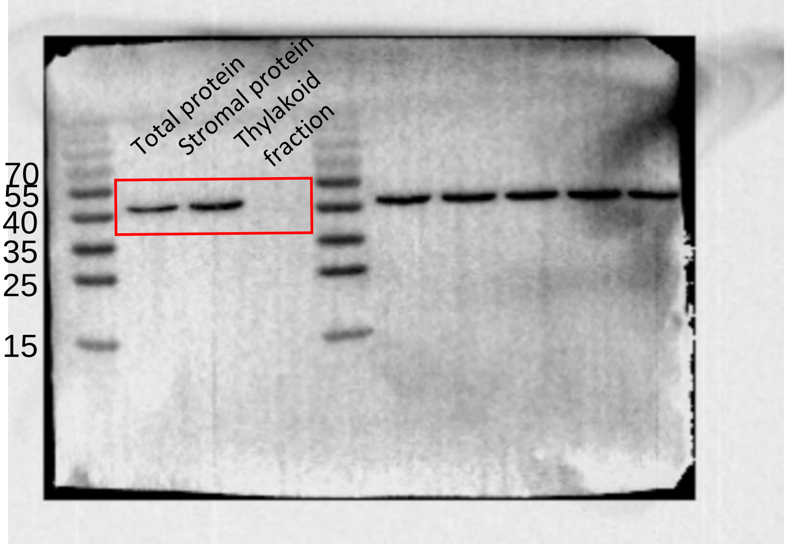

Antibody: Actin
Producer: Abclonal
Product code: AC009
Dilution: 1:10000
Exposure time: 5 s

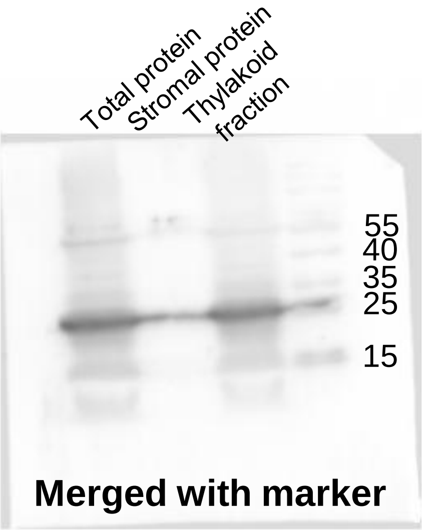

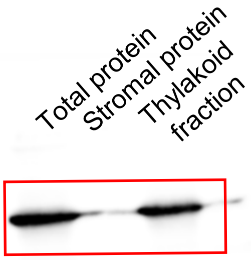

Antibody: LHCII antibody
Producer: Obtained from Prof. Lixin Zhang
Dilution:1:10000
Secondary antibody: HRP Goat Anti-Rabbit IgG, ABclonal, AS014, 1:10000 dilution
Exposure time: Left-2 s/Right-5 s

Supplemental Figure 10A

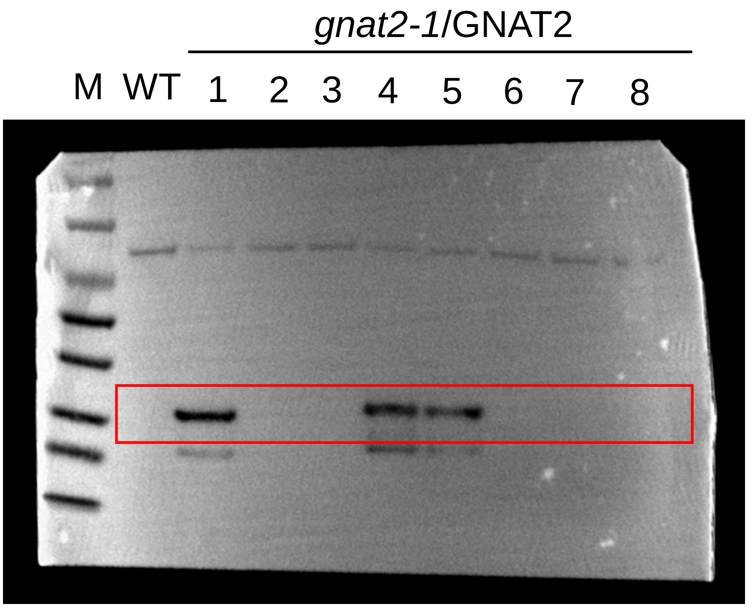

Antibody: Monoclonal ANTI-FLAG® M2-Peroxidase (HRP)
Producer: Sigma-Aldrich
Product code:A8592
Dilution:1:10000
Exposure time:1 min

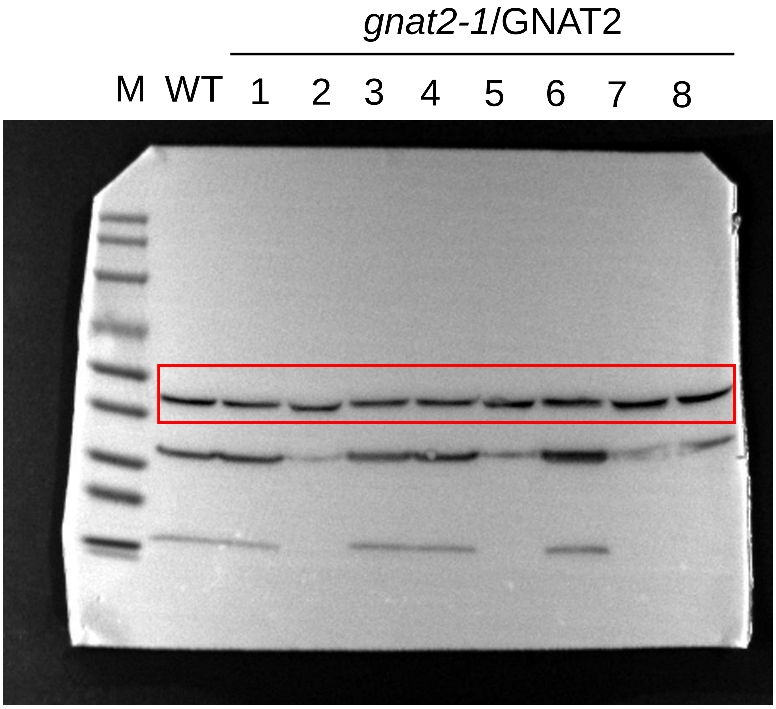

Antibody: Actin
Producer: Abclonal, Wuhan
Product code:AC009
Dilution:1:10000
Exposure time:10 s

Supplemental Figure 10B

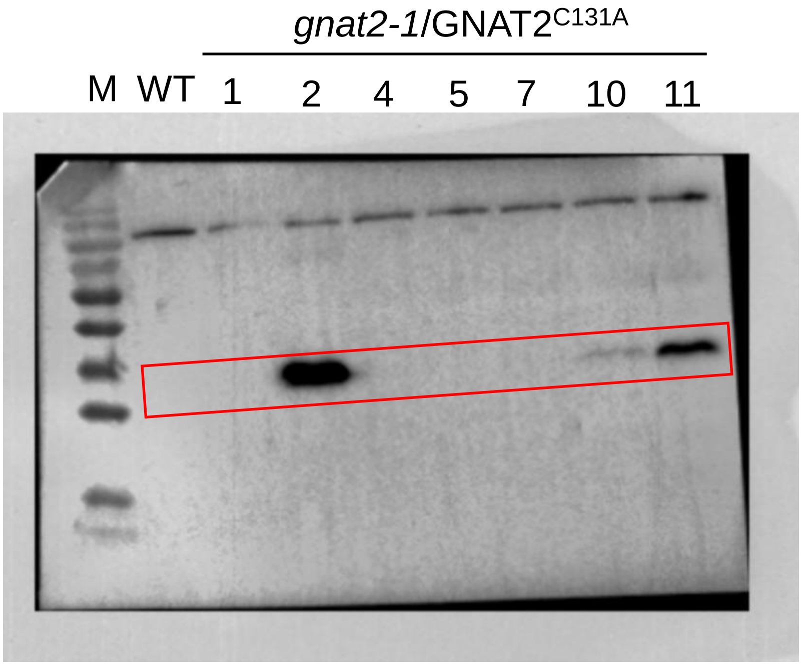

Antibody: Monoclonal ANTI-FLAG® M2-Peroxidase (HRP)
Producer: Sigma-Aldrich
Product code:A8592
Dilution:1:10000
Exposure time:1 min

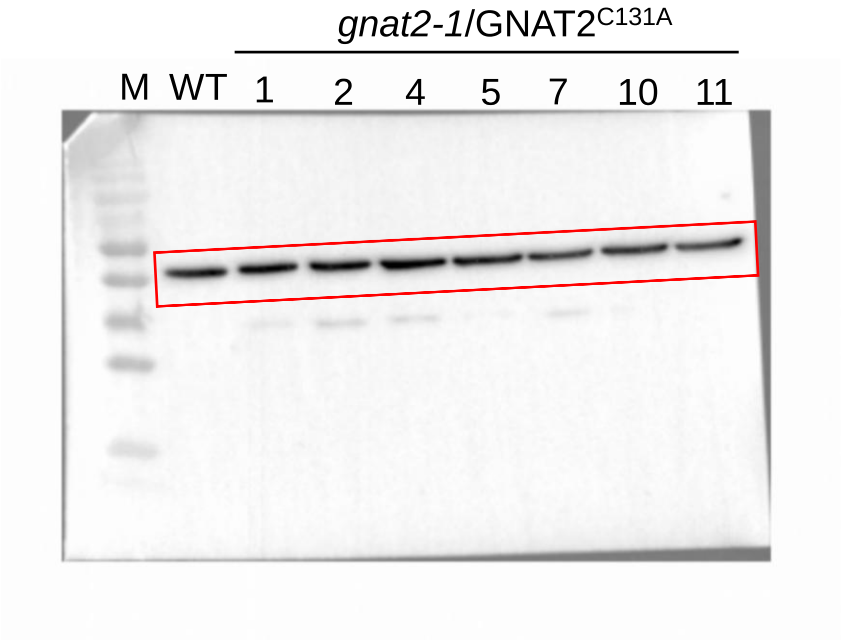

Antibody: Actin
Producer: Abclonal, Wuhan
Product code:AC009
Dilution:1:10000
Exposure time:10 s

Supplemental Figure 10C

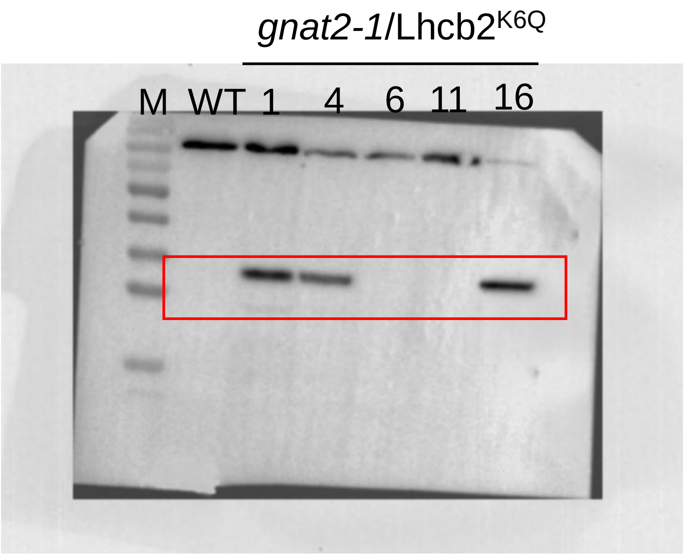

Antibody: Monoclonal ANTI-FLAG® M2-Peroxidase (HRP)
Producer: Sigma-Aldrich
Product code:A8592
Dilution:1:10000
Exposure time:1 min

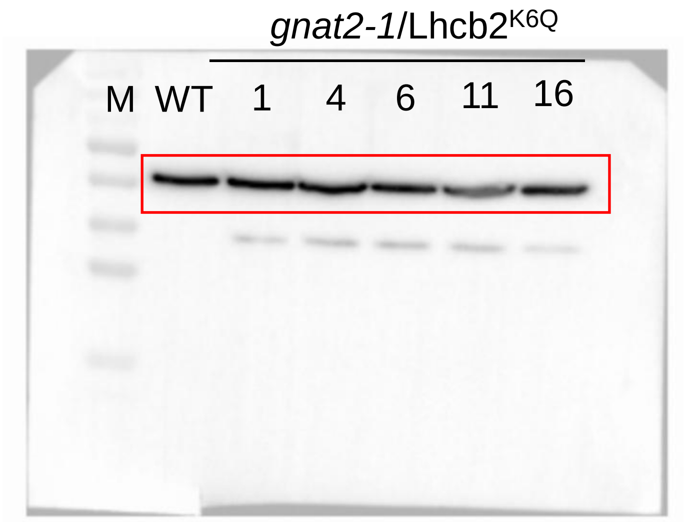

Antibody: Actin
Producer: Abclonal, Wuhan
Product code:AC009
Dilution:1:10000
Exposure time:10 s

Supplemental Figure 10D

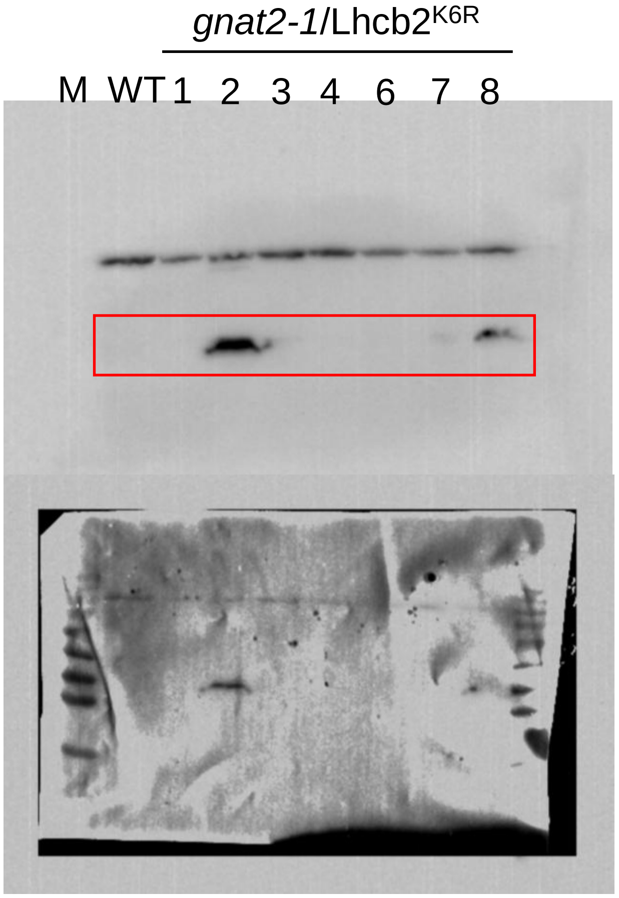

Antibody: Monoclonal ANTI-FLAG® M2-Peroxidase (HRP)
Producer: Sigma-Aldrich
Product code:A8592
Dilution:1:10000
Exposure time:1 min

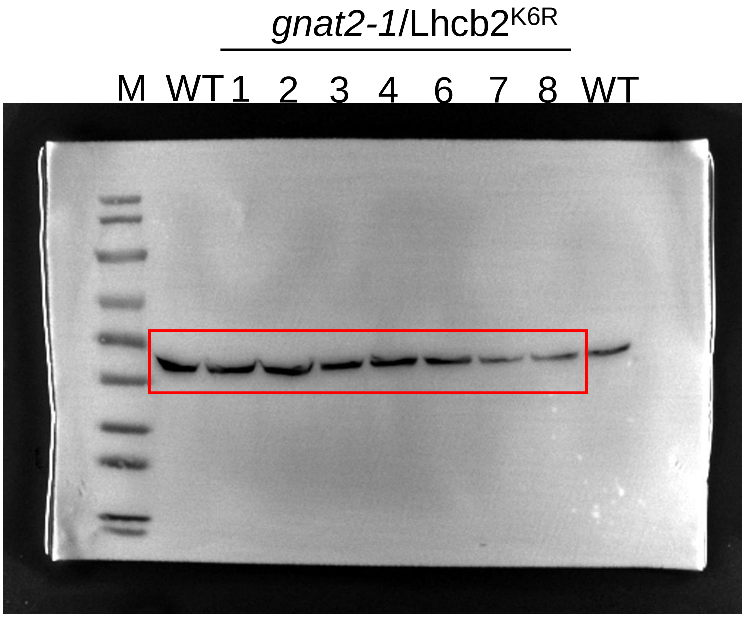

Antibody: Actin
Producer: Abclonal, Wuhan
Product code:AC009
Dilution:1:10000
Exposure time:10 s

Supplemental Figure 11E

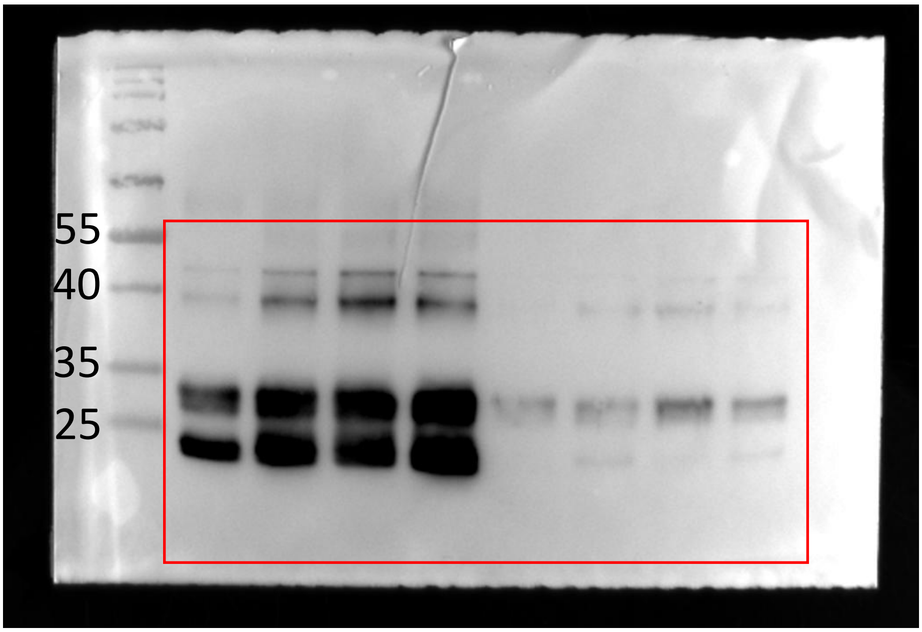

Antibody: Phospho Threonine/Tyrosine
Producer: Cell Signaling Technology
Product code: 9381S
Dilution:1:10000
Exposure time:1 min

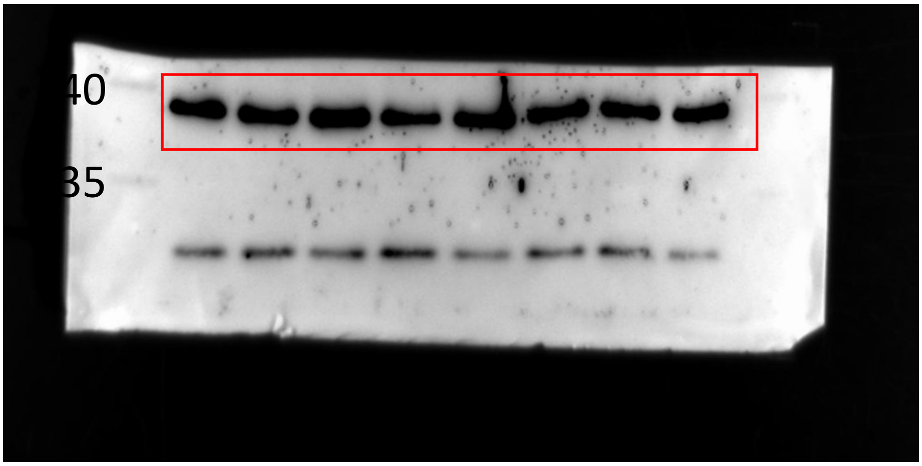

Antibody: Actin
Producer: Abclonal, Wuhan
Product code:AC009
Dilution:1:10000
Exposure time:10 s

Supplemental Figure 12

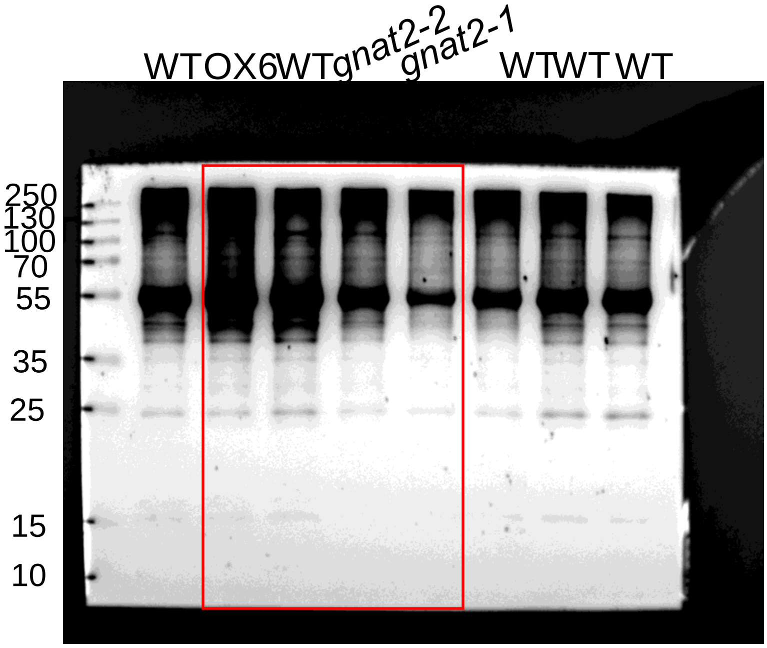

Antibody: Anti-acetyllysine
Producer: PTM Biolabs, Hangzhou
Product code: PTM-101
Dilution: 1:1000
Exposure time: 1 min

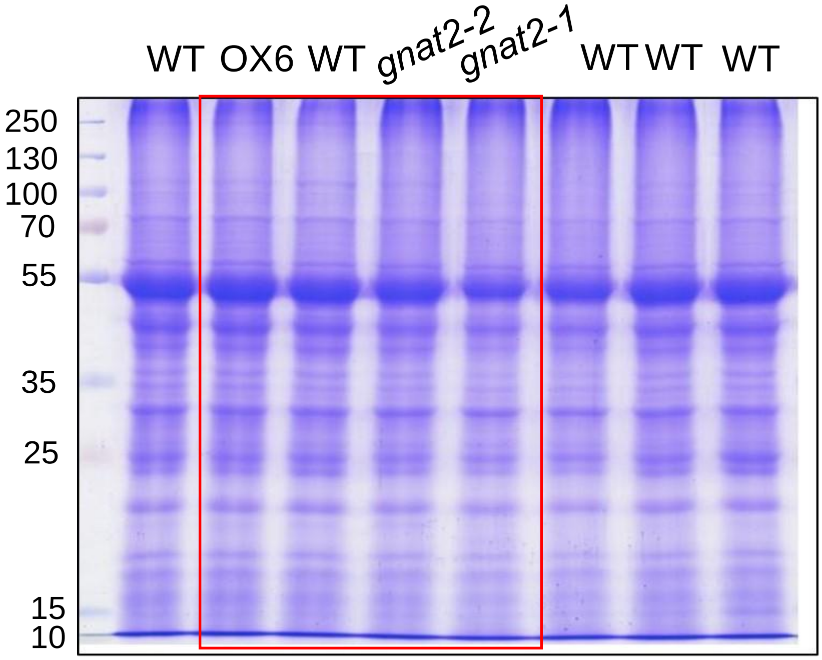

Supplemental Figure 15A

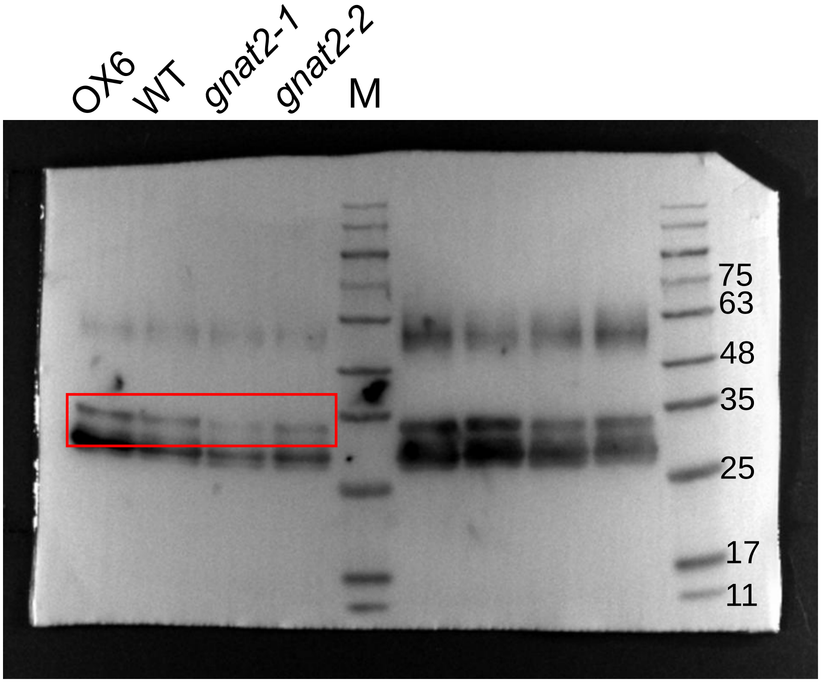

Antibody: Anti-acetyllysine
Producer: PTM Biolabs, Hangzhou
Product code: PTM-101
Dilution: 1:5000
Exposure time: 1 min
Marker: ColorMixed Protein Marker (11-180 kD), Solarbio, PR1910

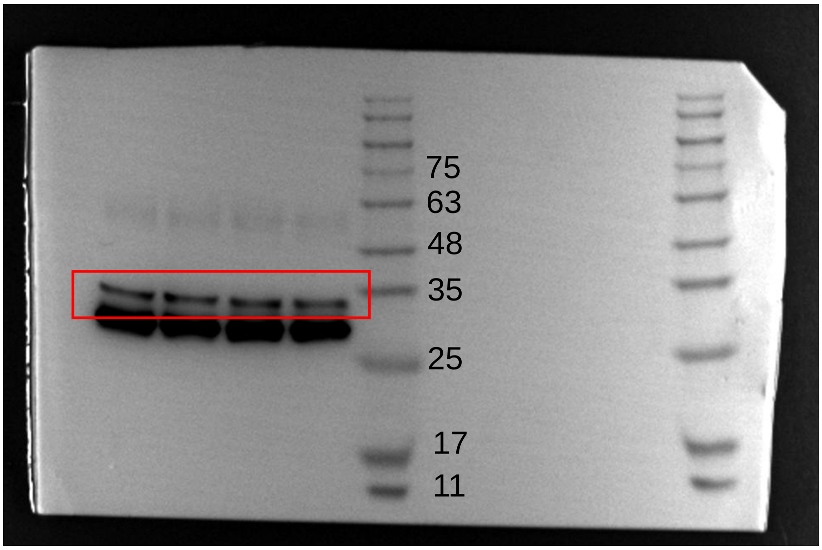

Antibody: GST
Producer: TransGen Biotech, Beijing
Product code: HT601-01
Dilution: 1:10000
Exposure time: 2.5 s
Marker: ColorMixed Protein Marker (11-180 kD), Solarbio, PR1910

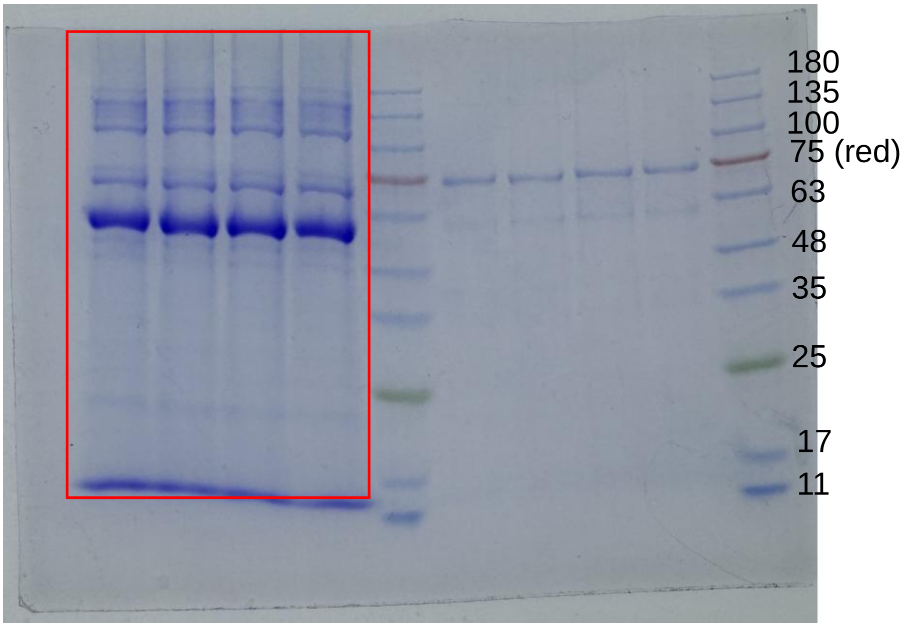

Supplemental Figure 15B

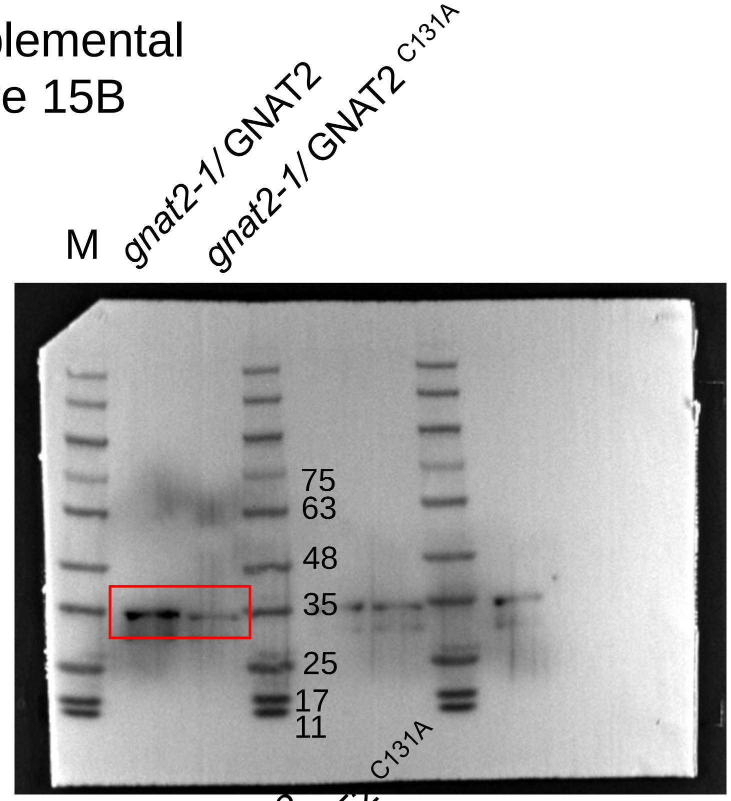

Antibody: Anti-acetyllysine
Producer: PTM Biolabs, Hangzhou
Product code: PTM-101
Dilution: 1:5000
Exposure time: 1 min
Marker: ColorMixed Protein Marker (11-180 kD), Solarbio, PR1910

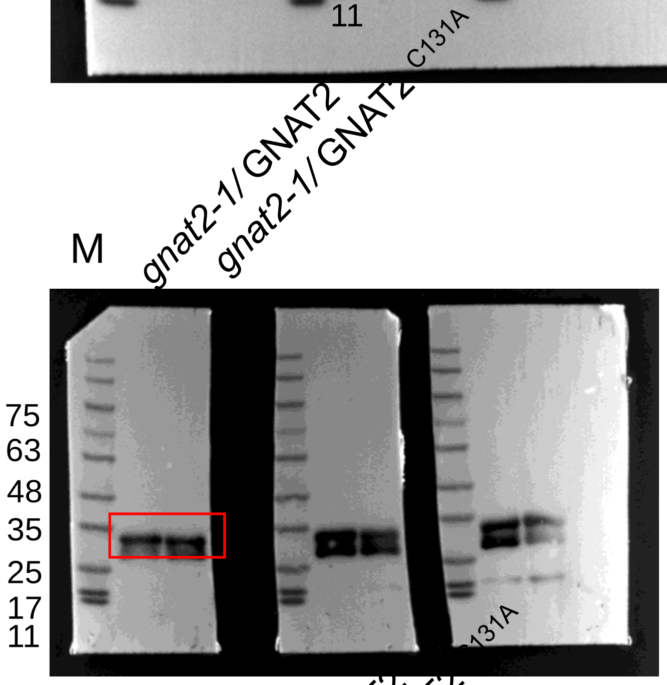

Antibody: GST
Producer: TransGen Biotech, Beijing
Product code: HT601-01
Dilution: 1:10000
Exposure time: 2.5 s
Marker: ColorMixed Protein Marker (11-180 kD), Solarbio, PR1910

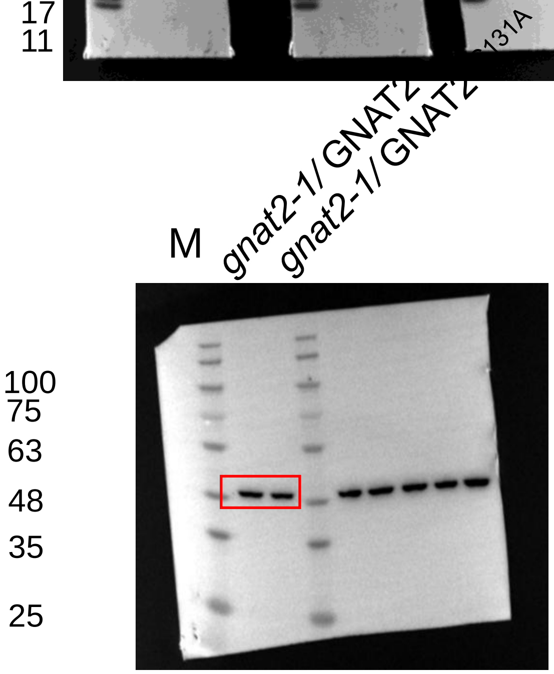

Antibody: Actin
Producer: Abclonal, Wuhan
Product code:AC009
Dilution:1:10000
Exposure time:10 s

Supplemental Figure 16

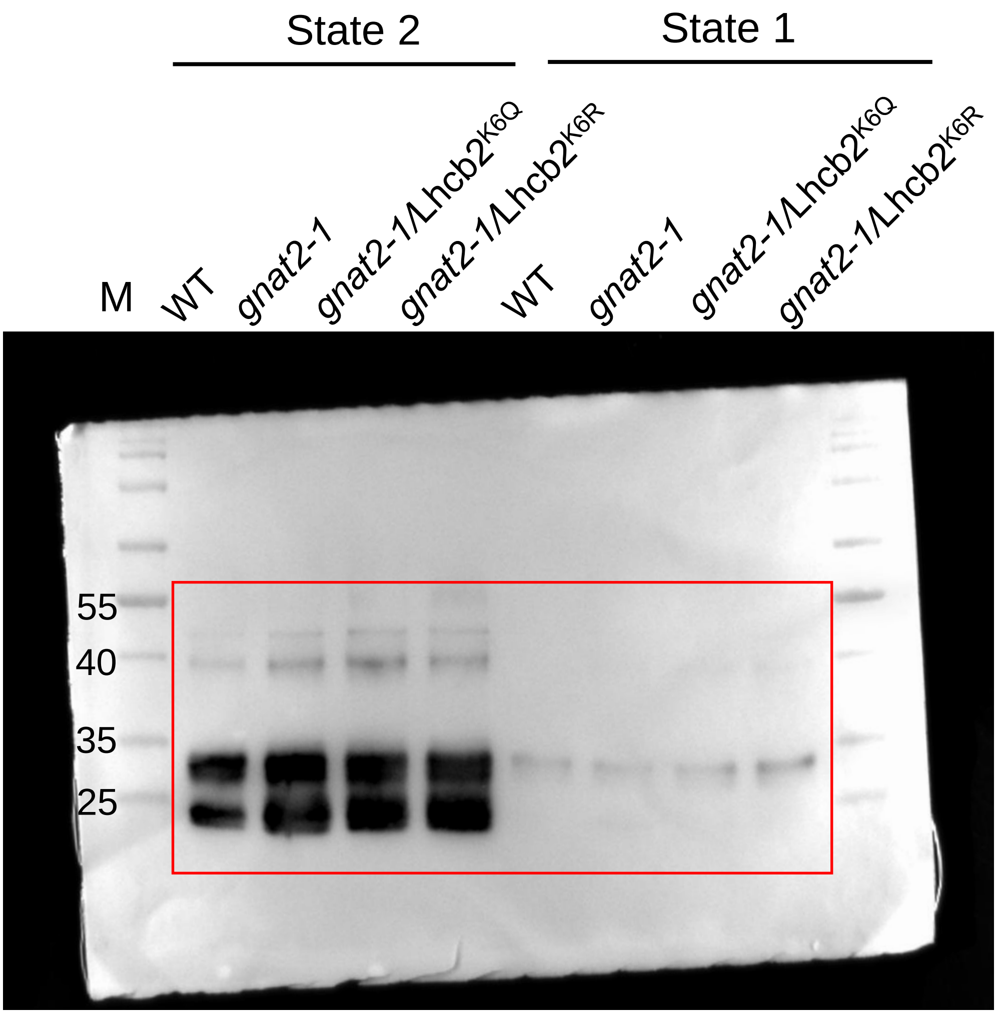

Antibody: Phospho Threonine/Tyrosine
Producer: Cell Signaling Technology
Product code: 9381S
Dilution:1:10000
Exposure time:1 min

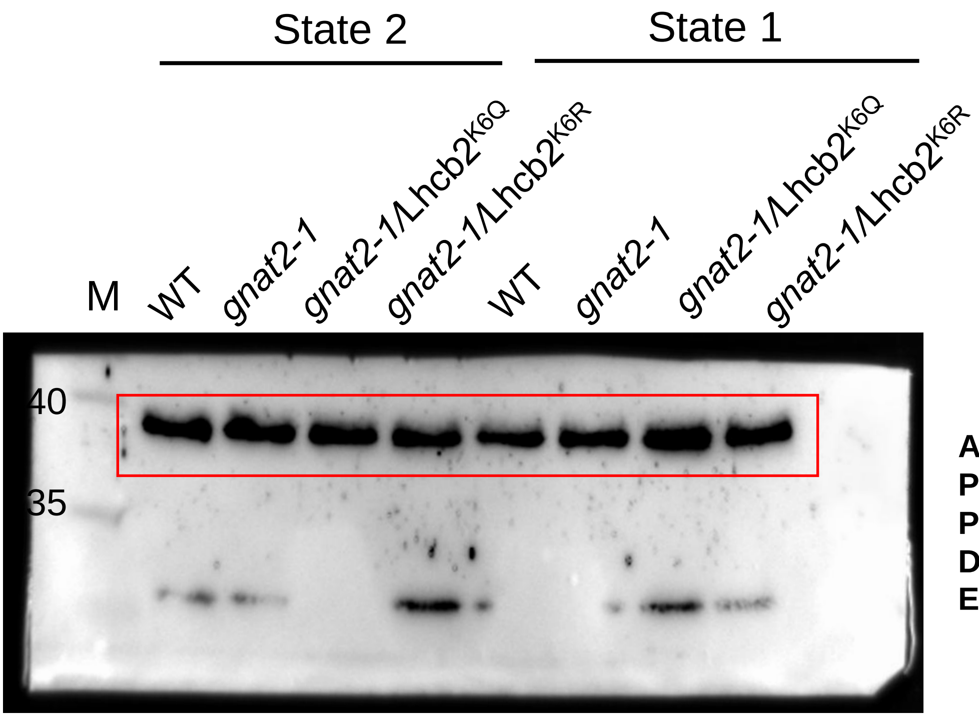

Antibody: Actin
Producer: Abclonal, Wuhan
Product code:AC009
Dilution:1:10000
Exposure time:10 s
