# Supplementary material for: Chloroplast acetyltransferase GNAT2 acts as a redox-regulated switch for state transitions in tomato
Source: Mol Hortic. 2025 Aug 6;5:39. doi: 10.1186/s43897-025-00164-0 (PMC12326663; doi:10.1186/s43897-025-00164-0)

# Supplemental Data set 3-Related MS_MS spectra of acetylated peptides

**A. Spectra of related acetylated peptides of photosynthetic proteins in acetylome data.**

| Protein accession | Protein name | Protein ID | Modified sequence | Position | Charge | Mass error [ppm] | MS/MS Count |
|---|---|---|---|---|---|---|---|
| A0A3Q7EIF5 | PsbP | Solyc01g087040 | LVSK(1)VEK | 115 | 3 | 0.0607 | 1 |

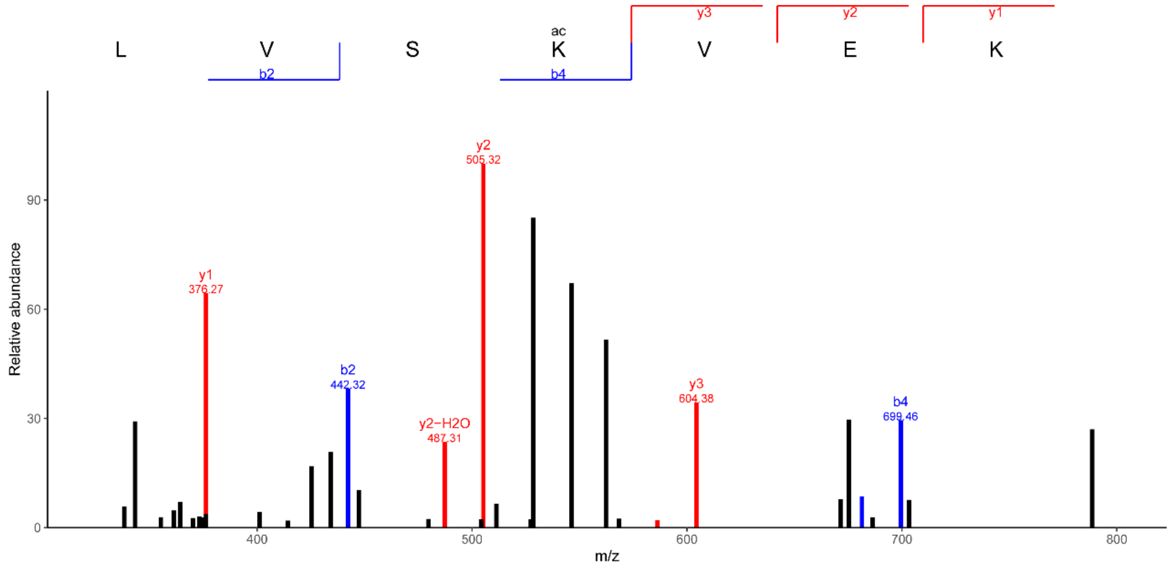


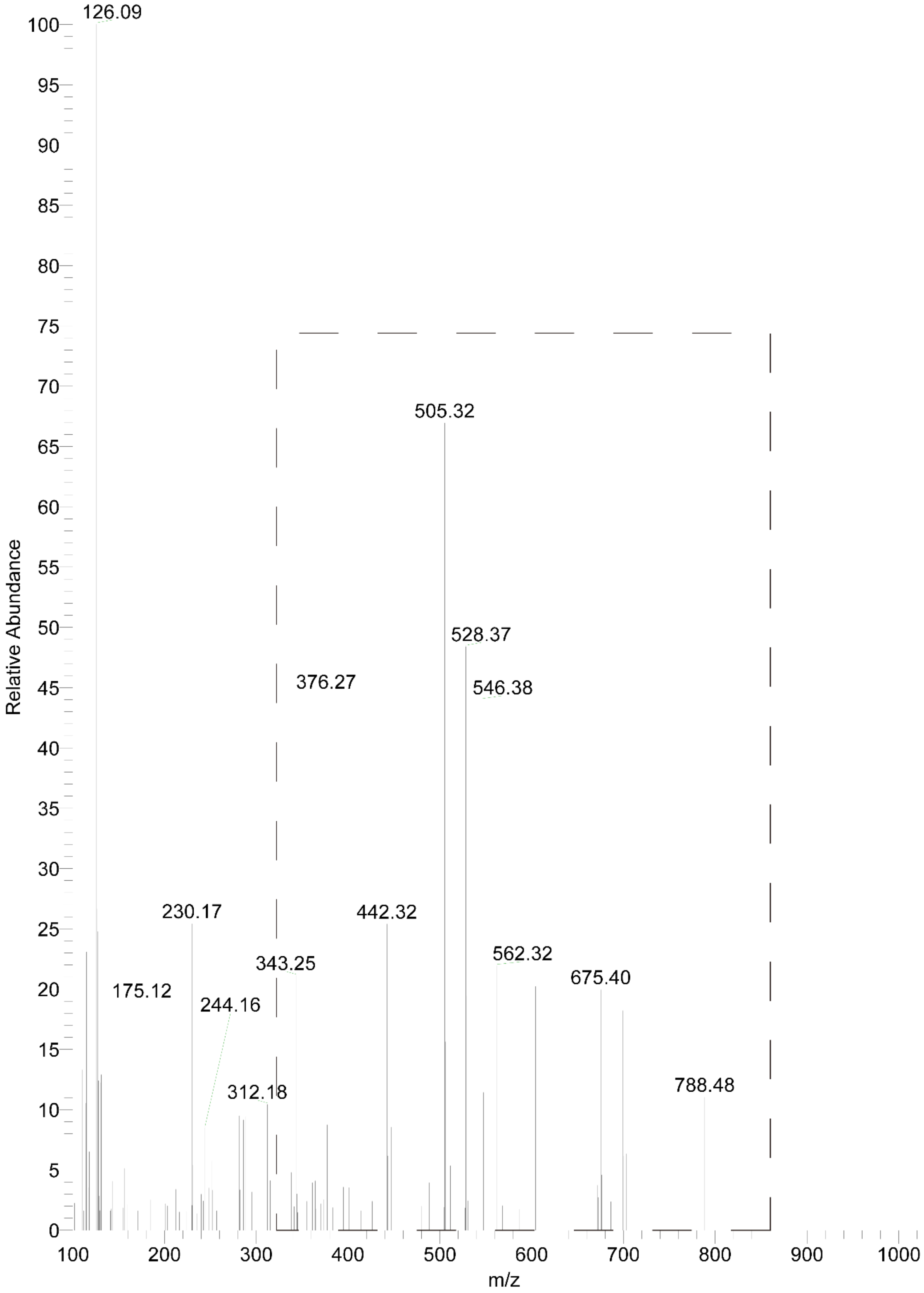

| Protein accession | Protein name | Protein ID | Modified sequence | Position | Charge | Mass error [ppm] | MS/MS Count |
|---|---|---|---|---|---|---|---|
| A0A3Q7EIF5 | PsbP | Solyc01g087040 | LYAHFVNAPAPEWK(1)K | 224 | 4 | -0.38168 | 2 |

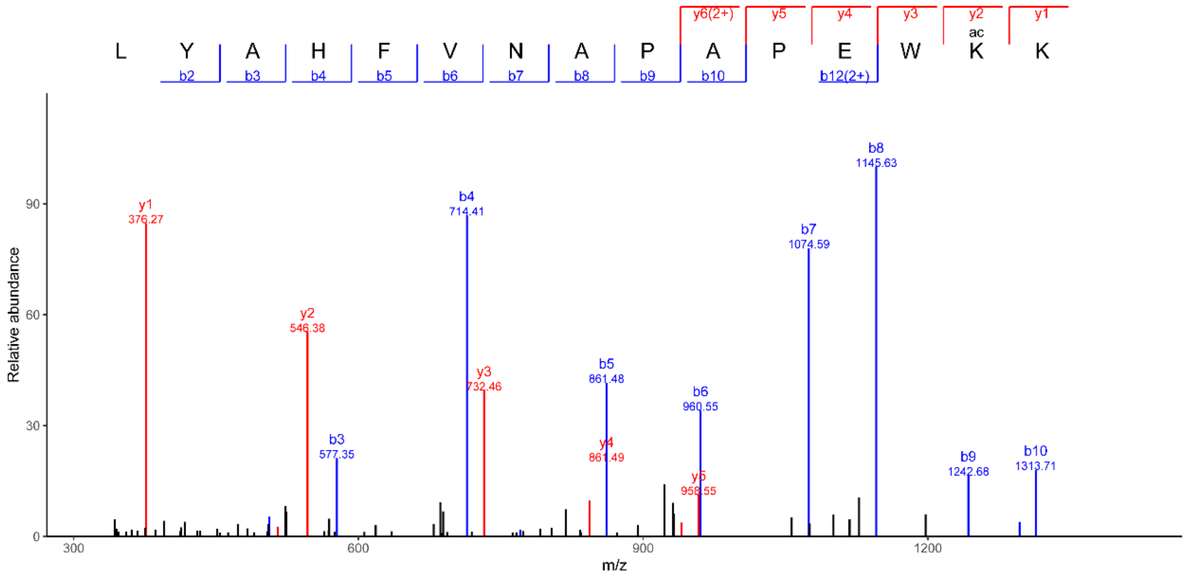


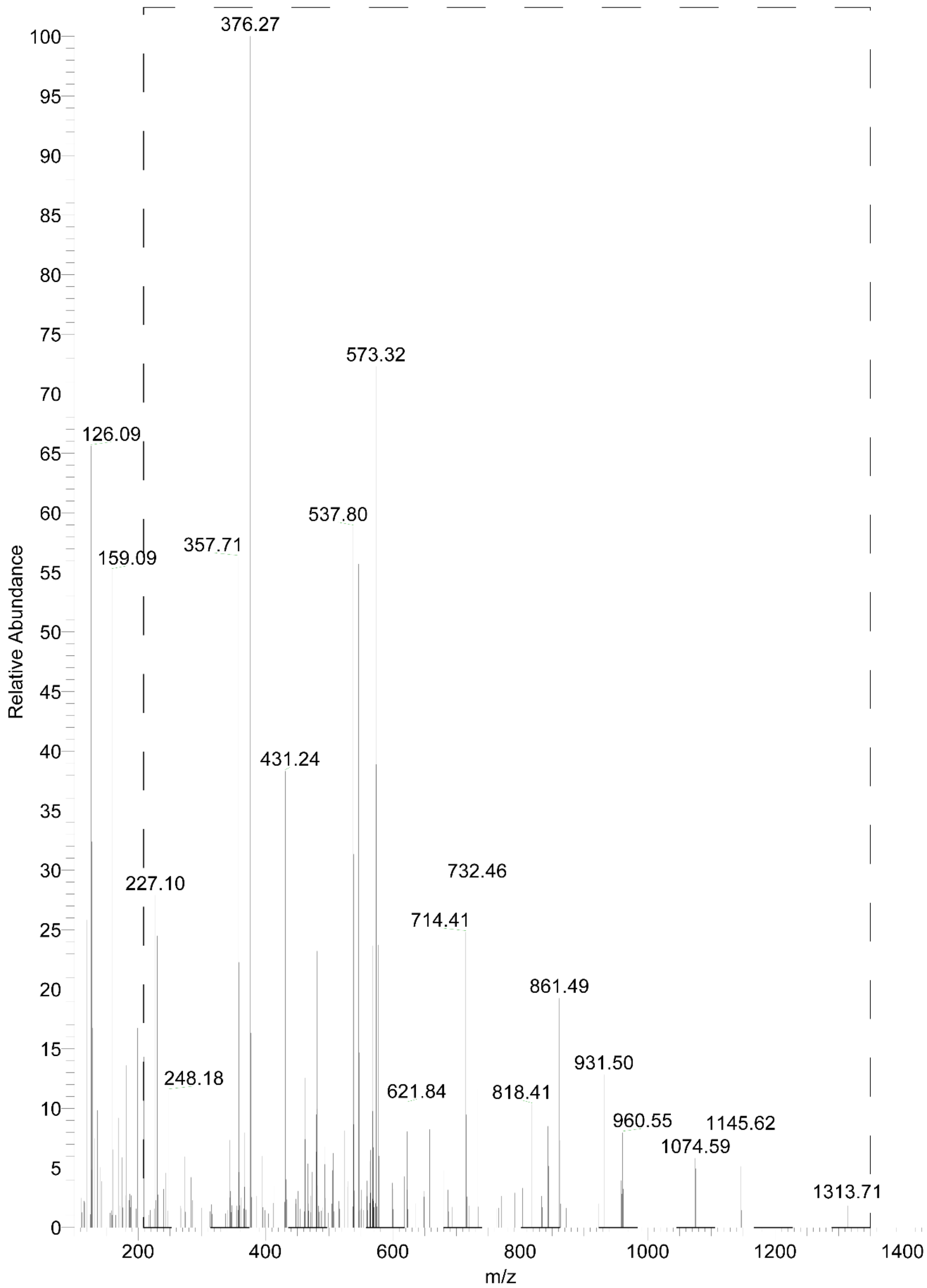

| Protein accession | Protein name | Protein ID | Modified sequence | Position | Charge | Mass error [ppm] | MS/MS Count |
|---|---|---|---|---|---|---|---|
| A0A3Q7G195 | Lhcb1 | Solyc03g005770 | AK(1)PASSGSPWYGPDR | 290 | 3 | -1.5254 | 2 |

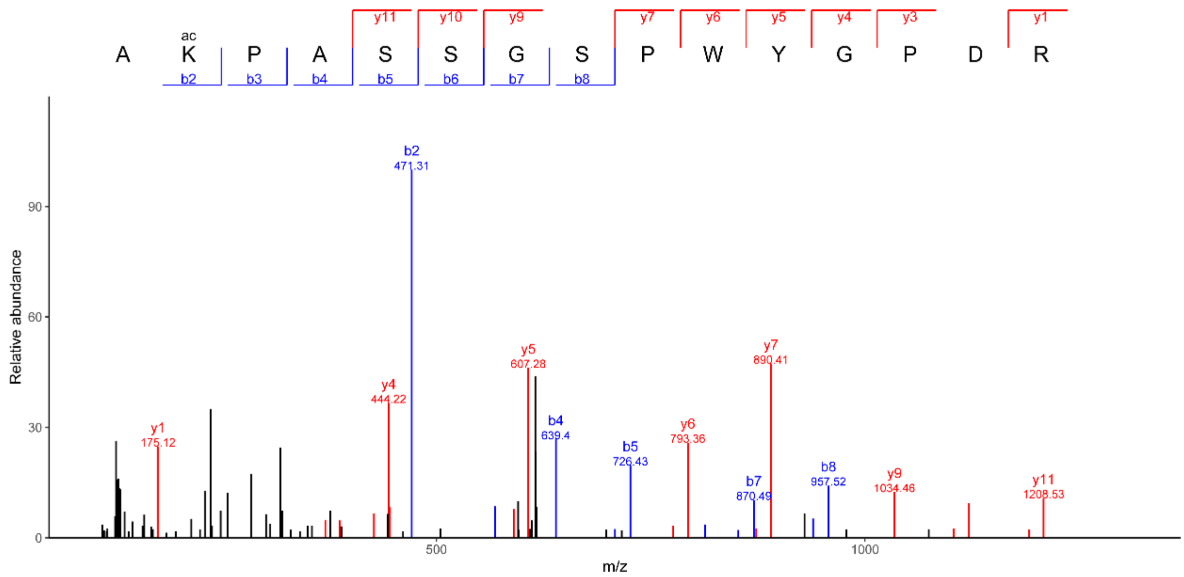


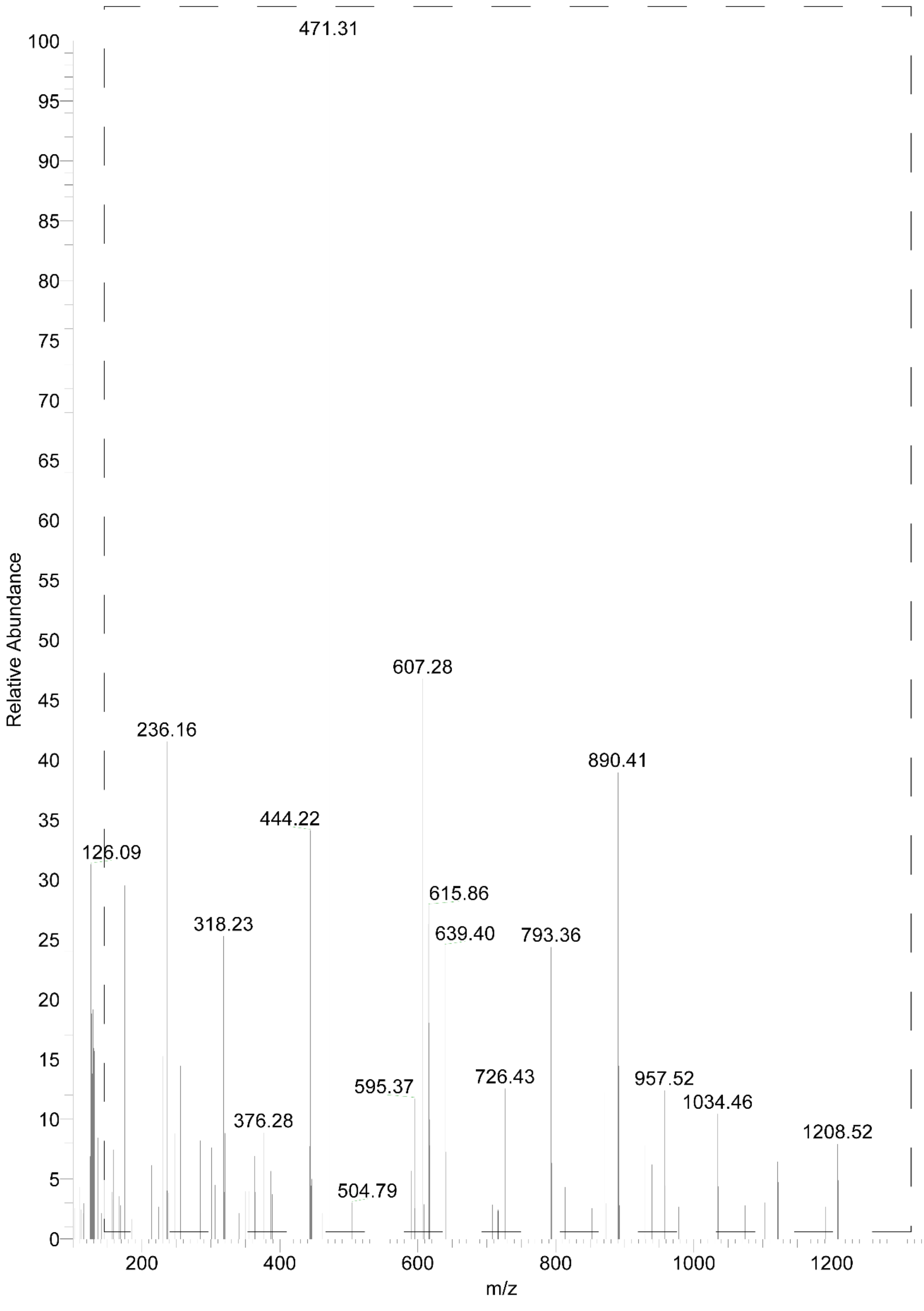

| Protein accession | Protein name | Protein ID | Modified sequence | Position | Charge | Mass error [ppm] | MS/MS Count |
|---|---|---|---|---|---|---|---|
| A0A3Q7HDJ6 | Lhcb2 | Solyc07g047850 | TVK(1)SAPQSIWYGEDRPK | 6 | 3 | -0.72614 | 18 |

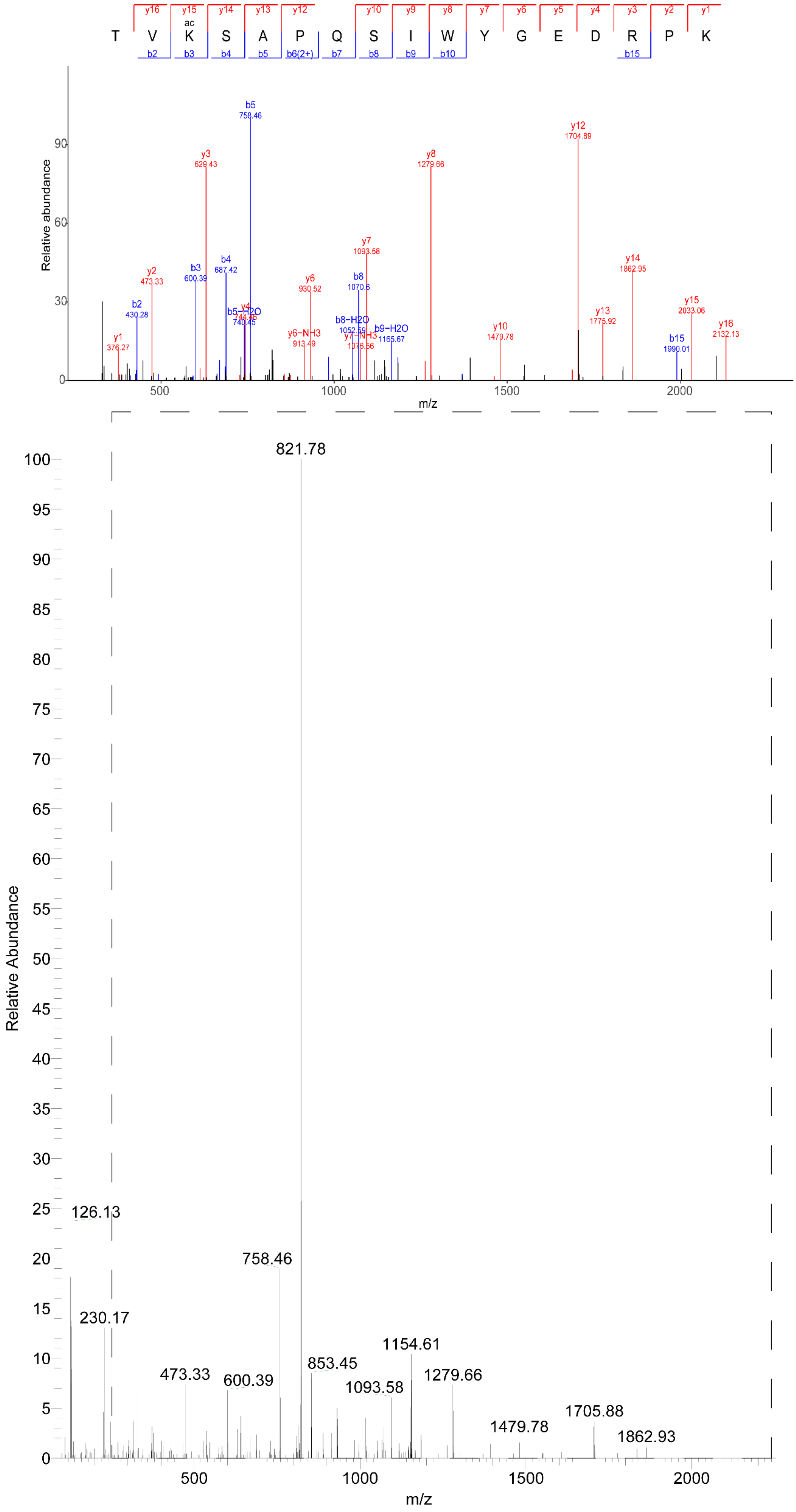

| Protein accession | Protein name | Protein ID | Modified sequence | Position | Charge | Mass error [ppm] | MS/MS Count |
|---|---|---|---|---|---|---|---|
| A0A3Q7I0X4 | Lhcb4 | Solyc09g014520 | FGFGQK(1)K | 34 | 3 | -0.067072 | 4 |

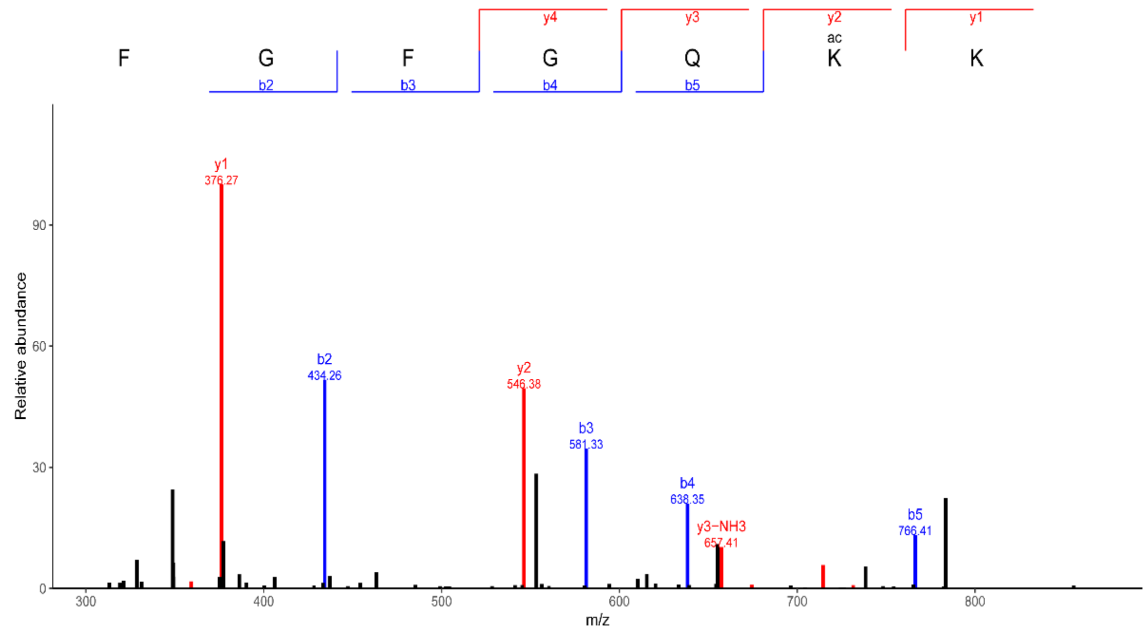


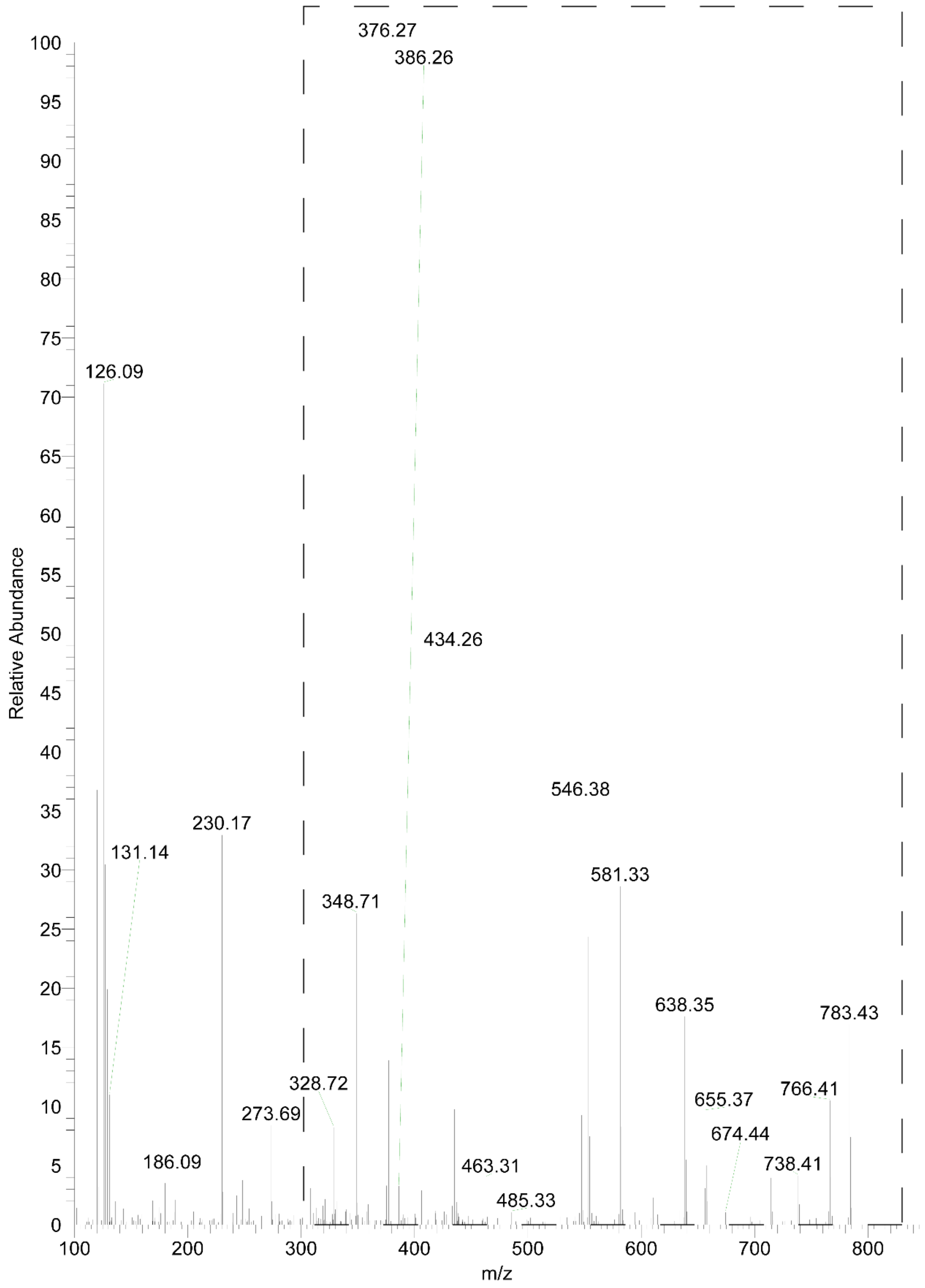

| Protein accession | Protein name | Protein ID | Modified sequence | Position | Charge | Mass error [ppm] | MS/MS Count |
|---|---|---|---|---|---|---|---|
| A0A3Q7IFE9 | PsaN | Solyc08g013670 | YK(1)CGSNVFWK | 154 | 2 | 4.1408 | 6 |

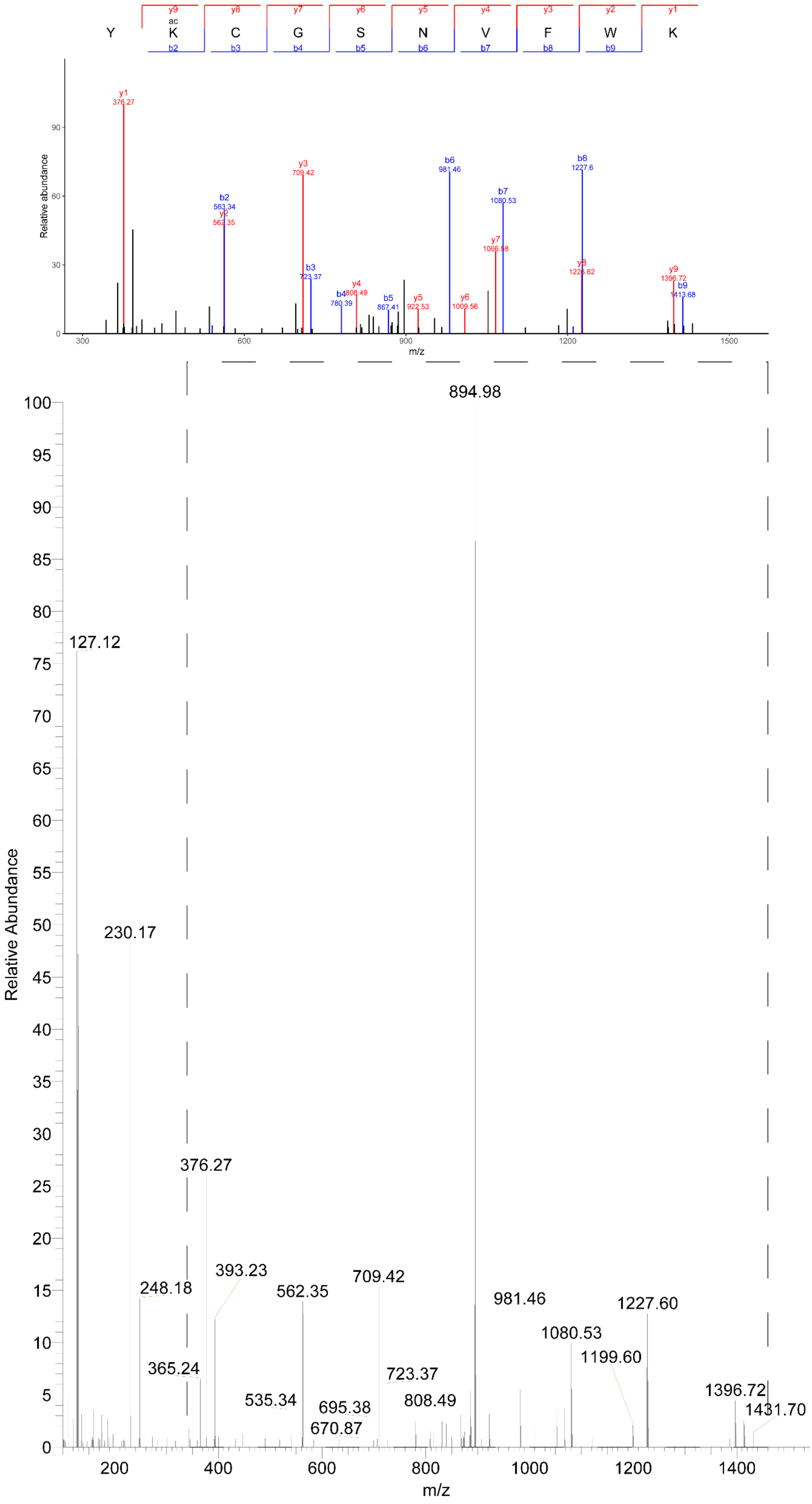

| Protein accession | Protein name | Protein ID | Modified sequence | Position | Charge | Mass error [ppm] | MS/MS Count |
|---|---|---|---|---|---|---|---|
| A0A3Q7IFE9 | PsaN | Solyc08g013670 | FPENFTGCQDLAK(1)QK(1)K | 132 | 3 | 0.30631 | 22 |

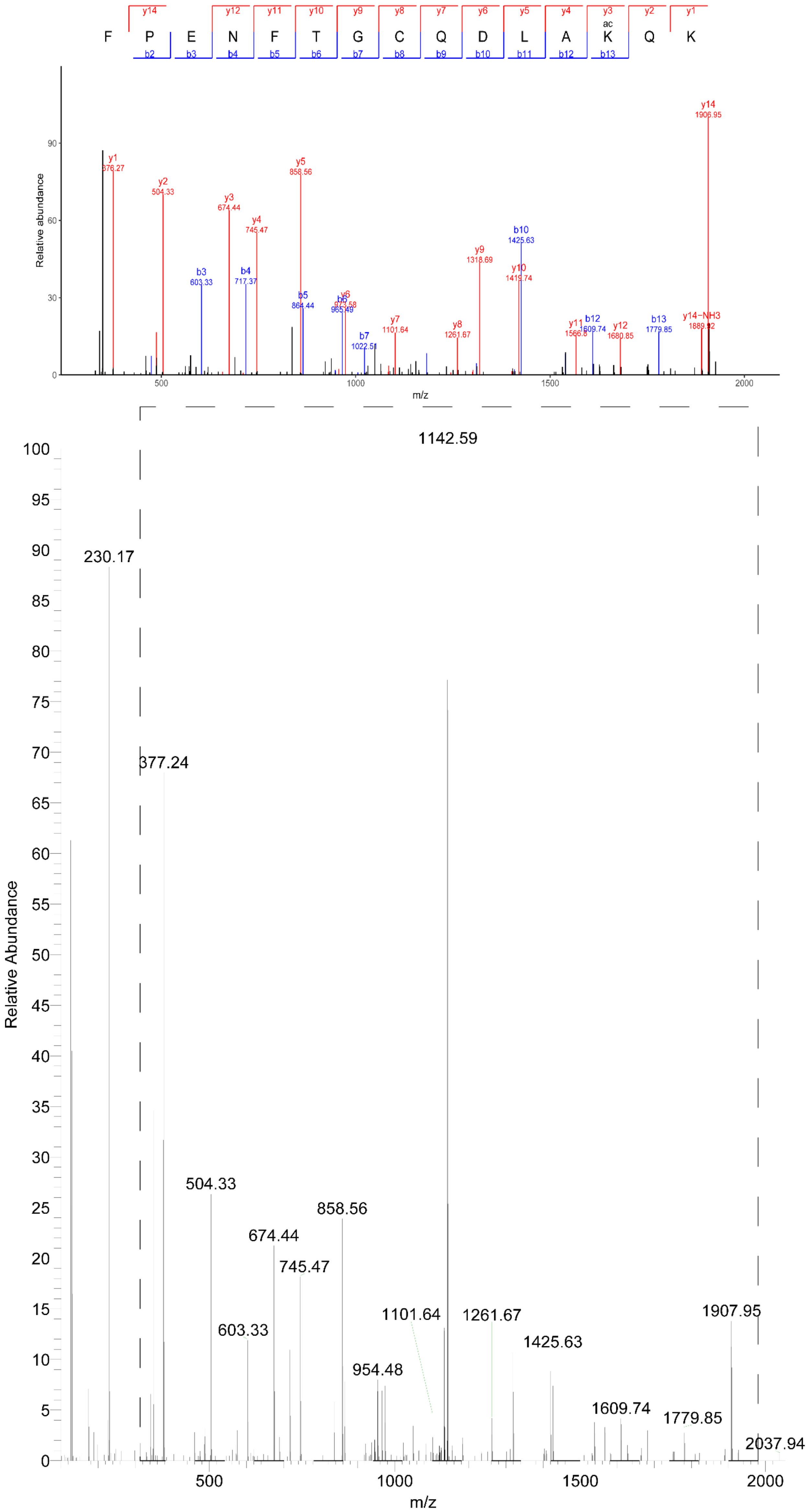

| Protein accession | Protein name | Protein ID | Modified sequence | Position | Charge | Mass error [ppm] | MS/MS Count |
|---|---|---|---|---|---|---|---|
| P23322 | PsbO | Solyc02g065400 | ENVK(1)NTASLTGK | 273 | 2 | -0.96567 | 7 |

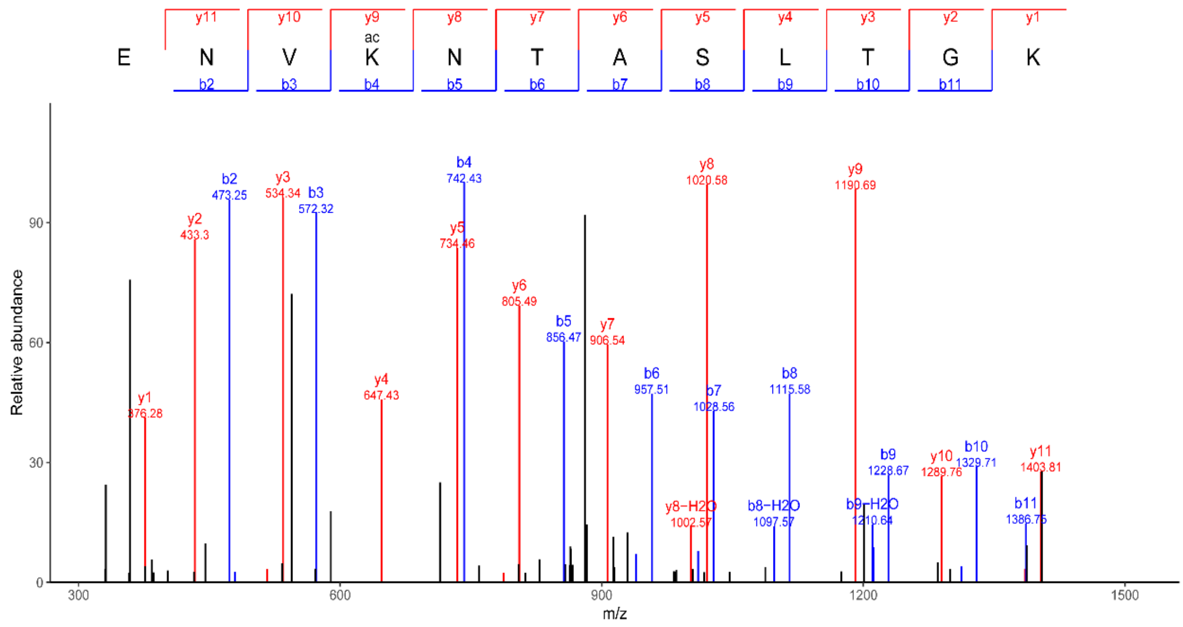


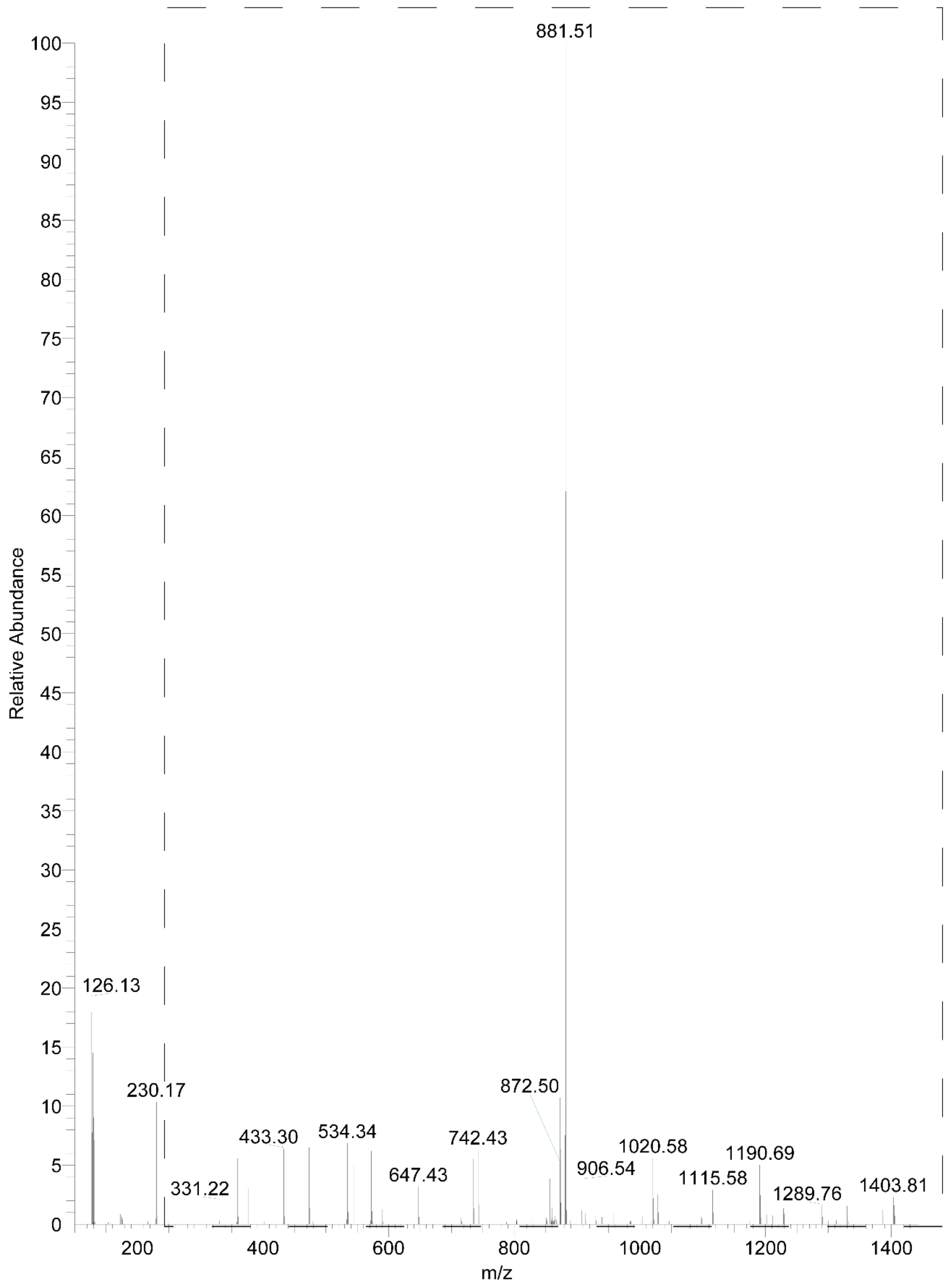

| Protein accession | Protein name | Protein ID | Modified sequence | Position | Charge | Mass error [ppm] | MS/MS Count |
|---|---|---|---|---|---|---|---|
| P23322 | PsbO | Solyc02g065400 | NSAPDFQK(1)TK | 157 | 3 | -0.24466 | 8 |

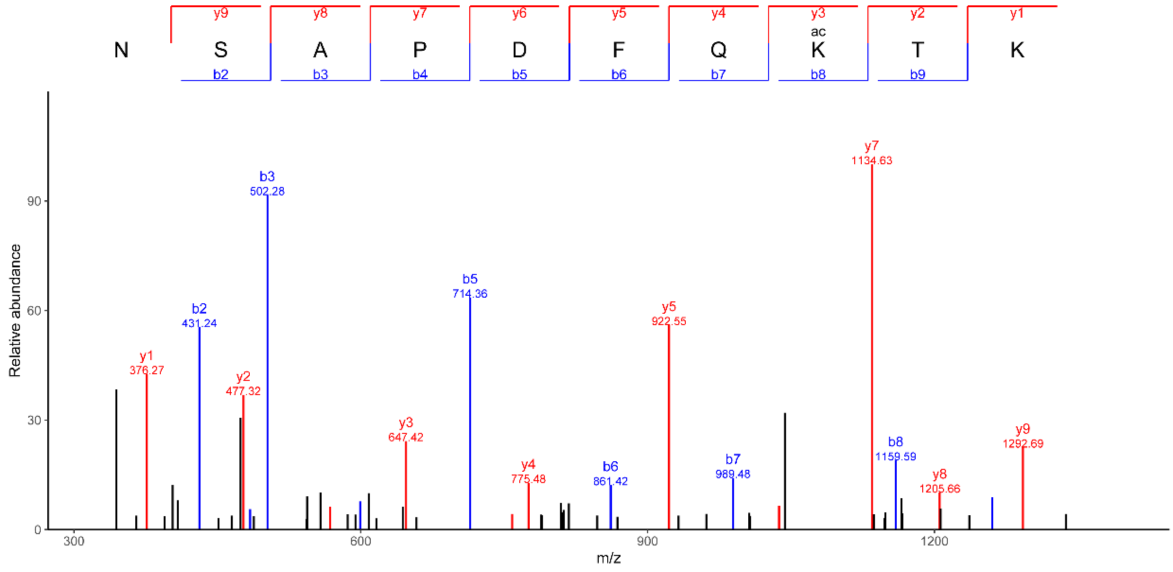


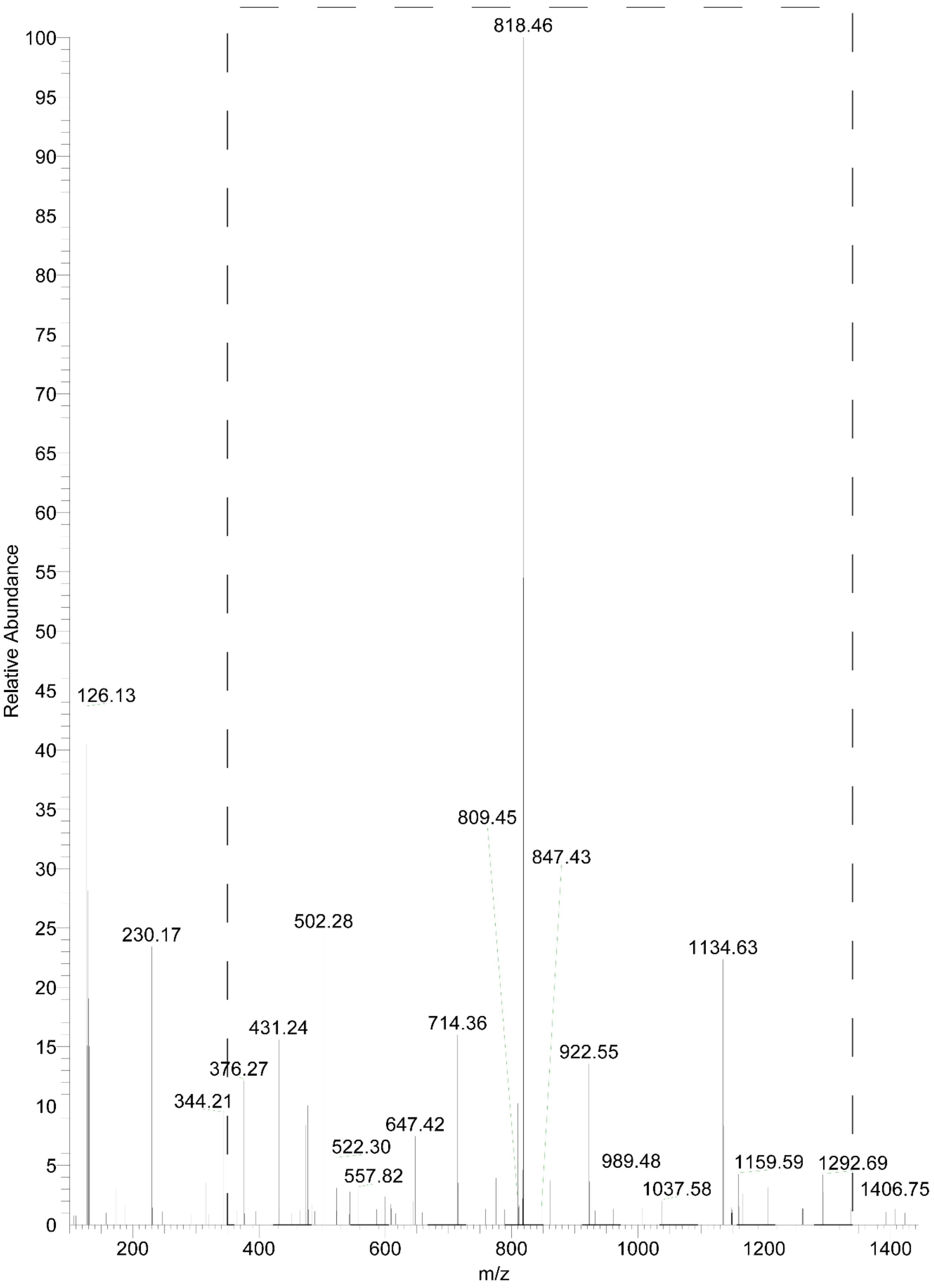

| Protein accession | Protein name | Protein ID | Modified sequence | Position | Charge | Mass error [ppm] | MS/MS Count |
|---|---|---|---|---|---|---|---|
| P31542 | CD4B | Solyc12g042060 | VPEPTVDETIQILK(1)GLR | 444 | 3 | 0.16519 | 1 |

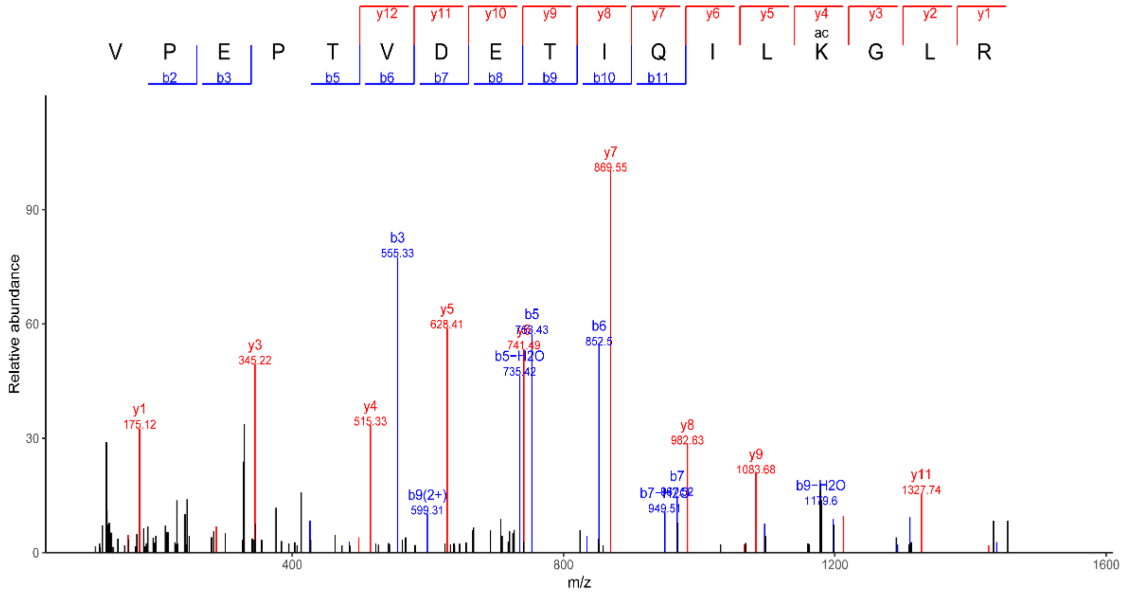


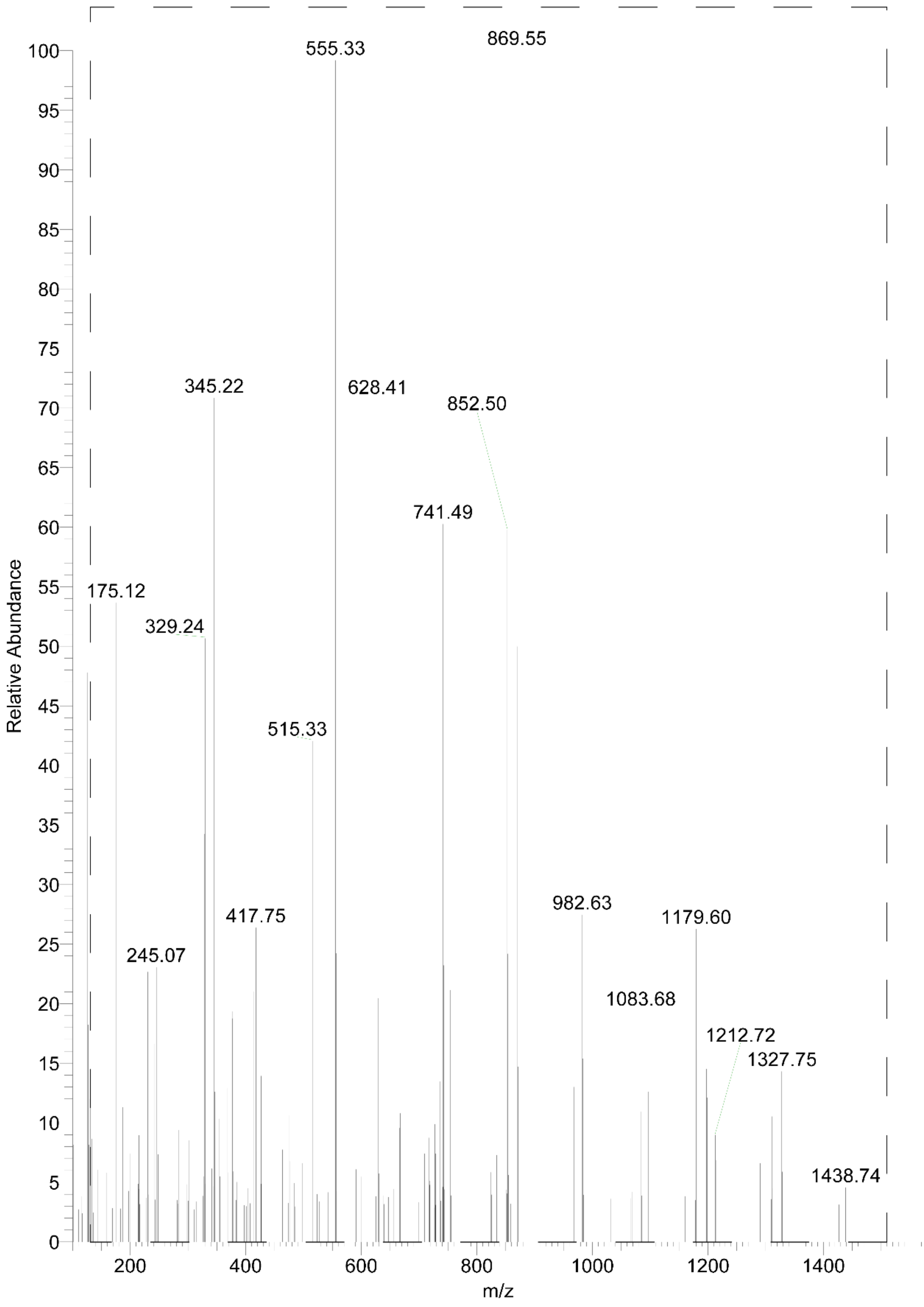

| Protein accession | Protein name | Protein ID | Modified sequence | Position | Charge | Mass error [ppm] | MS/MS Count |
|---|---|---|---|---|---|---|---|
| Q2MI75 | PsbB | Solyc01g007500 | LAFYDYIGNNPAK(1)GGLFR | 321 | 2 | -0.48466 | 2 |

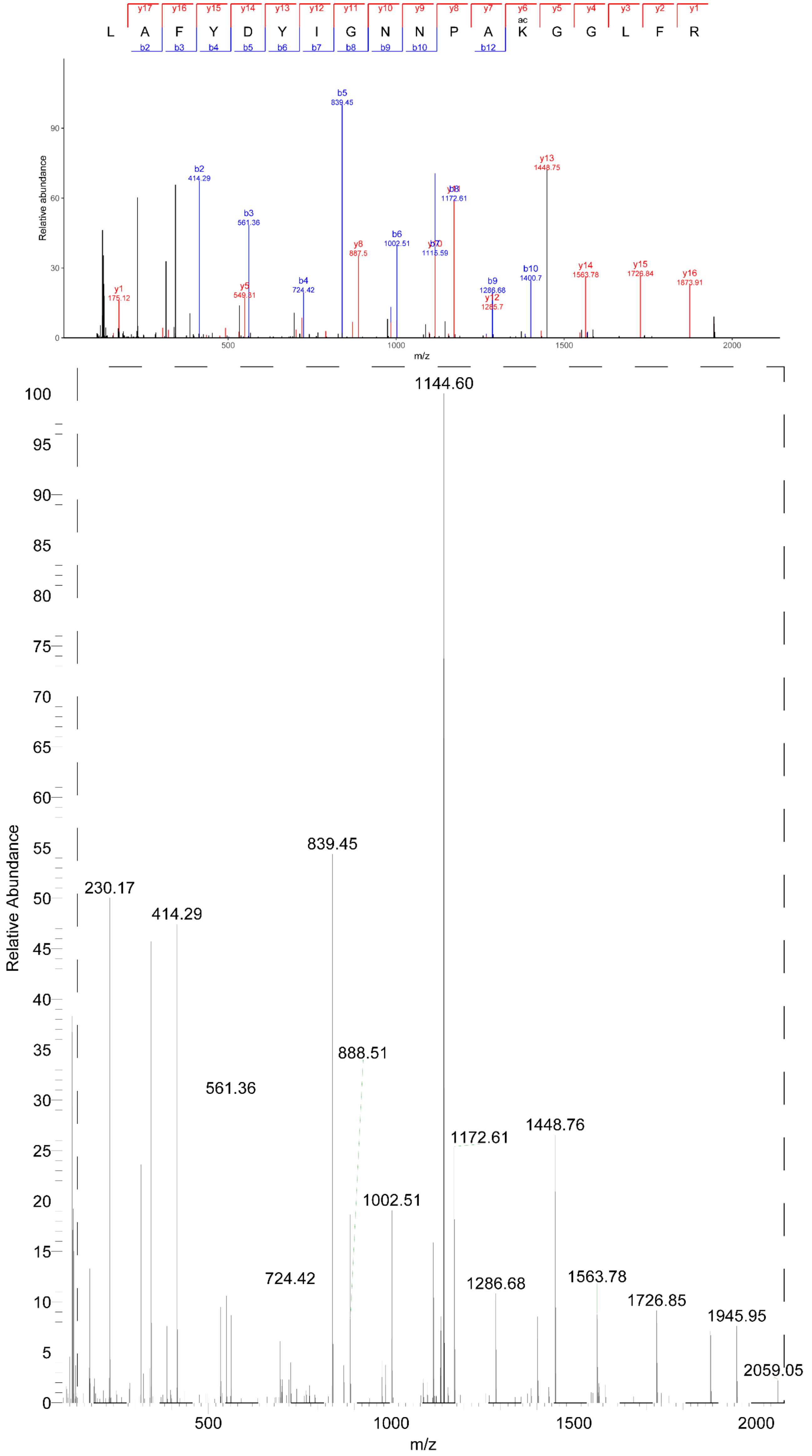

| Protein accession | Protein name | Protein ID | Modified sequence | Position | Charge | Mass error [ppm] | MS/MS Count |
|---|---|---|---|---|---|---|---|
| Q2MI75 | PsbB | Solyc01g007500 | VSAGLAENQSLSEAWSK(1)IPEK | 304 | 5 | 0.020452 | 4 |

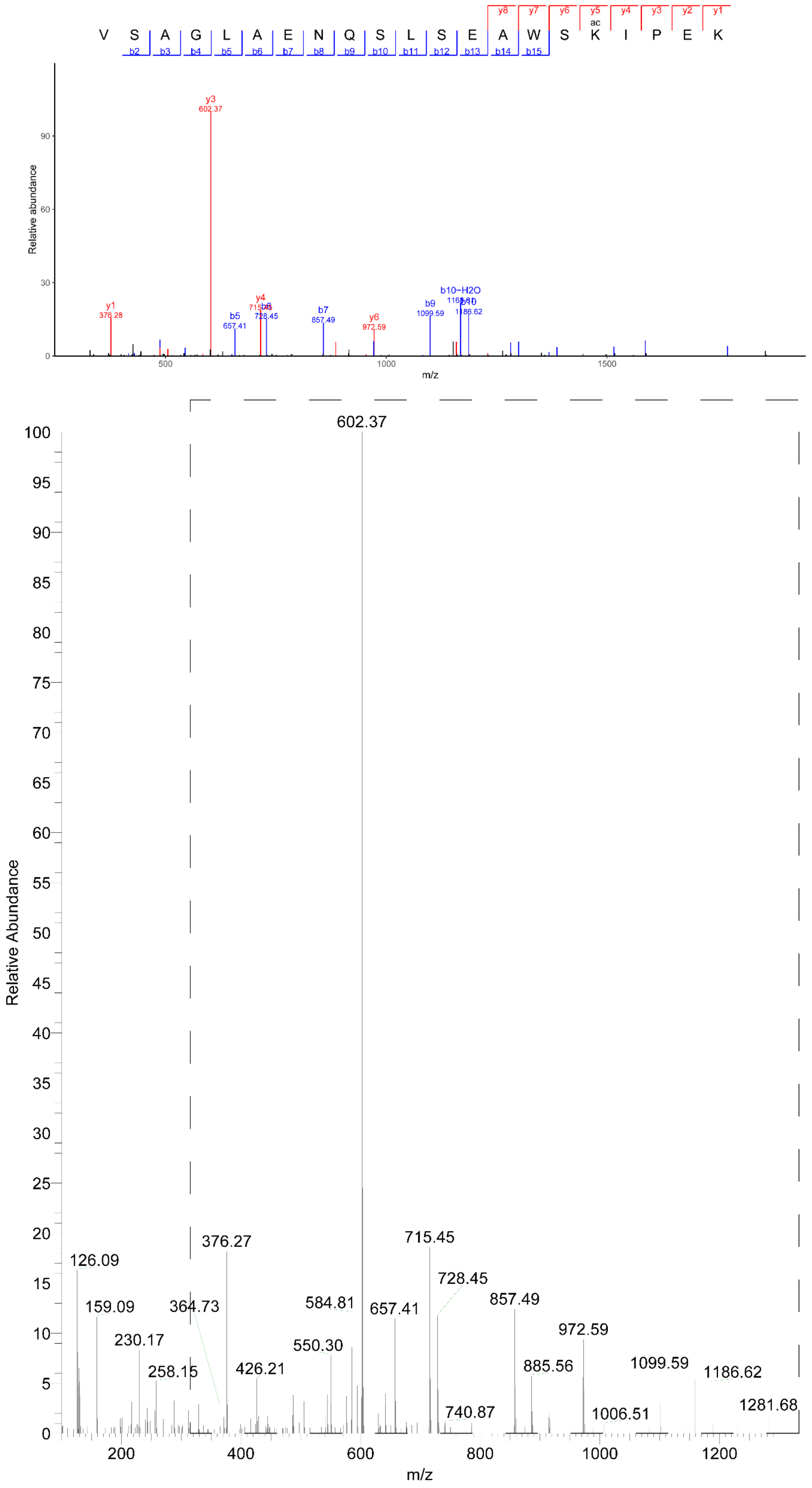

| Protein accession | Protein name | Protein ID | Modified sequence | Position | Charge | Mass error [ppm] | MS/MS Count |
|---|---|---|---|---|---|---|---|
| Q672Q6 | PsbQ | Solyc02g079950 | GK(1)LQDLSGK | 184 | 3 | -1.224 | 2 |

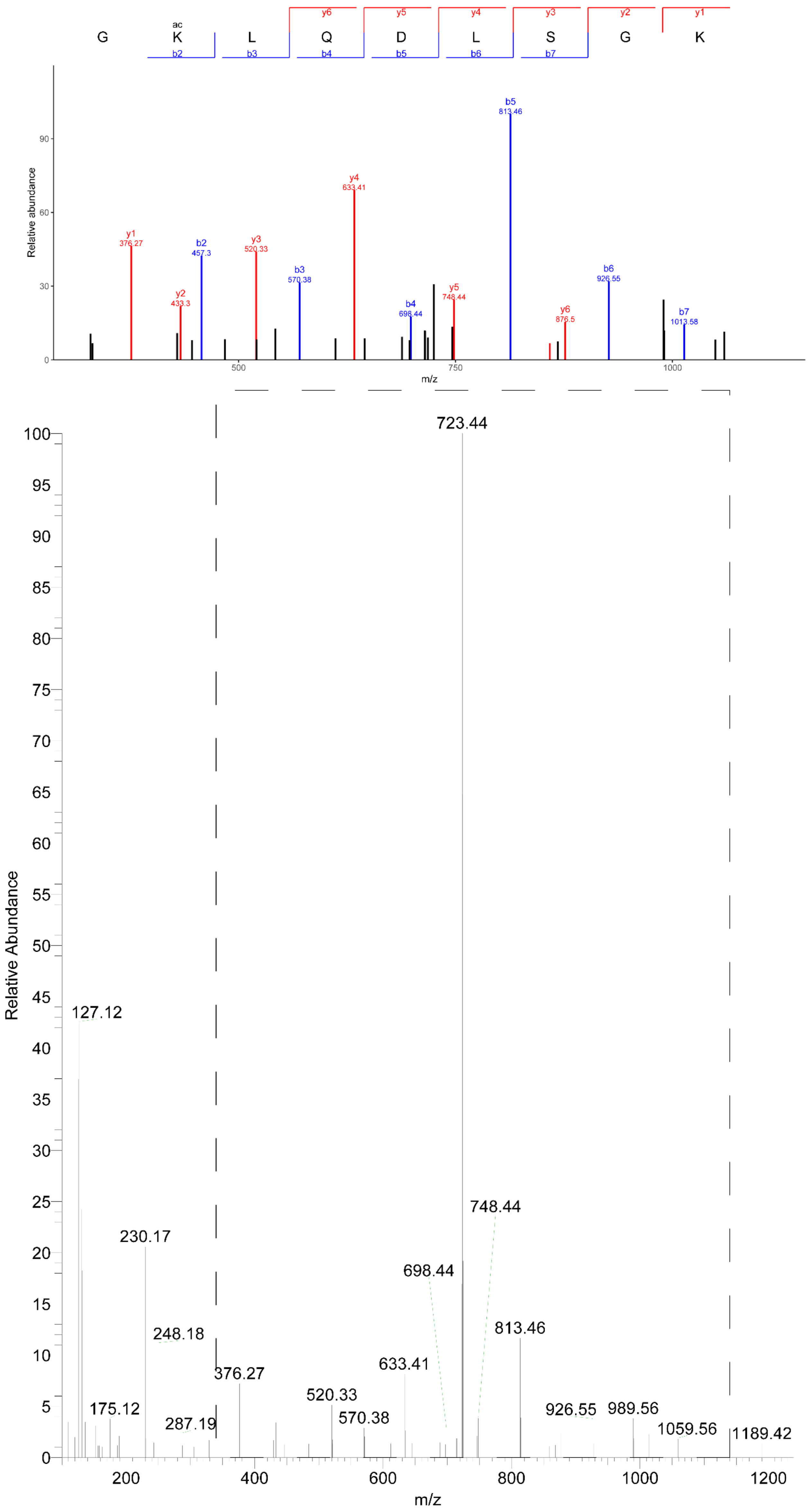

| Protein accession | Protein name | Protein ID | Modified sequence | Position | Charge | Mass error [ppm] | MS/MS Count |
|---|---|---|---|---|---|---|---|
| Q672Q6 | PsbQ | Solyc02g079950 | DFSLPLK(1)NR | 116 | 3 | -0.49362 | 3 |

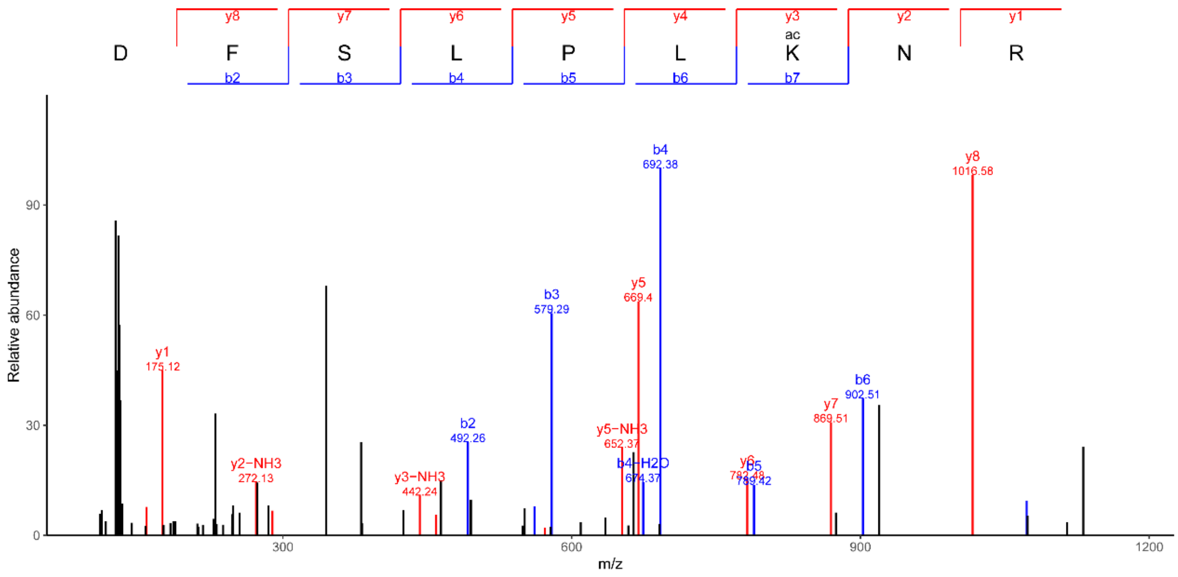


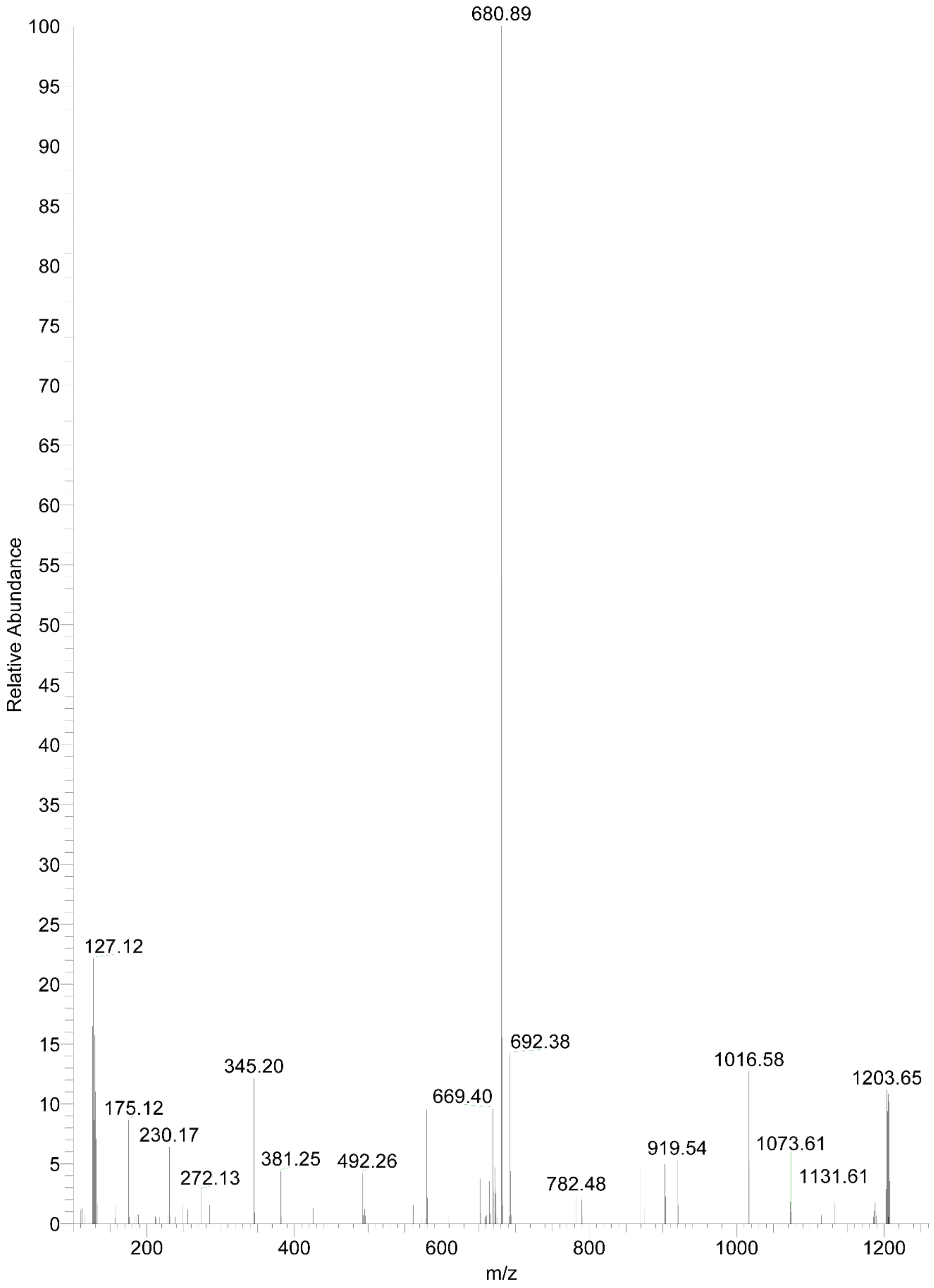

| Protein accession | Protein name | Protein ID | Modified sequence | Position | Charge | Mass error [ppm] | MS/MS Count |
|---|---|---|---|---|---|---|---|
| A0A3Q7GM40 | NDP | Solyc06g005710 | KPFASK(1)TK | 460 | 3 | 0.41856 | 2 |

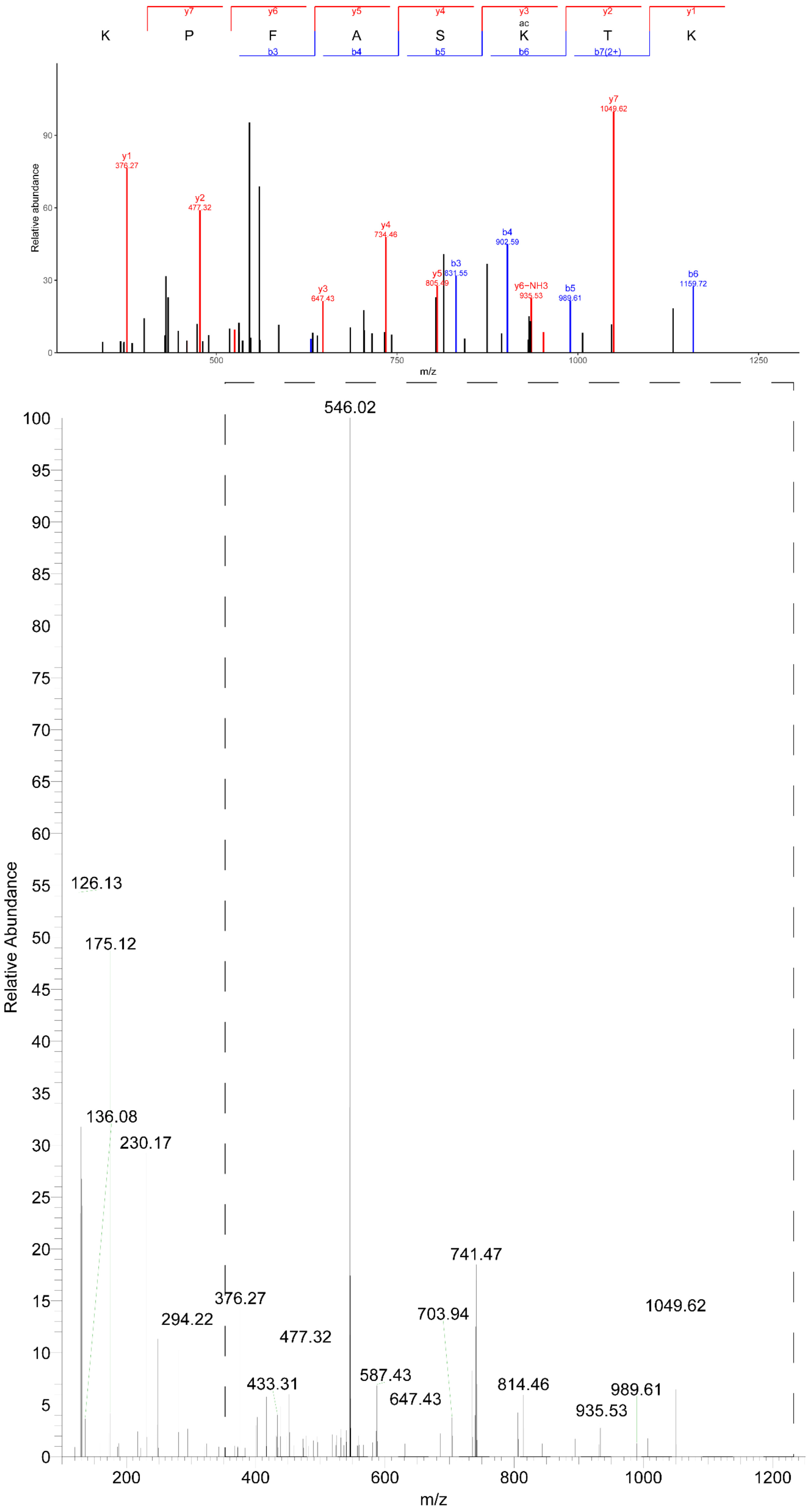

| Protein accession | Protein name | Protein ID | Modified sequence | Position | Charge | Mass error [ppm] | MS/MS Count |
|---|---|---|---|---|---|---|---|
| A0A3Q7GM40 | NDP | Solyc06g005710 | AK(1)NLPAQK | 440 | 3 | -0.4725 | 2 |

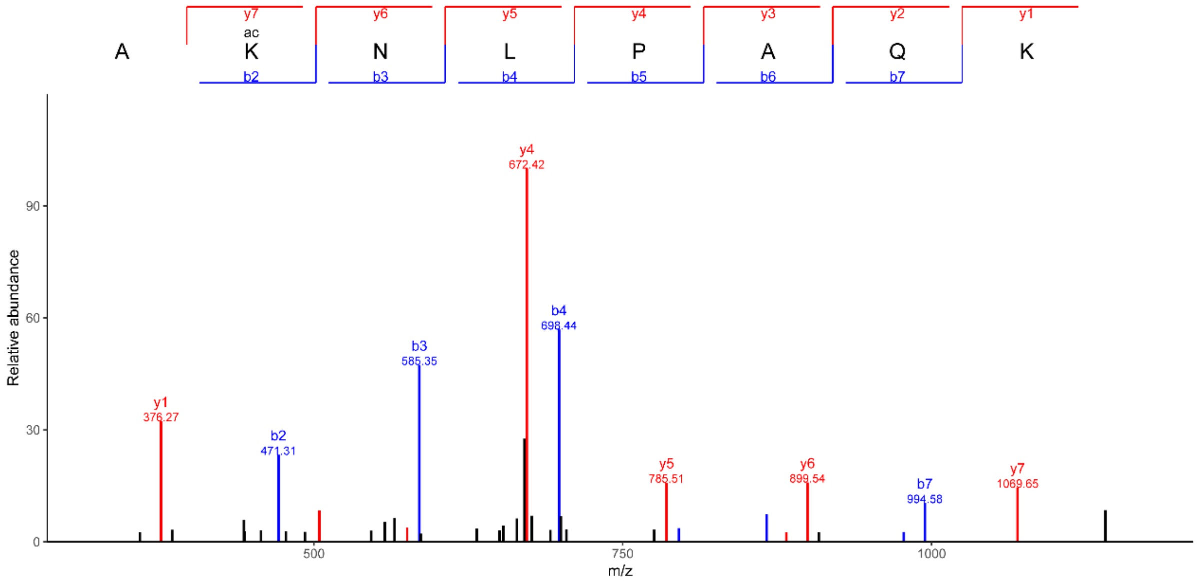


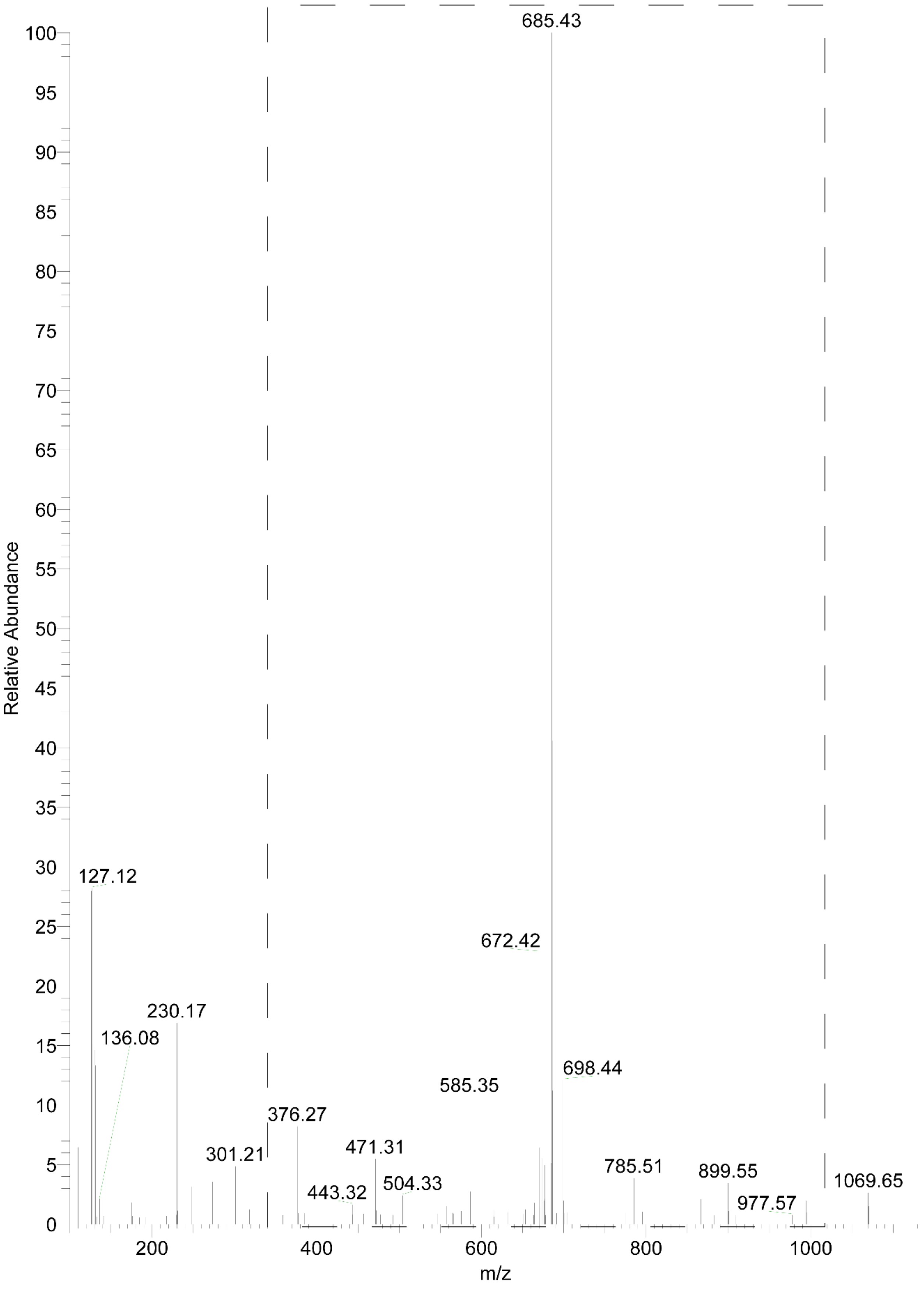

| Protein accession | Protein name | Protein ID | Modified sequence | Position | Charge | Mass error [ppm] | MS/MS Count |
|---|---|---|---|---|---|---|---|
| A0A3Q7GM40 | NDP | Solyc06g005710 | GVAEASK(1)R | 356 | 2 | -0.49552 | 1 |

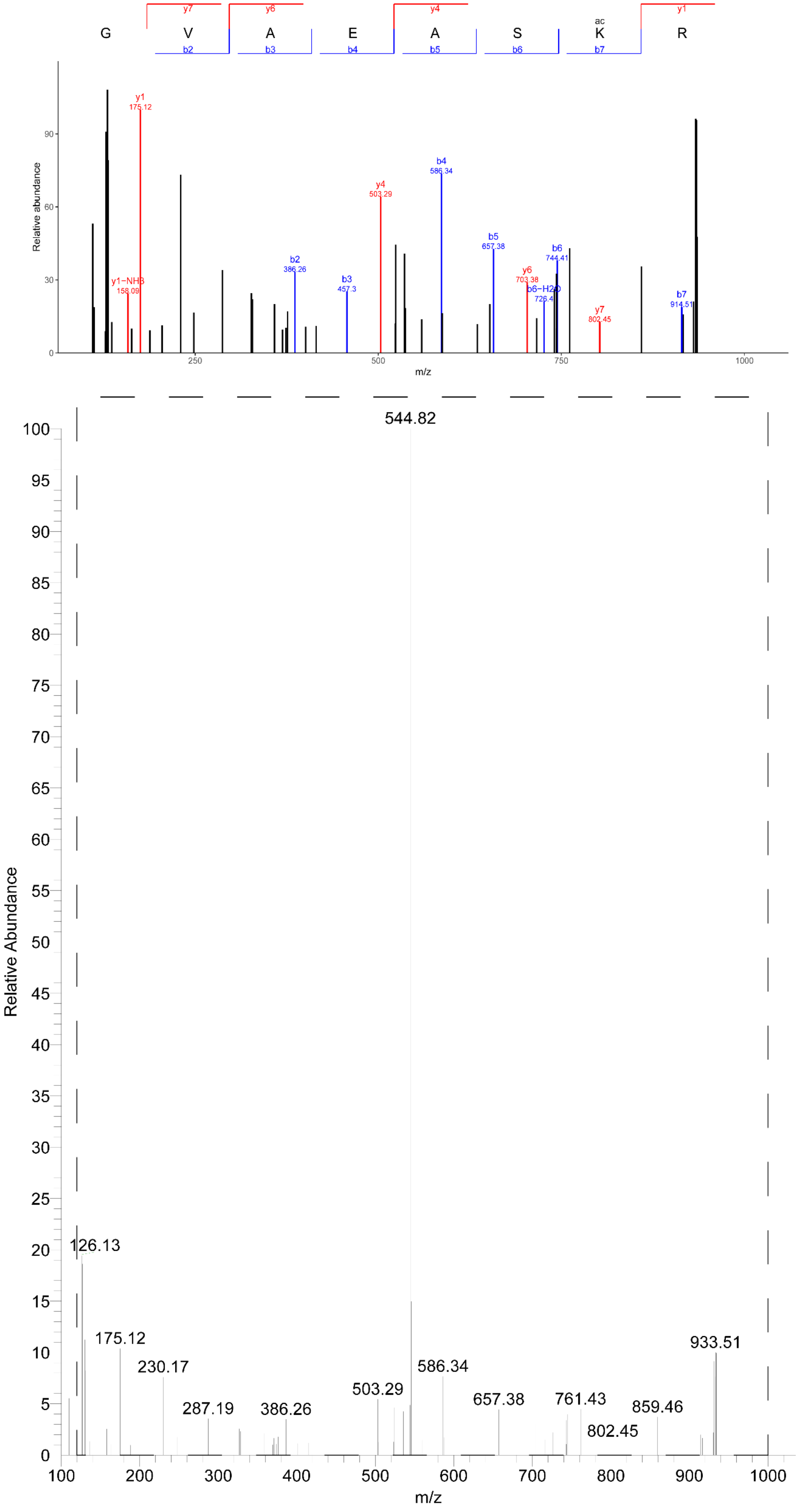

| Protein accession | Protein name | Protein ID | Modified sequence | Position | Charge | Mass error [ppm] | MS/MS Count |
|---|---|---|---|---|---|---|---|
| A0A3Q7GM40 | NDP | Solyc06g005710 | LSSQLK(1)SAVEK | 416 | 2 | 0.034715 | 2 |

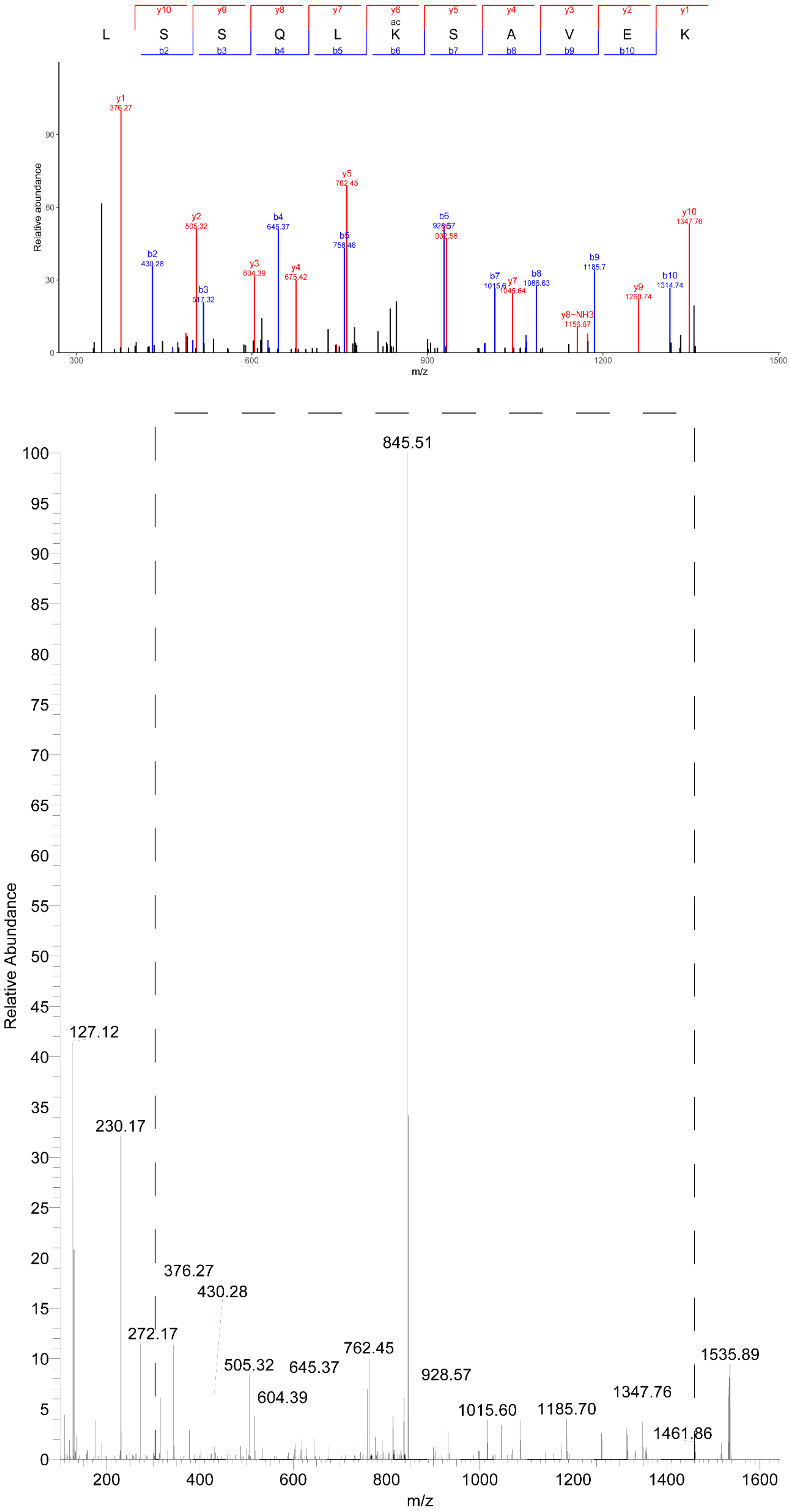

**B. Spectra of acetylated ΔLhcb2 peptides in mass spectrometry.**

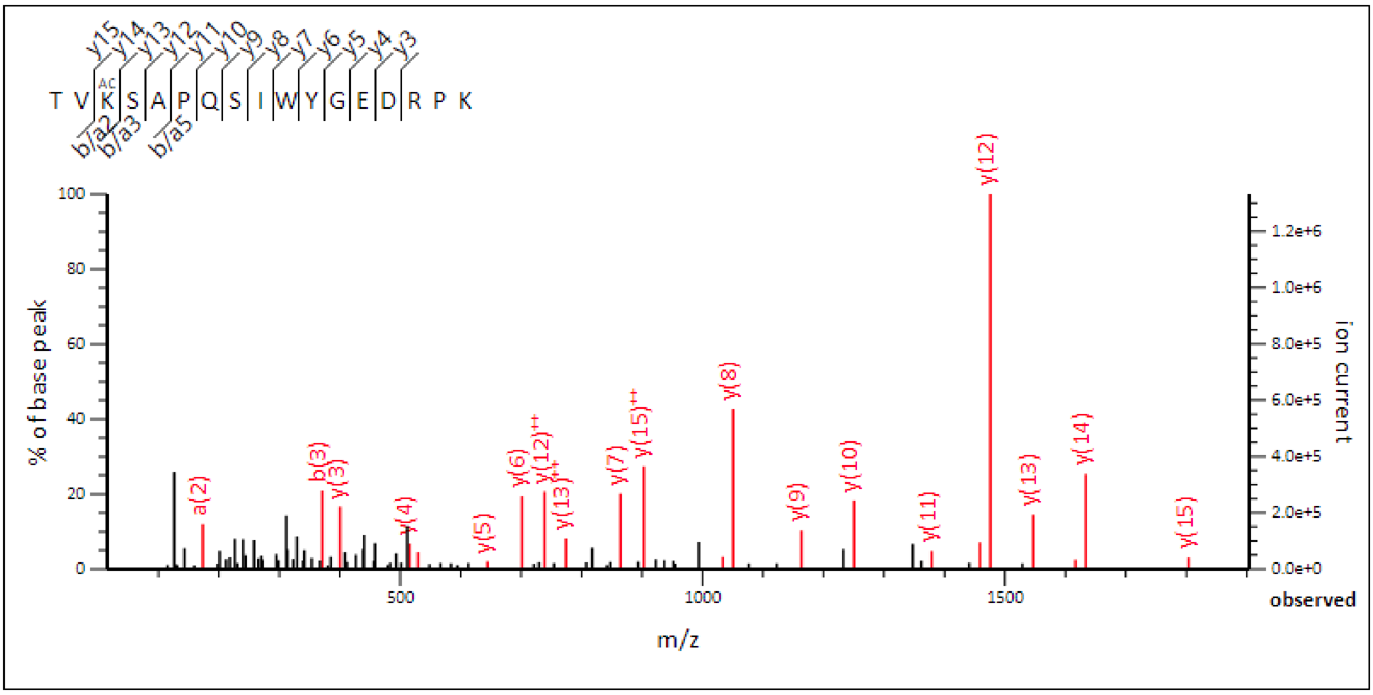

Supplement: Supplementary file 5 — Supplementary Material 5. [file 43897_2025_164_MOESM5_ESM.pdf]
